# Measuring juvenile habitat quality for fishes and invertebrates

**DOI:** 10.1111/brv.70050

**Published:** 2025-07-30

**Authors:** Benjamin J. Ciotti, Elliot J. Brown, Francesco Colloca, David B. Eggleston, A. Challen Hyman, Olivier Le Pape, Romuald N. Lipcius, Margot A. M. Maathuis, Suzanne S. H. Poiesz, Kenneth A. Rose, Rochelle D. Seitz, Daniele Ventura, Karen E. van de Wolfshaar

**Affiliations:** ^1^ University of Plymouth, Drake Circus Plymouth PL4 8AA UK; ^2^ National Institute of Aquatic Resources Technical University of Denmark (DTU Aqua) Kemitorvet, Building 201 Kongens Lyngby 2800 Denmark; ^3^ Department of Integrative Marine Ecology Stazione Zoologica Anton Dohrn Via G. Allegri 1 Rome 00198 Italy; ^4^ North Carolina State University Raleigh North Carolina 27695 USA; ^5^ Virginia Institute of Marine Science, William & Mary P.O. Box 1346 Gloucester Point Virginia 23062 USA; ^6^ DECOD (Ecosystem Dynamics and Sustainability), Institut Agro, INRAe, Ifremer 65 rue de Saint‐Brieuc Rennes Cedex 35042 France; ^7^ Aquaculture and Fisheries Group, Wageningen University Wageningen 6700 AH The Netherlands; ^8^ Wageningen Marine Research Haringkade 1 IJmuiden 1970 AB The Netherlands; ^9^ Department of Coastal Systems NIOZ Royal Netherlands Institute for Sea Research PO Box 59 Den Burg Texel 1790 AB The Netherlands; ^10^ Horn Point Laboratory University of Maryland Center for Environmental Science 2020 Horns Point Road Cambridge Maryland 21613 USA; ^11^ Department of Environmental Biology University of Rome ‘La Sapienza’ V. le dell'Università 32 Rome 00185 Italy

**Keywords:** ecosystem‐based fisheries management, essential fish habitat, MPA, nursery ground, recruitment, habitat suitability, managed realignment, restoration, larvae, subadult

## Abstract

Identifying the role of marine and estuarine habitats in supporting fish and invertebrate populations during vulnerable juvenile life stages is essential to achieve effective conservation and fisheries management. There remains general agreement that: (*i*) the quality of juvenile habitat is best measured as the contribution of juveniles to adult populations (here “juvenile–adult contribution”) and (*ii*) this contribution may be measured directly or inferred from habitat‐specific abundance, growth and survival. Obtaining effective estimates of juvenile habitat quality using these four metrics, however, is challenging. Through a systematic review of approaches to measure juvenile habitat quality, we critically evaluate current abilities to identify key habitats and provide recommendations for future work. We found that research in this area remains dominated by measurements of abundance (85% of studies) and, to a lesser extent growth (51% of studies), with limitations in the spatiotemporal resolution and extent of sampling. Relatively few approaches are available to measure survival and juvenile–adult contribution. Knowledge of juvenile habitat quality is further limited by restricted coverage of geographic areas, taxonomic groups and habitats. Based on our analysis of 874 studies over the past *ca.* 50 years, we provide five recommendations for enabling juvenile habitat research to support fisheries and conservation management better in future.

## I. INTRODUCTION

Coastal and estuarine ecosystems provide numerous ecological and socioeconomic functions (Costanza *et al*., 1997), which must be quantified and evaluated to reconcile conflicts among users of the coastal zone (Janssen *et al*., 2018). One important function is to support growth and development of juvenile life stages of exploited marine fish and invertebrate populations (Gibson, 1994). Juveniles typically occupy shallow, coastal and estuarine areas (Seitz *et al*., 2014) which may also be impacted by multiple human pressures (Brown *et al*., 2018). Identifying specific habitats that are essential for supporting juveniles is therefore a critical step in setting conservation priorities and delineating the natural and anthropogenic drivers of variation in marine populations (Beck *et al*., 2001; Hayes, Ferreri & Taylor, 1996; Lefcheck *et al*., 2019).

Habitat is a spatially and temporally defined area where an organism resides, including the abiotic and biotic environment (Begon, Townsend & Harper, 2006; Hayes *et al*., 1996). Exactly how best to measure the quality of habitats for juvenile fishes and invertebrates remains a matter of debate. Initially, juvenile fish habitat quality was inferred based on loosely defined criteria, often relating to the abundance of juveniles it contained (Beck *et al*., 2001). Beck *et al*. (2001, p. 635), however, formalised the Nursery Role Hypothesis by defining an area as a nursery for a particular species if its “contribution per unit area to the production of individuals that recruit to adult populations is greater, on average, than production from other habitats in which juveniles occur”. This definition distinguished “nurseries” as areas of particular importance, within the broader pool of “juvenile habitats”. Furthermore, the definition provided a metric to measure and compare the quality of habitats based on their contribution to the adult population (hereafter referred to as “juvenile–adult contribution”). While juvenile–adult contribution was recognised as the best way to quantify habitat quality, Beck *et al*. (2001) also identified four factors that support this contribution: abundance, growth and survival in the juvenile habitat as well as movement from the juvenile to adult habitat. In practice, measurements of movement are rarely separated from the overall contribution rate, so the current review focuses on four principal metrics of habitat quality: abundance, growth, survival and juvenile–adult contribution. The definition and four metrics of habitat quality provide a standard framework by which the quality of juvenile habitat can be rigorously measured, compared and categorised.

In the two decades since publication, the Nursery Role Hypothesis has received substantial scrutiny. Relatively minor modifications were suggested by Dahlgren *et al*. (2006) and Fodrie, Levin & Lucas (2009), who chiefly suggested that contributions overall, rather than contributions per unit area, were a more convenient and management‐relevant basis for quantifying the relative importance of habitats. Further adaptations were suggested to account for unique biological features of specific taxa such as sharks (Heupel, Carlson & Simpfendorfer, 2007). The Nursery Role Hypothesis was also regarded by some as too simplistic (Sheaves, Baker & Johnston, 2006; Sheaves *et al*., 2015), but few workable alternatives were offered (Layman *et al*., 2006) until Nagelkerken *et al*. (2015) proposed the seascape nursery concept. This conceptualises a nursery as “a spatially explicit seascape consisting of multiple mosaics of habitat patches that are functionally connected” (Nagelkerken *et al*., 2015, p. 362). The seascape nursery concept encouraged a move away from habitats as static, individual, homogenous entities. Instead, it emphasised how spatial characteristics of habitats influence their quality, incorporating habitat shifts, animal movement and spatially explicit usage of patches and corridors. The types of seascape properties identified as important included: suitability for settlement, linkages between habitats and connecting migration corridors used at different scales (diel, tidal, seasonal and ontogenetic habitat shifts), habitat mosaics with hotspots of abundance within a habitat (e.g. distance to marsh edge, distance to estuary mouth) and ecosystem corridors providing organisms with specific routes back to the adult population. More recently, Lefcheck *et al*. (2019) identified conflicts between research realism and management pragmatism, noting that the simpler Nursery Role Hypothesis framework for assessing nursery habitat was not being met and the more complex seascape framework was even further beyond the reach of practical implementation in monitoring and management.

Although many aspects of the Nursery Role Hypothesis have been contested, its core framework for measuring juvenile habitat quality remains widely agreed. Debate has centred on how to use estimates of juvenile habitat quality to identify “nurseries” (Sheaves *et al*., 2006, 2015). Disagreements have arisen from inconsistences in terminology: objections that the Nursery Role Hypothesis overlooked spatial context or eco‐physiological factors (e.g. temperature and salinity gradients; Sheaves *et al*., 2006) were founded more on different interpretations of habitat descriptors rather than on the fundamental approach to measure habitat quality. Furthermore, while the seascape nursery concept highlights important points about the scales and complexity of habitat use (Nagelkerken *et al*., 2015), widening the suite of factors that may determine habitat quality, it does not fundamentally alter approaches to its measurement. On this, there is widespread agreement: juvenile habitat quality is best measured as juvenile–adult contribution (Nagelkerken *et al*., 2015), and should be accompanied by understanding of the component processes (i.e. abundance, growth, survival) that drive it (Sheaves *et al*., 2015). Therefore, a consistent framework for measuring habitat quality remains, based on the four key “metrics” of Beck *et al*. (2001): abundance, growth, survival and juvenile–adult contribution.

The fundamental approach to measuring juvenile habitat quality is well established, but incorporating complexities of the seascape nursery concept requires greater consideration of spatial and temporal measurement scales. The seascape concept demands finer resolution in patterns of habitat quality (multiple life‐history sub‐stages, microhabitats) and consideration of additional environmental drivers (e.g. area, physicochemical gradient, microhabitat, emergent landscape characteristics, trophic productivity). Habitat quality must therefore be measured at temporal and spatial scales reflecting fine‐scale processes and localised movements occurring over diel and tidal scales, as well as the broader suite of habitats across which an individual moves from settlement to adulthood (MacPherson, 1998). This level of understanding can only be achieved with tools to measure abundance, growth, survival and juvenile–adult contribution across a range of spatial and temporal scales. But do such tools exist, are they routinely applied, and how can critical data gaps be overcome in future? This paper reviews the methods that have been used to measure juvenile habitat quality, with a focus on the four key metrics (abundance, growth, survival and juvenile–adult contribution), in order to: (*i*) understand how juvenile habitat quality is being measured and how approaches have varied over time, geographically, among taxa and among habitats; (*ii*) evaluate the ability of these methods to measure juvenile habitat quality at appropriate spatial and temporal scales; and (*iii*) use the answers to aims 1 and 2 to provide recommendations for measuring juvenile habitat quality.

## II. REVIEW AND EVALUATION OF CURRENT PRACTICE

### (1) Metrics of juvenile habitat quality

We performed a systematic review of 874 studies that have sought to measure juvenile habitat quality in marine or estuarine fish, crustaceans and molluscs (see online Supporting Information, Appendix S1 for methods and Fig. S1 for PRISMA flow diagram; publications included in the qualitative synthesis are listed in Appendix S2). Since we wanted to assess methods that had been deliberately chosen to measure habitat quality, we excluded papers where this was not the main intention of the study (see Fig. S1 for list of exclusion criteria). As the emphasis of this review is on methods to measure juvenile habitat quality, papers were excluded that did not involve data collection (i.e. purely review, theoretical, method development or modelling studies). The review focuses on methods used to measure juvenile habitat quality for single species, so we excluded those focusing on community‐ or assemblage‐level metrics (e.g. diversity or assemblage structure). In line with the scope of the review, we excluded papers that did not focus on marine or estuarine fish, crustaceans or molluscs. We excluded salmonids since they have a unique life history, often inhabit fresh water during juvenile stages and are the subject of a large and somewhat independent body of research. The emphasis of the review is on methods to measure juvenile habitat quality *in situ*, so papers that studied populations in the laboratory were also excluded.

**Fig. 1:**
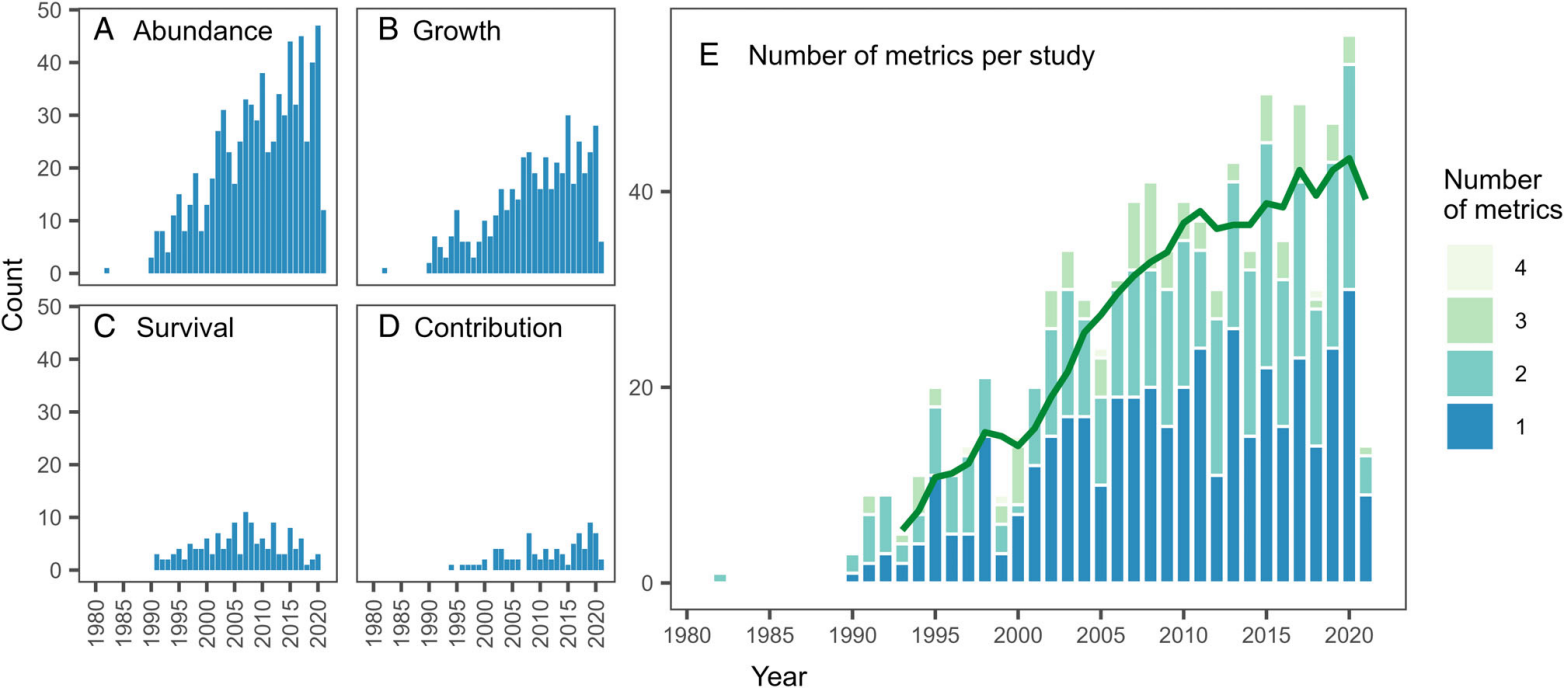
Annual frequencies of papers published since 1970 utilising the four different metrics of juvenile habitat quality suggested by Beck *et al*. (2001): (A) abundance, (B) growth, (C) survival and (D) juvenile–adult contribution. Bar heights represent the total number of papers published in a given year. (E) Annual sum of published studies separated according to the number of metrics employed concurrently in each paper. The green line represents the 5‐year simple moving average of the total number of papers published in a given year. Note that 2021 is an incomplete year due to the timing of the literature search.

Although our search included literature from 1970 onwards, the first paper describing juvenile habitat metrics was published in 1982 (Rosenberg, 1982). We found no relevant papers published in the following 8 years, after which there was an increase from *ca*. 10 per annum in the 1990s to *ca*. 40 per annum in the 2010s (Fig. 1).

**Fig. 2:**
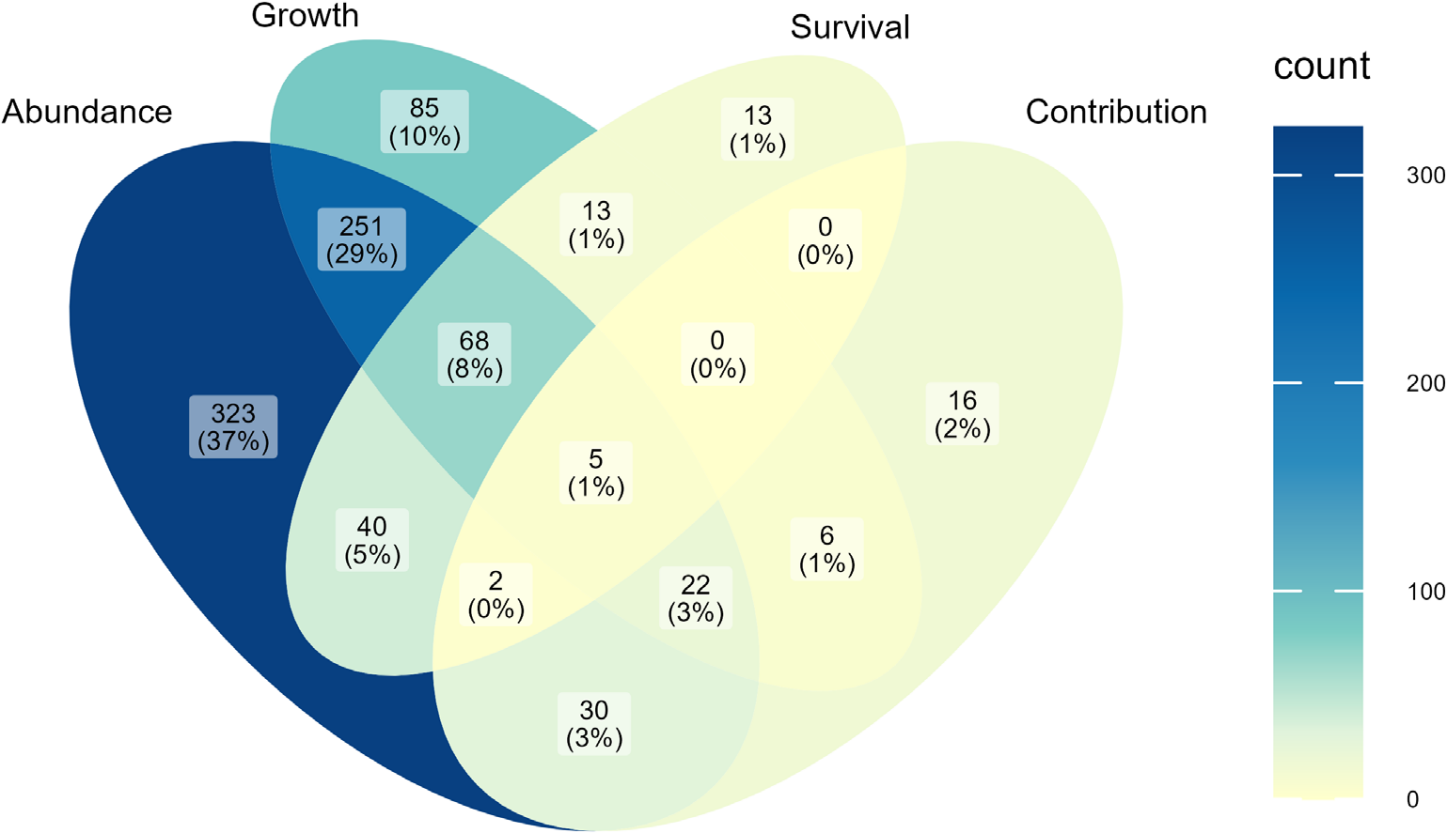
Venn diagram representing the combination of the four metrics from Beck *et al*. (2001) – abundance, growth, survival and juvenile–adult contribution – applied in papers published on juvenile habitat quality since 1970. Numbers represent the count of papers using a given combination of metrics, with these numbers expressed as a percentage of the total 874 studies given in parentheses.

The number of studies measuring juvenile habitat quality remains dominated by the abundance metric. Abundance was measured in 741 (85%) of the 874 studies, followed by growth (51%), survival (16%) and juvenile–adult contribution (9%; percentages sum to more than 100% as more than one metric could be measured in a single paper). The number of studies on abundance (Fig. 1A) and growth (Fig. 1B) has generally increased, whereas the number measuring survival (Fig. 1C) peaked between 2005 and 2010 and has since declined. Measurement of juvenile–adult contribution started more recently, in 1994 (Carr, 1994), and has increased slowly since then (Fig. 1D). Most studies (50%) used only one metric, with increased rarity of studies combining more metrics (Figs 1E and 2). Abundance–growth combinations were most common (29%), followed by abundance–growth–survival (8%) and abundance–survival (5%; Fig. 2). Our systematic review is therefore consistent with Lefcheck *et al*. (2019) in finding limited adoption of two key recommendations by Beck *et al*. (2001), that: (*i*) juvenile–adult contribution is the gold standard; and (*ii*) combination of multiple metrics is necessary to measure juvenile habitat quality effectively.

The different metrics of juvenile habitat quality are associated with contrasting challenges and opportunities (Table 1). Imbalance in the application of metrics likely reflects convenience and feasibility rather than convergence on an optimal approach. Given the challenges in measuring juvenile–adult contribution directly, assessment of proxies is necessary. However, the interpretation of these proxies to infer juvenile habitat quality may not follow expectations (Box 1). Identifying an appropriate metric requires an understanding of the species and seascape, as well as calibration against direct measures of juvenile–adult contribution.

**Table 1:** Opportunities and challenges presented by each of the four metrics of juvenile habitat quality.

	Opportunities	Challenges
Abundance	Routinely and widely measured using readily available and well‐established methodology. Broad range of methods available that can be employed at different spatial and temporal scales. Often the only option for long time series and therefore information at the broadest spatial and temporal extent.	There can be substantial modification by stochastic environmental influences in earlier juvenile stages: responds dynamically to complex interactions of settler supply with growth and survival after settlement, both of which can vary in response to habitat characteristics and environmental variables. Challenging to compare among certain combinations of habitat types, due to differing suitability and efficiency of methods. Provides no information about functional role of habitat in supporting growth and survival. Eulerian methods provide a snapshot for a limited number of locations and times: abundances can be very dynamic. Lagrangian methods can provide good spatiotemporal coverage, but in a limited number of individuals.
Growth	Complements abundance‐only methods by informing on the functional importance of different habitat types. Advantages when comparing different habitat types because is less influenced than abundance by habitat‐related differences in capture efficiency. Range of methods available that can be employed at different spatiotemporal scales. Measurements obtained readily alongside abundance surveys.	Method selection often based on feasibility rather than informing on growth at suitable spatial and temporal scales. Only provides an indirect assessment of survival. Growth is an integrative process and may be difficult to link with specific habitats (e.g. mobile species).
Survival	More direct metric of the functional importance of habitats than growth.	Little methodology available to measure survival: two main methods with major assumptions, limitations and potential for systematic biases among habitat types. High precision required to estimate differences due to extremely high proportional loss.
Juvenile–adult contribution	The most direct measure of juvenile habitat quality.	Requires considerable investment, infrastructure and expertise. Juvenile–adult survival rates can be very low, so variation occurs across a narrow range of values. Requires data on at least two life stages, across separate habitats, but patterns of habitat use can be complex. Sampling and data for both juveniles and adults must be compatible. Challenging to distinguish juvenile origins: need to find consistent, reliable, unique, archival identifiers of different habitats. Quantifiable at relatively coarse scales. Does not provide information on the specific mechanisms that control variation in contribution rates.

Box 1 **How can we interpret metrics of juvenile habitat quality?**

**Juvenile habitat quality can be inferred from the contribution a habitat makes to the adult population. While abundance, growth and survival may provide convenient proxies for this contribution, interpretation is not always straightforward.**

EXAMPLE 1: MORE FISH = BETTER HABITAT? Habitats containing more fish are not necessarily better quality. Abundances interact with individual growth and/or survival through density‐dependent processes (Holbrook & Schmitt, 2002). These interactions vary in strength and direction depending on environmental conditions and life‐history stage (Brown, Kokkalis & Stottrup, 2019; Ciotti *et al*., 2013b; Le Pape *et al*., 2003c; Rose *et al*., 2001).

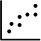
 **Positive association:** habitats with high abundance produce more adults because density‐dependent growth and mortality is relatively weak or juveniles select better quality habitats.

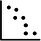
 **Negative association:** habitats with high abundance produce fewer adults due to high mortality and/or lower growth in crowded habitats (compensatory density dependence).

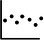
 **No association:** habitat‐specific variation in rates of growth and survival after settlement completely overwhelm variation in larval supply.

EXAMPLE 2: FASTER GROWTH = BETTER HABITAT? Growth indicates the contribution a habitat will make to adult populations through its interaction with juvenile survival. Depending on the context, however, the strength and direction of this interaction can vary substantially. Life‐history trade‐offs influencing the survival benefits of different growth trajectories are critical (Able, 1999): habitat quality may relate more to the ratio between rates of growth and mortality (Dahlgren & Eggleston, 2000; Ryer & Hurst, 2008) than to either metric in isolation.

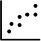
 **Positive association:** growth determines attributes (e.g. size, energy status) that enhance survival directly by conferring resistance to starvation, predators or other agents of mortality (Le Pape & Bonhommeau, 2015; Miller *et al*., 1988; Sogard, 1997).

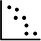
 **Negative association:** growth–survival trade‐offs mean that promotion of fast growth in certain individuals/environmental conditions takes place at the expense of survival (Biro *et al*., 2006; Mangel & Stamps, 2001; Robert *et al*., 2023; Ryer & Hurst, 2008).

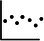
 **No association:** due to behavioural optimisation of habitat use under density dependence, growth rates are spatially uniform over the spatial scales that permit ideal free distribution (Ciotti *et al*., 2014).

EXAMPLE 3: GROWTH = GROWTH? A given metric is measured in different ways, with different interpretations. Growth, for example, is measured in a range of ‘currencies’ which may not covary (e.g. starving fish decrease in mass but not length). Differing measures may have contrasting implications for habitat quality, depending on the agent of mortality.

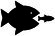
 **Agent of mortality = gape‐limited predators.** If predators are unable to consume juveniles above a certain size, survival can be related to growth in length, not mass or energy content (Cowan *et al*., 1996; van der Veer & Bergman, 1987).

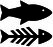
 **Agent of mortality = overwinter survival.** If fish with larger energy reserves resist seasonal food shortages better, survival can be related to energy storage (e.g. lipid content, condition factor) rather than growth in length (Hurst & Conover, 1998; Robert *et al*., 2023).

**→** **A solid biological understanding of the species and ecological system concerned is required in order to identify methods that offer clear, well‐constrained inferences on habitat quality.**

### (2) Measures for each metric

#### (a) Abundance

**Fig. 3:**
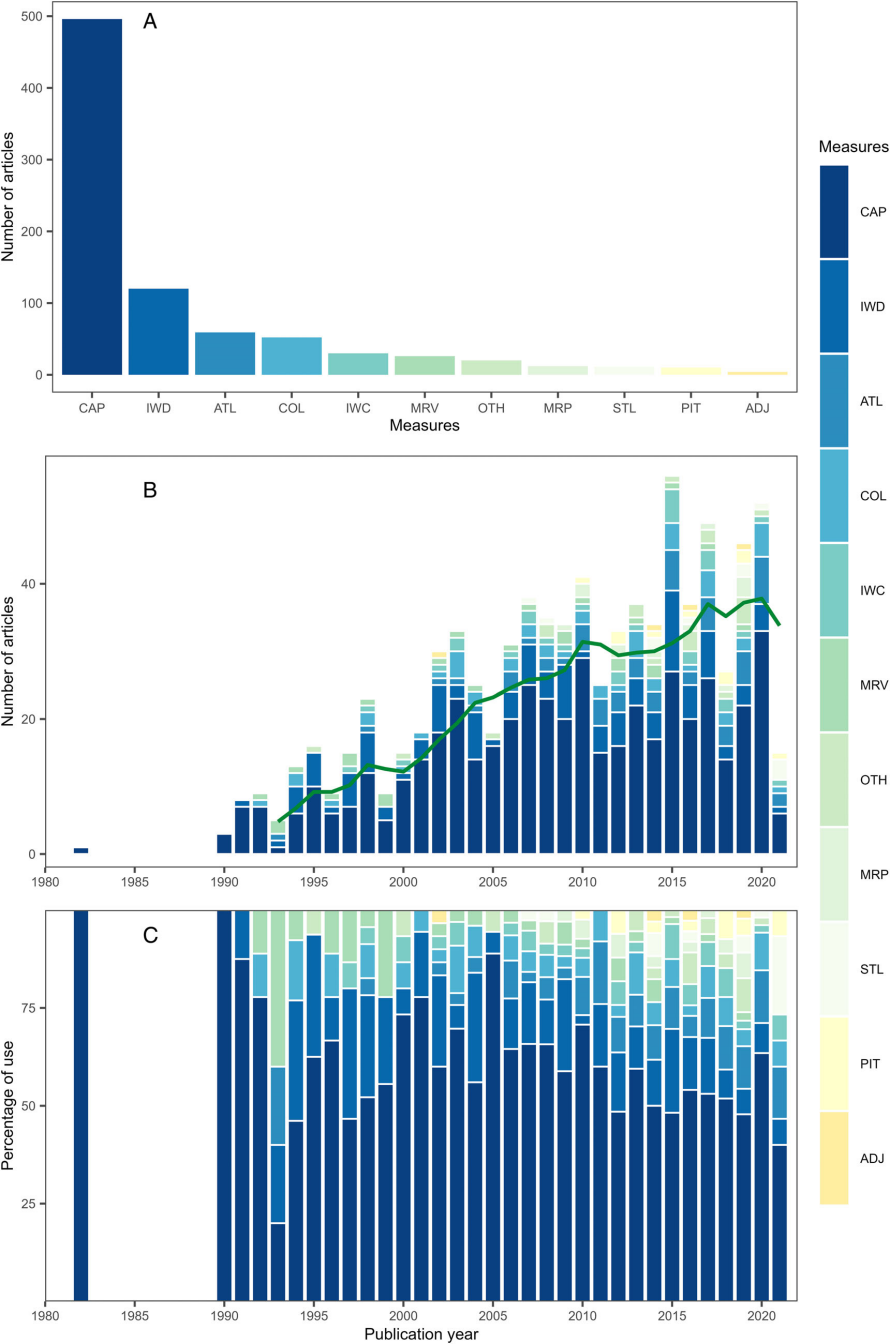
Abundance measures used to estimate juvenile habitat quality in papers published since 1970. (A) Sum of different measures used across all years. (B) Frequency of measures by year (green line represents the 5‐year simple moving average of the total number of papers published in a given year). (C) Relative frequency of measures by year. Note that 2021 is an incomplete year due to the timing of the literature search. Some papers employed more than one measure and are therefore counted multiple times. CAP, counting individuals captured in fishing gear; IWD, direct counts from in‐water observations; ATL, acoustic telemetry; COL, counting individuals captured using collector devices; IWC, in‐water counts from camera survey; MRV, mark–recapture using visual tags; OTH, other; MRP, mark–recapture using passive integrated transponder (PIT) tags; STL, satellite telemetry; PIT, tracking with PIT tags; ADJ, adult–juvenile linkage (e.g. larval supply).

Among the 11 categories considered (Table S1), two general approaches were used to infer juvenile habitat quality based on abundance. Most studies (644; 87% of the 741 studies measuring abundance) measured the juveniles present in different habitats (Eulerian approach), while 10% measured movements across habitats by tracking individuals (Lagrangian approach) and a further 3% measured both. Abundance was measured principally by capture sampling with fishing gear (67%; Fig. 3). Other common methods included direct visual observation (16%), acoustic telemetry (8%), collector devices (7%) and in‐water camera surveys (4%). The number of measures applied generally increased through time, with the use of passive integrated transponder (PIT)‐tagging and telemetry increasing since the early 2000s, yet remaining relatively rare (Fig. 3B, C).

The widespread reliance of abundance estimates on capture counts and direct observations relates to the convenience and reliability of these methods. Both can be employed with minimal fishing gear, at fine resolution, and provide a direct estimate of habitat use. Capture counts are the standard for long‐term monitoring surveys (e.g. annual scientific trawl surveys) and are therefore useful for understanding variation in juvenile abundance over large spatial and temporal scales. But these common methods also have limitations. Capture counts cannot easily be compared among habitats because fishing gear is often specialised to certain habitat types (e.g. unstructured soft bottom). Options for hard‐bottom and highly structured habitats are more limited, instead requiring in‐water direct or camera surveys. Even where the same fishing gear can be used in multiple habitats, capture efficiency may vary, creating systematic estimation biases (Riou, Le Pape & Rogers, 2001). Moreover, many methods (e.g. capture counts, visual observation and collector devices) can be labour intensive and only provide fragmented coverage. Each abundance sample represents a snapshot of the population; thus, many samples are required to identify trends amid patchy distributions and high sample variance. Sampling may also be biased by a focus on locations and times of day that are convenient to sample and life stages selected by specific gear or that are visible to observers. Therefore, the most common methods to measure abundance are associated with some limitations.

Lagrangian tracking of tagged juveniles provides information on distributions and therefore a complementary way of assessing abundance in different habitats. Acoustic telemetry is currently the dominant approach for tracking juveniles and can evaluate diverse research questions (e.g. Abecasis, Bentes & Erzini, 2009; Abecasis *et al*., 2013; D'Anna *et al*., 2011; Dudgeon *et al*., 2015; Knip, Heupel & Simpfendorfer, 2012; Mitsunaga, Endo & Babaran, 2013). Using a suitably designed acoustic telemetry array, tracked individuals can be localised to discrete habitats, with few sampling constraints (Thorstad *et al*., 2013). Tags can have relatively long lifespans, record numerous locations, and monitor habitat use continuously across ontogeny (Thorstad *et al*., 2013). Other electronic tag systems, including radio transmitters, data storage tags (DSTs, also termed archival tags), pop‐up satellite archival tags (PSATs) and PIT‐tags have become available (Thorstad *et al*., 2013). One limitation is that, despite progressive miniaturisation, many tags are still too large for smaller juveniles. While PIT‐tags can be inserted into smaller juveniles, these require close proximity between the tag and the receiver, and so are suited only to certain spatiotemporal scales and research contexts. Tags and receiver networks can be expensive to buy and maintain, and have trade‐offs between the spatial resolution of geolocation and spatial extent of the detection array. Finally, in comparison to Eulerian approaches the added resolution that Lagrangian approaches provide on habitat use of individuals is balanced against the more limited number of individuals or species tracked.

New technologies have become available to increase spatial and temporal coverage. These include underwater video, which can provide proxies for abundance such as MaxN: the maximum number of individuals visualised at any one time within a recording interval (Ellis & DeMartini, 1995). Underwater camera systems are now accepted for quantifying biodiversity and human impacts (Bicknell *et al*., 2016) and can be adapted to early life stages (Andradi‐Brown *et al*., 2016; Auster, Lindholm & Valentine, 2003; Piggott *et al*., 2020). They enable extensive spatio‐temporal data collection: fibreoptic‐cabled video observatories, for example, produce real‐time fishery‐independent stock assessment (Aguzzi *et al*., 2020; Ditria *et al*., 2022) and repeatable, permanently recorded sampling across different habitats, scales and depths (Piggott *et al*., 2020). While image/video storage, transfer and processing can limit the amount of data collected, computer‐vision (e.g. Artificial Intelligence, AI) can remove such bottlenecks (Chen *et al*., 2015; Sheaves *et al*., 2020). Limitations of video surveys include unquantified biases related to movement behaviour, artificial responses of fish to cameras or bait, and low water visibility. New acoustic camera sensors, such as Dual‐Frequency Identification Sonar (DIDSON) and Adaptive Resolution Imaging Sonar (ARIS), are unaffected by water clarity but currently lack the resolution to identify smaller juveniles (Jones, Griffin & Unsworth, 2021). Although our review did not find any studies that used aerial imagery from drones and other aircraft, this could estimate abundance across various spatio‐temporal scales and habitat types (Fortuna *et al*., 2013; Harris *et al*., 2019; Jennings, 1985) along with information on associated habitat characteristics (Madin, Darling & Hardt, 2019; Ventura *et al*., 2016, 2023). However, aerial imagery methods will likely remain restricted to large juvenile species and shallow, clear waters.

Environmental DNA (eDNA) is a powerful, emerging method to monitor fish and invertebrate assemblages (Karlsson *et al*., 2022; Rey *et al*., 2023), yet we found no applications measuring juvenile abundance. The main barrier to using eDNA is that sequencing does not reveal the age of the animal, so the approach cannot separate juvenile habitat from areas used by other life stages.

In general, capture methods and direct observations will almost certainly remain the mainstay of abundance estimation for juveniles. The value of generating long time series will be realised by building on these established methods. However, it is critical to complement these with the further development and deployment of camera and telemetry technology that can provide detailed information on distributions, from complementary Eulerian and Lagrangian perspectives, across a broader range of spatio‐temporal scales and habitat types.

#### (b) Growth

**Fig. 4:**
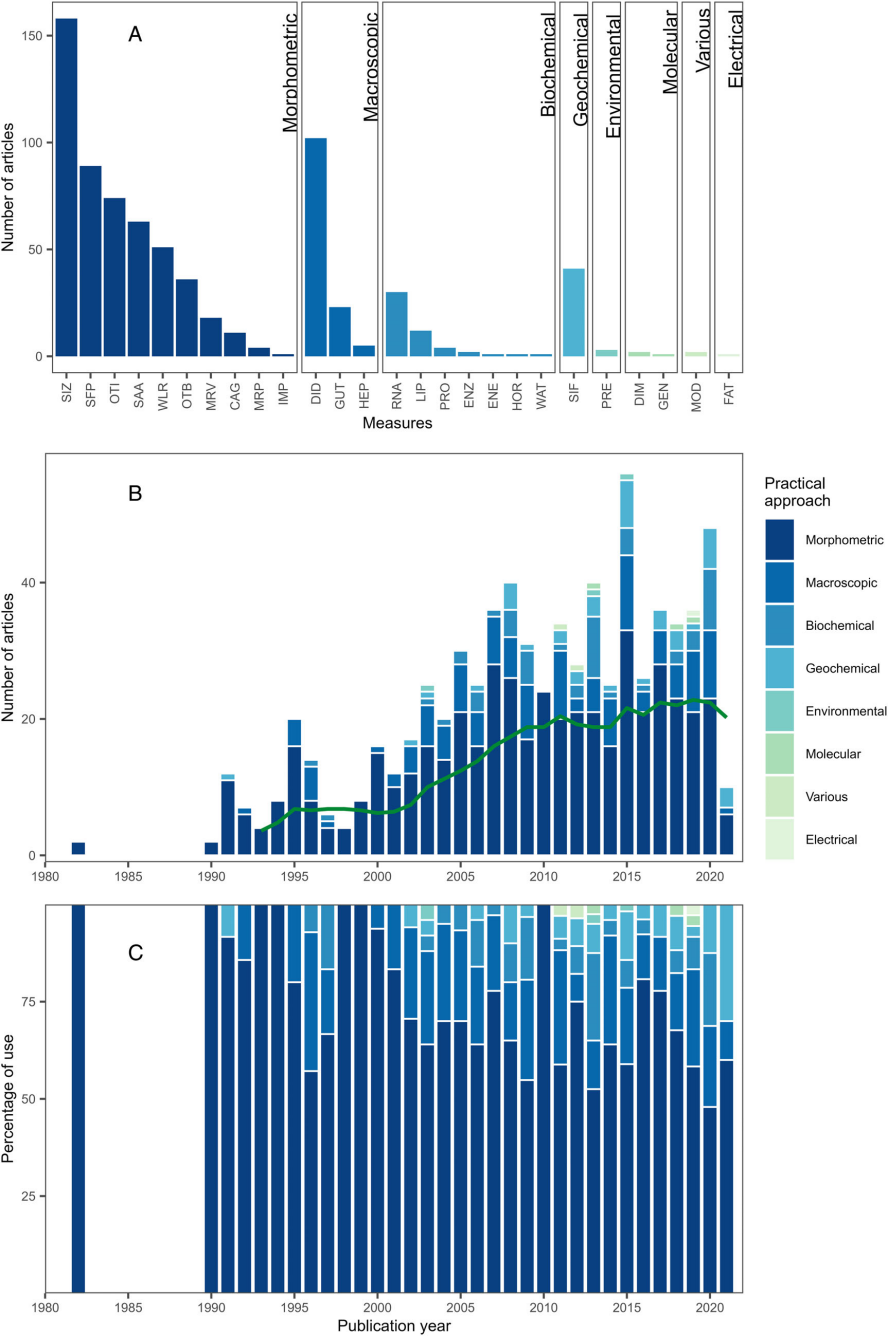
Growth measures used to estimate juvenile habitat quality in papers published since 1970. (A) Sum of different measures used across all years separated by practical approach. (B) Frequency of measures by year (green line represents the 5‐year simple moving average of the total number of papers published in a given year). (C) Relative frequency of measures by year. Note that 2021 is an incomplete year due to the timing of the literature search. Some papers employed more than one measure and are therefore counted multiple times. Measures were split into eight “practical approaches” describing the general family of methods used: “morphometric” (SIZ, measurements of size; SFP, size–frequency progression; OTI, increment widths of otoliths or other hard structures; SAA, size at age; WLR, weight‐to‐length ratio; OTB, size increments back‐calculated from otoliths or other hard structures; MRV, size increments from individuals marked with visual tags and recaptured; CAG, size increments from caged individuals; MRP, size increments from individuals marked with passive integrated transponder (PIT) tags and recaptured; IMP, intermoult period); “macroscopic” (DID, characterisation of diet from direct observation; GUT, gut fullness from direct observation; HEP, hepatosomatic index); “biochemical” [RNA, nucleic acid‐based indices (e.g. RNA:DNA); LIP, content of lipids extracted from tissues; PRO, content of protein extracted from tissues; ENZ, enzyme activity; ENE, energetic content of tissues; HOR, hormone level; WAT, water content of tissues]; “geochemical” (SIF, diet reconstruction from stable isotope fractionation); “environmental” (PRE, characteristics of prey in the environment); “molecular” (DIM, characterisation of diet from metabarcoding; GEN, gene expression); “various” (MOD, bioenergetics model based on field measurements); and “electronic” (FAT, lipid content measured using a fat meter).

Across the 450 studies using growth as a metric of juvenile habitat quality, 26 measures were applied (Table S2; Fig. 4A). Given this diversity, we pooled measures into six categories reflecting focal processes. These included, in order of decreasing application frequency, direct measures of growth and indirect proxies based on feeding, proportional growth, energy acquired, metabolic processes, and those using models to derive estimates from field measurements (“derived”; e.g. bioenergetics models). Eight main practical approaches were applied to quantify the focal processes: morphometric, macroscopic, biochemical, geochemical, environmental, molecular, various (combination of approaches) and electrical (Fig. 4A; Table S2).

The focal processes studied varied in frequency (Fig. 4A; Table S2). The most common focal process, direct growth (344 of 450 growth studies, 76%), used morphometric measurements of size, size–frequency progression, size‐at‐age and, to a lesser extent, paired morphometric measurements in individually tagged or caged individuals. The second focal process, feeding (38%), mainly used macroscopic examination of gut contents or geochemical analysis of tissues. Three studies used prey abundance, while two studies applied molecular methods (DNA barcoding) to characterise diet. The third focal process, proportional growth (24%), used structures (e.g. otoliths) assumed to scale isometrically with body size and measured growth by otolith increment width or progression in otolith back‐calculated length‐at‐age. The fourth focal process, energy acquired (17%), used either morphometric measurements (weight‐to‐length ratios), biochemical measurements (lipid, protein, energy, or water content of tissues), macroscopic examination of tissues (hepatosomatic index), or a fat meter. The fifth focal process, metabolic processes (8%), mainly used biochemical measures of nucleic acid indices, and sometimes other biochemical or molecular techniques such as enzyme activities, gene expression, or hormone levels. Finally, just two studies (0.5%) used field measurements to derive growth predictions based on bioenergetics models (Table S2).

Growth measures were combined with one another more frequently than for other metrics. Of 450 studies measuring growth, 59% used one measure, 26% combined two measures, and 15% combined three or more measures. Practical approaches diversified over time (Fig. 4B, C): morphometric and macroscopic approaches began increasing in *ca*. 1990, whereas the rise in biochemical, geochemical and molecular approaches started later (*ca*. 2000s), in line with their technical development and increased accessibility.

Widespread use of morphometric measures likely resulted more from availability and practicality, than from superior performance as an indicator of juvenile habitat quality. Direct measures of size are easy to obtain and were very common, but size confounds growth with age unless accompanied by aging, which can be more time‐consuming. Furthermore, size is an integrated, whole‐lifetime metric of growth, and even weight‐to‐length ratios (condition) can be relatively unresponsive to dynamic environmental variables (Ciotti, Targett & Burrows, 2013a). Measuring progression in size over a defined time window enables finer resolution estimates and avoids assumptions about birth dates, but this requires more intense sampling and remains relatively coarse resolution. Finally, growth estimates based on population averages of individual size are potentially confounded by processes other than growth, such as size‐selective mortality and migration, which influence size distributions (Bartolino *et al*., 2008; Ciotti *et al*., 2014).

Diet and feeding measures are straightforward, but indirect, proxies for growth and nutritional condition. Diet can be highly dynamic, indicating changes in feeding success at the scale of a few hours (Ansell & Gibson, 1990; Fonseca, Colclough & Hughes, 2011) and thus enabling energetic advantages of habitats to be identified with finer resolution than direct measures of growth and nutritional condition. By contrast, geochemical measures (e.g. stable isotope fractionation) reflect integrated feeding history (Poiesz *et al*., 2023). Diet‐based measures, however, measure consumption and not the energetic consequences directly. Both pre‐consumptive metabolic costs involved with feeding and post‐consumptive constraints of digesting certain prey items can uncouple food intake from the energetic outcome for the organism (Goodrich *et al*., 2022; Lankford & Targett, 1997).

Analysis of otoliths and other hard structures is widely used for measuring age and growth. While these do not directly measure whole‐organism growth, the size of hard structures usually scales proportionally with body size (Brown *et al*., 2019). Archival structures are available in preserved samples, which are accessible historically and retrospectively, and can chart age, growth and additional information such as physiological condition and habitat‐specific geochemical signatures (Avigliano & Volpedo, 2016; Reis‐Santos *et al*., 2022). Otolith increment widths are sensitive to changes in growth conditions, enabling fine‐resolution estimates (Fey, 2005; Peck *et al*., 2015). One drawback is that availability of archival structures is limited outside bony fishes and some molluscs. Otolith analysis can also be time‐consuming, and this may limit spatial and temporal coverage.

Application of indirect biochemical proxies of growth has increased recently but remains infrequent. Nucleic acid‐based indices (e.g. RNA:DNA) and lipid content analysis were the most common, with only four studies adopting other biochemical measures (Table S2). As sensitive, individual‐level, high‐throughput measures, nucleic acid‐based indices can reveal broad‐extent, fine‐resolution dynamics of juvenile growth (Ciotti *et al*., 2010, 2013c; Stierhoff, Targett & Power, 2009). The drawbacks are that these techniques can be expensive, require specific technical expertise and have special requirements for sample storage.

Despite the rise of molecular technologies across assorted biological disciplines, application to assessing juvenile habitat quality is rare, as this was documented in just one study of metabolic and growth processes and two diet studies. Such techniques have great potential to detect mechanisms and determinants of juvenile habitat quality. For example, measuring expression of specific genes underlying growth limitation can identify causes of growth variation (Liu *et al*., 2022). Similarly, metabolomic profiling offers a sensitive and detailed indicator of how different habitats affect pathways of energy production, growth, and immune responses (Goode, Dunphy & Parsons, 2020) while measurement of hormone concentrations can reveal consequences of environmental stress on growth (Perez‐Dominguez & Holt, 2006).

Across the wide range in use, the method to measure growth will have a critical bearing on inferences for habitat quality. Differing techniques reveal growth processes at vastly different scales, some of which can easily be incorporated into abundance monitoring programmes. Furthermore, growth is currently measured in a range of “currencies” (e.g. growth in length, mass or energy content), each with different ecological implications and therefore interpretations for habitat quality (Box 1). In many cases, however, growth estimates rely on measures that are readily accessible. Preferably, selection of approaches should be based on a solid understanding of the species and ecological system concerned to identify measures that will (*i*) respond at scales appropriate to relevant environmental drivers, and (*ii*) be relevant to specific mechanisms of mortality and thus habitat quality. Further development and application of the diverse measures available holds great promise to determine patterns and causes of variation in growth and energetic status in different habitats.

#### (c) Survival

**Fig. 5:**
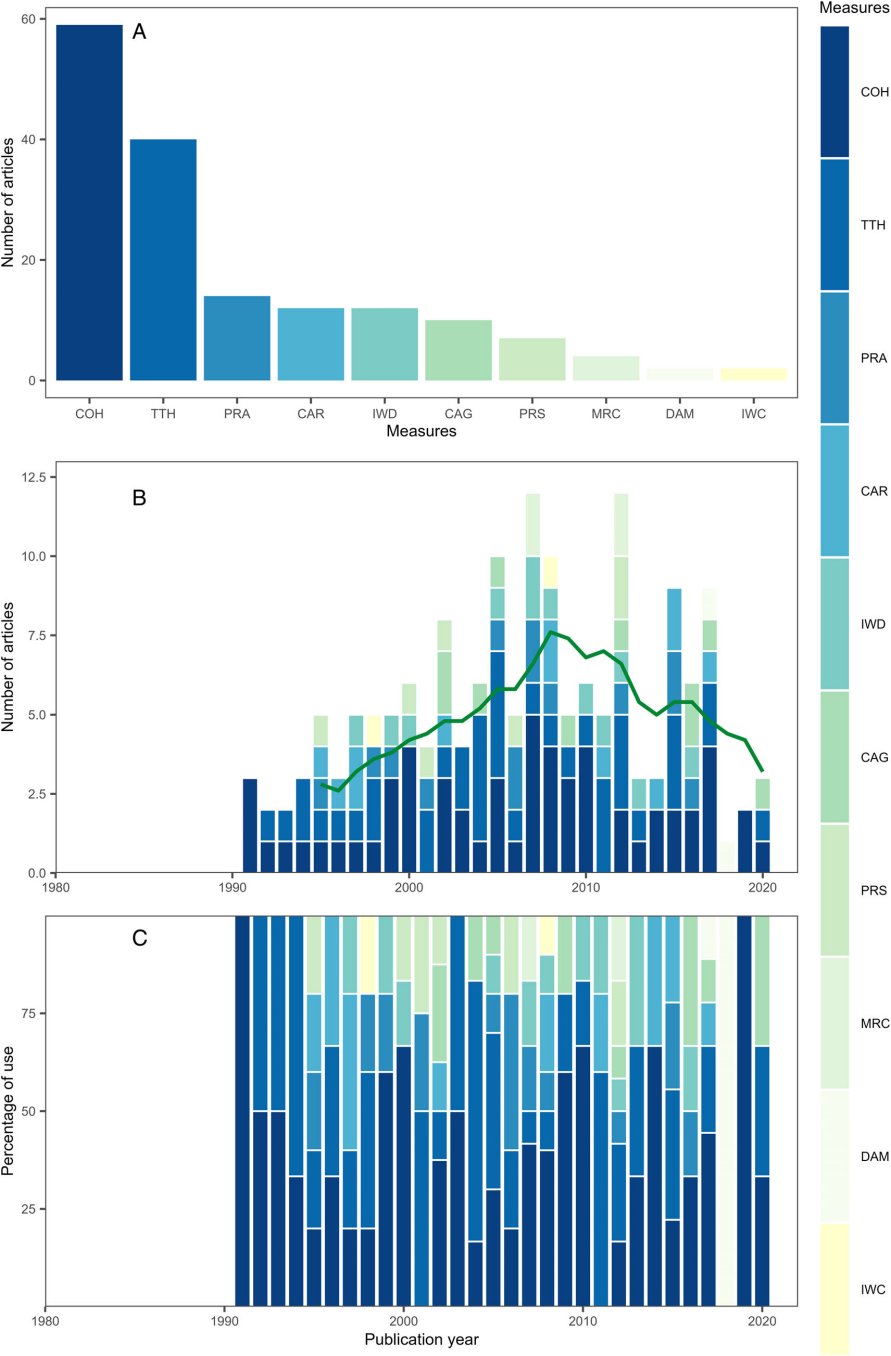
Survival measures used to estimate juvenile habitat quality in papers published since 1970. (A) Sum of different measures used across all years. (B) Frequency of measures by year (green line represents the 5‐year simple moving average of the total number of papers published in a given year). (C) Relative frequency of measures by year. Note that 2021 is an incomplete year due to the timing of the literature search. Some papers employed more than one measure and are therefore counted multiple times. COH, cohort analysis (rates of decline in abundance of cohorts); TTH, tethering (disappearance of tethered individuals *in situ*); PRA, predator abundance; CAR, carcasses remaining; IWD, direct observation of mortality events by in‐water observers; CAG, caging study (e.g. predator exclusion experiments); PRS, predator stomach contents; MRC, mark–recapture; DAM, damage accrued; IWC, observation of mortality events by in‐water cameras.

We found 10 measures applied across 141 studies using survival as a metric of juvenile habitat quality (Table S3; Fig. 5A). Most examined rates of decline in cohort abundance (42% of the 141 studies) or the disappearance of tethered individuals (28%). Between 5 and 10% of survival studies measured predator abundance, directly observed mortality events, counted carcasses, undertook caging experiments or examined predator stomach contents. The frequency of studies using survival as a metric increased until the mid‐2000s and declined thereafter (Fig. 5B, C), which may have been rooted in criticisms of tethering methodology (Baker & Sheaves, 2007; Halpin, 2000; Haywood *et al*., 2003; Peterson & Black, 1994; Zimmerfaust *et al*., 1994) and tightening of protocols for ethical use of animals in research. Most studies (88%) employed only a single survival measure, while 9% employed two and 3% employed three.

Shortcomings of tethering are well documented (e.g. Baker & Waltham, 2020; Peterson & Black, 1994). Biases include preferential selection of tethered prey by predators, increased susceptibility of prey to predators by reducing effectiveness of escape responses (Zimmerfaust *et al*., 1994) or enhanced detection or consumption probability (Barshaw & Able, 1990; Pursche, Suthers & Taylor, 2009). Tethering therefore amplifies mortality, not only from natural predators, but also from opportunistic predators to which a prey might normally be immune (Haywood *et al*., 2003). In field experiments, tethering can bias not only estimates of predation intensity within a single habitat but also between‐habitat comparisons, due to habitat‐specific artefacts of differing magnitudes (Micheli, 1996). Such biases can be mitigated through (*i*) mesocosm experiments to ensure that treatment‐specific biases are not present (Hovel & Lipcius, 2001; Pile *et al*., 1996), (*ii*) deployment of video cameras in parallel (Baker & Waltham, 2020) to cross check that predators match known predators on untethered prey, and (*iii*) validation against independent methods to measure survival. Finally, even if habitat‐specific bias is avoided, it remains a relative, rather than absolute, measure of survival that must be used carefully when quantifying its impact on population dynamics (i.e. juvenile–adult contribution), particularly at large spatial scales.

The other commonly used measure, cohort analysis, is useful to evaluate juvenile survival over large spatial and temporal scales. Cohort analysis is commonly conducted using long‐term survey or mark–recapture designs that estimate age‐ or size‐specific growth and mortality by tracking the abundance of year classes through time (e.g. Puckett & Eggleston, 2012). Surveys must be designed to ensure that size‐specific fishing gear and habitat‐specific selectivity do not bias abundance estimates at different life stages (e.g. Addison, Lawler & Nicholson, 2003; Fraser, Greenstreet & Piet, 2007). Such surveys are often unable to estimate survival of the earliest, and most vulnerable, juvenile stages, that select complex, shallow subtidal or intertidal habitats which are inaccessible to survey vessels (Ruiz, Hines & Posey, 1993). Pre‐ and post‐settlement abundance can be de‐coupled within hours of settlement, such that weekly or monthly surveys may miss the early juvenile stages and timing of natural mortality (Eggleston & Armstrong, 1995; Steele & Forrester, 2002). Furthermore, isolation of discrete cohorts is challenging (van Montfrans *et al*., 1995), as is separation of mortality from migration (Etherington, Eggleston & Stockhausen, 2003).

Few methods exist to measure survival and these are often applied at different spatiotemporal scales, which may underlie the rarity of the use of multiple measures in single studies. Given the numerous underlying assumptions, use of a single measure limits the usefulness of survival as a metric for habitat quality. Combining measures would allow cross validation of results and more robust inferences. For example, the combination of tag–recapture and telemetry is promising for disentangling natural *versus* fishing mortality (Bacheler *et al*., 2009a). When measures are applied at different spatial and temporal scales, inferences can be broadened. For example, small‐scale mark–recapture studies can ground‐truth estimates of growth and survival from large‐scale cohort analyses (e.g. Puckett & Eggleston, 2016). Moreover, monitoring programmes using acoustic telemetry arrays are increasingly providing long‐term, consistent, size‐specific abundance and demographic data from a range of locations (e.g. Goulette *et al*., 2014). Classic monitoring programmes, such as fishery‐independent trawl surveys, generate size‐specific abundance data for cohort analyses. Pairing survey‐generated cohort analyses with acoustic telemetry (e.g. Goulette *et al*., 2014) provides a powerful approach to estimate survival of free‐ranging marine species. Hence, the emerging choice for researchers is not to decide on a single measure, but rather which small‐scale measures pair with large‐scale survey data to facilitate robust inferences on survival.

Throughout the history of juvenile habitat research, therefore, two survival measures have dominated, yet these are both associated with important disadvantages. Beyond these two, few other methods are currently available, and new or developing technologies are not forthcoming. A key focus of future survival studies will be to combine the limited available approaches to provide more robust estimates at realistic spatiotemporal scales.

#### (d) Juvenile–adult contribution

**Fig. 6:**
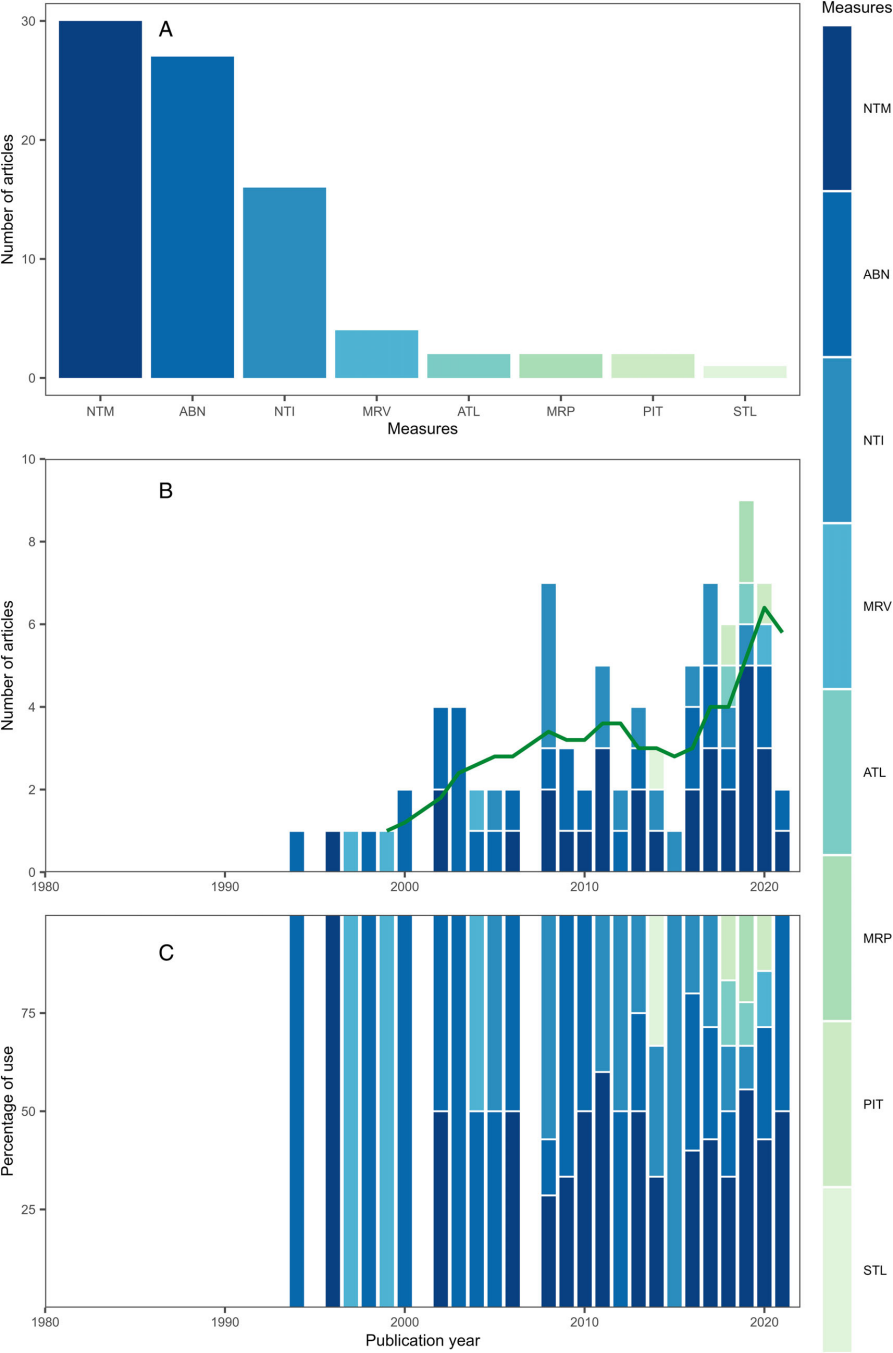
Measures of juvenile–adult contribution used to estimate juvenile habitat quality in papers published since 1970. (A) Sum of different measures used across all years. (B) Frequency of measures by year (green line represents the 5‐year simple moving average of the total number of papers published in a given year). (C) Relative frequency of measures by year. Note that 2021 is an incomplete year due to the timing of the literature search. Some papers employed more than one measure and are therefore counted multiple times. NTM, tissue microchemistry as natural tags to identify juvenile origins of the adult population; ABN, relationships between juvenile abundance measures from different habitats or between juvenile and subsequent adult habitats; NTI, tissue stable isotopes as natural tags to identify juvenile origins of the adult population; MRV, mark–recapture with visual tags; ATL, acoustic telemetry; MRP, mark–recapture with passive integrated transponder (PIT) tags; PIT, telemetry with PIT tags; STL, satellite telemetry.

Contribution of juvenile habitats to adult populations (juvenile–adult contribution) was the least reported metric of habitat quality, measured in 81 of 874 studies. Eight measures were used that can be grouped into three categories: abundance patterns, tracking natural tags and tracking artificial tags (Table S4; Fig. 6A). Measuring contribution rates using abundance patterns is based on the relationships between juvenile abundance measures from different habitats or between juvenile and subsequent adult habitats (e.g. Jones *et al*., 2010; Le Pape *et al*., 2003b; Wilson *et al*., 2016). Natural tags involve reconstructing movements based on signatures within archival tissues, such as stable isotopes in soft tissue (e.g. McMahon, Berumen & Thorrold, 2012; Niklitschek *et al*., 2014) or otoliths (e.g. Kimirei *et al*., 2013) and microchemistry of otoliths (e.g. Fodrie & Levin, 2008; Schilling *et al*., 2018; Tanner *et al*., 2013). Artificial tagging methods include acoustic telemetry (e.g. Hollensead *et al*., 2018), mark–recapture with PIT or visual tags (e.g. Wilson, Adams & Ahrens, 2019), tracking with PIT tags (e.g. Dufour, Cantou & Lecomte, 2009) and satellite telemetry (e.g. Vandeperre *et al*., 2014). Among 81 studies assessing juvenile–adult contribution, the most frequently used measures were based on natural tags (57%) and involved microchemistry (37%) or stable isotopes (20%), followed by abundance patterns (33%; Fig. 6A). The remaining measures, all involving artificial tags, were used in four studies (5%) or fewer (Fig. 6A). Studies measuring juvenile–adult contribution increased from the early 2000s, initially based on natural tags (microchemistry and isotopes) but diversifying from 2010 onwards with more emphasis on tracking methods involving satellite and acoustic telemetry, and mark–recapture with PIT and visual tags (Fig. 6B, C).

Abundance‐based analyses are readily accessible and based on easily available data from abundance surveys of juveniles and adults (e.g. Rooper, Boldt & Zimmermann, 2007). Studies collecting new data tended to be limited in temporal coverage (e.g. Bradbury *et al*., 2008; Dantas *et al*., 2012; de la Moriniere *et al*., 2002), whereas those using existing data (often for adults) can be limited if surveys are designed for purposes other than juvenile–adult contribution (e.g. Loneragan *et al*., 2013; Rooper *et al*., 2007). Juvenile–adult contribution and the role of different juvenile habitats based on abundance measures is indirect and often depends on the correlation between indices of abundance of juveniles and adults. Such analyses require major simplifying assumptions, including that (*i*) juveniles in a particular habitat migrate to the adult habitat, (*ii*) there is no immigration/emigration of juveniles and adults from/to other habitats, and (*iii*) higher correlation implies those habitats contribute more. These are strong assumptions that are difficult to confirm without additional data beyond measuring abundance; few studies evaluated these assumptions (but see Tobin *et al*., 2010). Abundance‐based analyses offer, at best and when assumptions are confirmed, first‐order (qualitative to semi‐quantitative) assessment of juvenile–adult contribution.

Newer approaches based on natural and artificial tags offer advantages over the abundance approach because they provide direct measures of juvenile–adult contribution (Calò *et al*., 2013). Natural tag approaches rely on a signal in juvenile tissues that is distinct among juvenile habitats and retained in adults, which allows the juvenile habitat of adults to be identified. Despite providing stronger evidence than abundance‐based correlations, these measures also make assumptions, particularly whether individuals are representative of the population, which requires an appropriate sampling design. Use of natural tags also presents logistical and sampling challenges. Costs and time required for analysis have historically limited sample sizes. Furthermore, some archival tissues such as otoliths are only available in actinopterygians, not chondrichthyans or invertebrates, although other tissues may provide promising options (Reis‐Santos *et al*., 2022; Taylor *et al*., 2017b). Data from natural tags can be highly variable at a range of spatial and temporal scales, which creates challenges when classifying chemical signatures in adults back to all potential juvenile habitat origins. For example, geochemical signatures vary among and within estuaries; unless juveniles in all possible habitats are sampled or simplifying assumptions can be made, the origin of adults will be uncertain. Further, geochemical signatures can differ among years, requiring cohorts to be matched to year‐specific juvenile baselines and potentially requiring multi‐annual sampling. Spatiotemporal complexity often produces relatively coarse geochemical signatures, which limits assignment of adults to juvenile habitats that cover broad regions. The need to enhance spatial and temporal sensitivity has, however, stimulated development of natural tag approaches that offer finer resolution estimates of juvenile–adult contribution and careful selection of chemical markers coupled with baseline sampling can produce tools to gain inferences across an increasingly wide range of scales (Reis‐Santos *et al*., 2022).

Tracking with artificial tags uses tag‐recaptures or detections to follow movement of juveniles within and between habitats, with contribution rates based either on returns as adults (e.g. Pickett, Kelley & Pawson, 2004; Saemundsson *et al*., 2020) or as late‐stage or emigrating juveniles immediately prior to joining the adult pool (e.g. Cianciotto *et al*., 2019; Wilson *et al*., 2019). A limiting issue is the low efficiency with which artificial tags follow juvenile to adult transitions due both to high juvenile mortality and to missed or unreported detections leading to small sample sizes. Acoustic telemetry can offer detailed information on multi‐habitat use of individual juveniles and across the juvenile–adult transition, but this requires careful design of acoustic receiver arrays at contrasting spatial scales (Burnsed *et al*., 2020). In addition, acoustic tags are large and can only be implanted in larger juveniles, although miniaturised technology is becoming available (Ivanova *et al*., 2020). While PIT tags are smaller, they require close‐proximity resampling and are thus best for species with small home ranges and restricted habitats (Cianciotto *et al*., 2019; Curtis *et al*., 2018; Wilson *et al*., 2019). Satellite tags have been used only in large, wide‐ranging species over broad spatial and temporal scales (Vandeperre *et al*., 2014). These limitations explain why the potential of electronic tagging has not been fully realised.

In summary, abundance‐based measures do not provide irrefutable evidence on juvenile–adult contribution, but the development of natural and artificial tags offers exciting potential. Presently, measures generate data on restricted and sometimes inappropriate spatial and temporal scales, which limits inference and applicability to management.

### (3) Taxonomic, habitat and geographic coverage

#### (a) Taxonomic

**Fig. 7:**
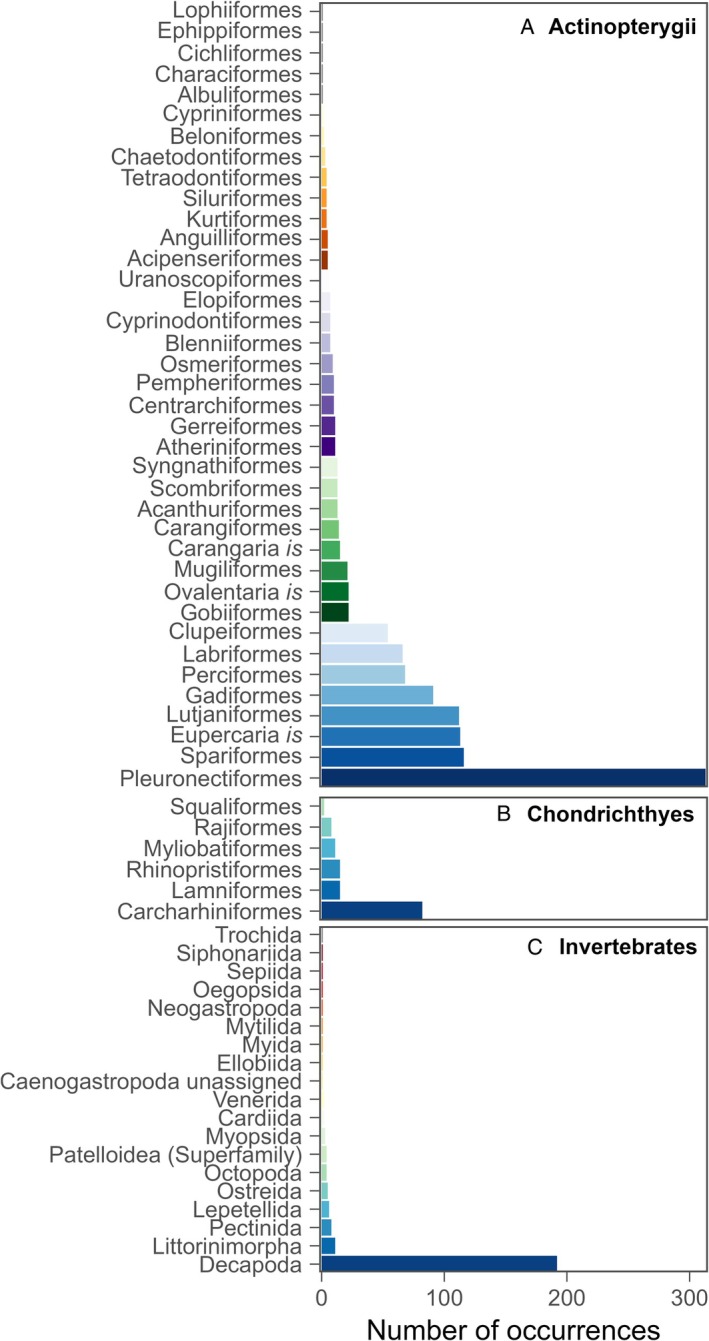
Taxonomic focus of studies of juvenile habitat quality in papers published since 1970. Bars represent the occurrences of species across all papers within each order in (A) class Actinopterygii, (B) class Chondrichthyes and (C) the combined invertebrate classes Malacostraca, Gastropoda, Bivalvia and Cephalopoda. Some papers study more than one species and are therefore counted multiple times, including for multiple species within the same order. Some orders that are currently of uncertain placement are succeeded by “*is*” for *incertae sedis*.

In our review, 874 studies measured juvenile habitat quality for marine or estuarine fish (excluding salmonids), crustaceans and molluscs. Studies often investigated multiple species, resulting in 1557 occurrences of paper–species combinations across 567 distinct species, 63 orders and six classes. By class, most occurrences focused on actinopterygians (76%), malacostracan crustaceans (12%) and chondrichthyans (9%), with few occurrences for gastropod (2%), bivalve (1%) or cephalopod (<1%) molluscs. By order, research focused disproportionately on flatfishes Pleuronectiformes (20%; Fig. 7A), even though this order only accounts for <3% of actinopterygian species (Nelson, Grande & Wilson, 2016). Other actinopterygian orders studied included Spariformes (7%), Eupercaria *is* (7%), Lutjaniformes (7%), Gadiformes (6%), Perciformes (4%), Labriformes (4%), Clupeiformes (3%) and a further 30 orders each with low representation (<1.5%; Fig. 7A). Chondrichthyan studies were dominated by ground sharks Carcharhiniformes (5%) with low representation (<1%) from five additional orders (Fig. 7B). Invertebrate studies were dominated by Decapoda (12%) with an additional 18 poorly represented (<1%) orders (Fig. 7C). The papers in our review therefore focused overwhelmingly on studies of flatfish and decapod crustaceans with much less attention to the wider diversity of other fish, crustacean and mollusc taxa. Possible reasons why these taxa were better represented is that they are of commercial importance in the areas where juvenile habitat research is concentrated (see Section II.3.*c* below), their juvenile habitats (soft sediment, shallow coastal and estuarine areas) are easily accessible and juveniles are amenable to capture and laboratory experimentation (Ciotti *et al*., 2014).

The variety of habitat quality measures was limited within each taxon. For example, almost half (44%) of the studies using counts of collector devices to measure abundance were for malacostracan crustaceans, 74% of acoustic telemetry involved chondrichthyans, and 87% of direct in‐water surveys focused on actinopterygians. Measures of survival were also taxon biased; tethering was most common in decapods (51% of occurrences) and cohort analysis in pleuronectiforms (53%). Some measures were excluded for major taxonomic groups; lack of otoliths or other archival structures in chondrichthyans and invertebrates restricted measurement of juvenile–adult contribution to only 13 occurrences outside the actinopterygians. In summary, methodological constraints restrict the range of approaches used within specific taxa.

#### (b) Habitat

**Fig. 8:**
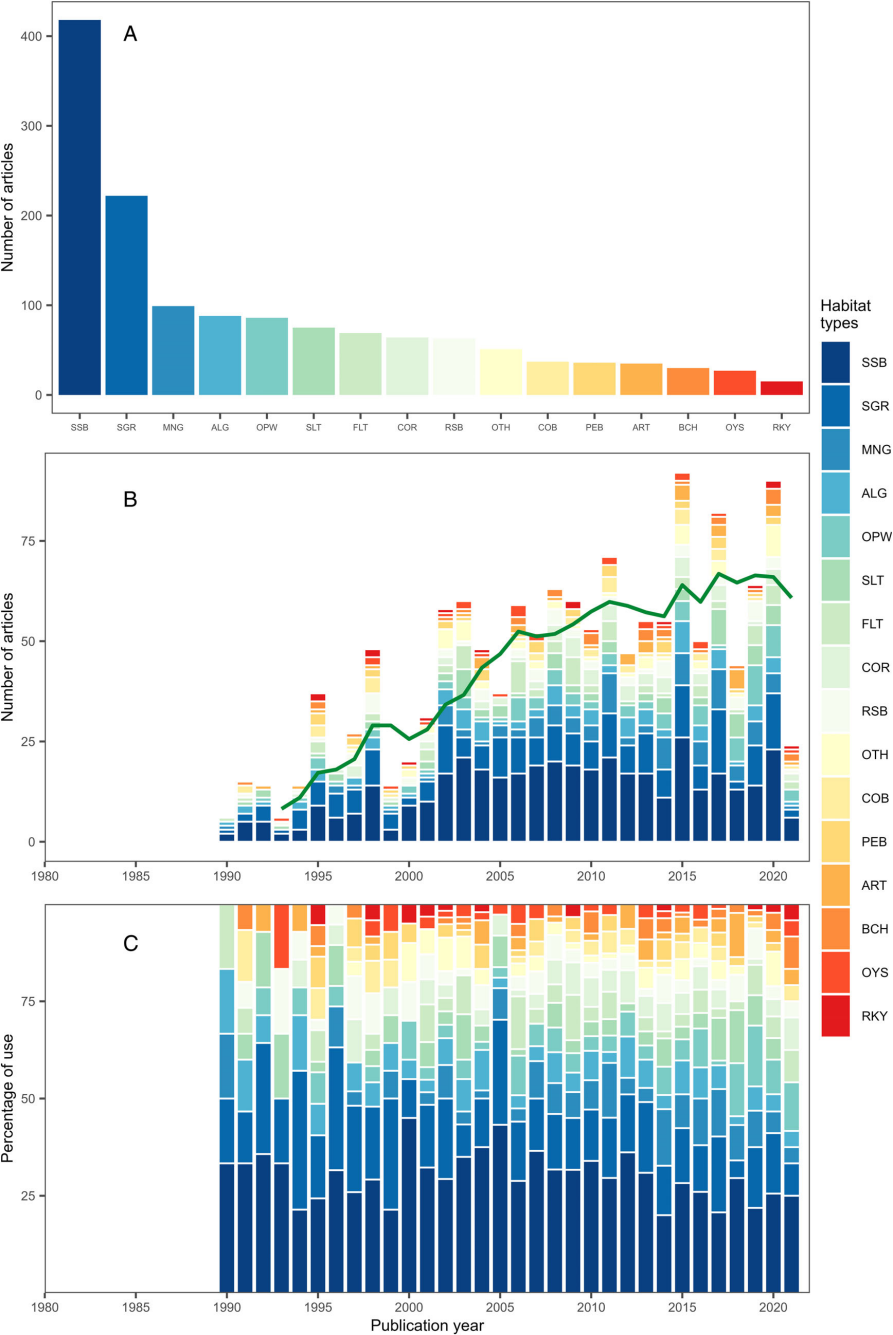
Habitat types investigated in papers studying juvenile habitat quality published since 1970. (A) Sum of studies investigating a particular habitat type across all papers. (B) Frequency of habitat types by year (green line represents the 5‐year simple moving average of the total number of papers published in a given year). (C) Relative frequency of habitat types by year. Some papers studied more than one habitat and are therefore counted multiple times. SSB, subtidal soft bottom; SGR, seagrass; MNG, mangrove; ALG, benthic macroalgae; OPW, open water; SLT, saltmarshes; FLT, intertidal flats; COR, coral reefs; RSB, rocky subtidal; OTH, other habitats accounting for <1% of studies; COB, cobble/boulder; PEB, pebble/gravel; ART, artificial substrates; BCH, sandy beaches; OYS, oyster beds; RKY, rocky intertidal. Other habitats (“OTH”), each accounting for <1% of studies, include worm reefs, mussel beds, other vascular plants, sponges, maerl, coastal wetlands, detritus and debris, cockle beds, other mollusc reefs, caves, drifting macroalgae, crustose coralline algae, amphipod reefs, other epifaunal reefs, and trees/shrubs. Plot does not include papers where the habitat type was unclear.

The papers within our review measured juvenile habitat quality in 30 habitat types. Of the 874 studies reviewed, almost half (48%) included subtidal soft bottom while 25% included seagrass beds (Fig. 8A). Seven habitat types were the focus of 5–11% of studies: mangrove (11%), benthic macroalgae (10%), open water (10%), saltmarshes (9%), intertidal flats (8%), coral reefs (7%) and rocky subtidal (7%). A further six habitat types were the focus of 1–5% of studies, including cobble/boulder (4%), pebble/gravel (4%), artificial substrates (4%), sandy beaches (3%), oyster beds (3%) and rocky intertidal (2%). The remaining habitat types were the focus of fewer than 1% of studies each. The majority (56%) of studies focused on a single habitat type with progressively fewer studies comparing two (24%), three (10%), four (6%), five (3%), six (<1%), or seven (<1%). In 16% of studies, the habitat type was not stated or was not clear: this is expected because some studies focus on comparing habitat quality across gradients of physiochemical conditions or among geographical locations rather than among discrete habitat types (e.g. Ciotti, Targett & Burrows, 2013b).

As for taxonomic coverage, representation of different habitat types varied among metrics and measures. Capture count dominated abundance measures in habitats suitable for active fishing gear, such as soft‐bottom subtidal and intertidal flats (>57% of studies). By contrast, in‐water direct observations were often used in areas unsuitable for fishing gear but with good water clarity, such as rocky intertidal (67%) and coral reefs (58%). The majority (53%) of satellite telemetry studies were conducted on large pelagic species (e.g. tuna and pelagic sharks) in open water. Following patterns of taxonomic diversity, survival studies were split between tethering of decapods in seagrass (30%) or cohort analysis of pleuronectiforms on subtidal soft bottom (36%).

Hence, the range of approaches applied to measure juvenile habitat quality is restricted within habitat type. An associated challenge is in finding methods for robust comparisons among habitat types. For example, measures of abundance from cameras or direct observations could be standardised against capture counts in soft‐bottom habitats to develop efficient and widely applicable methods (Bicknell *et al*., 2016).

#### (c) Geographic

**Fig. 9:**
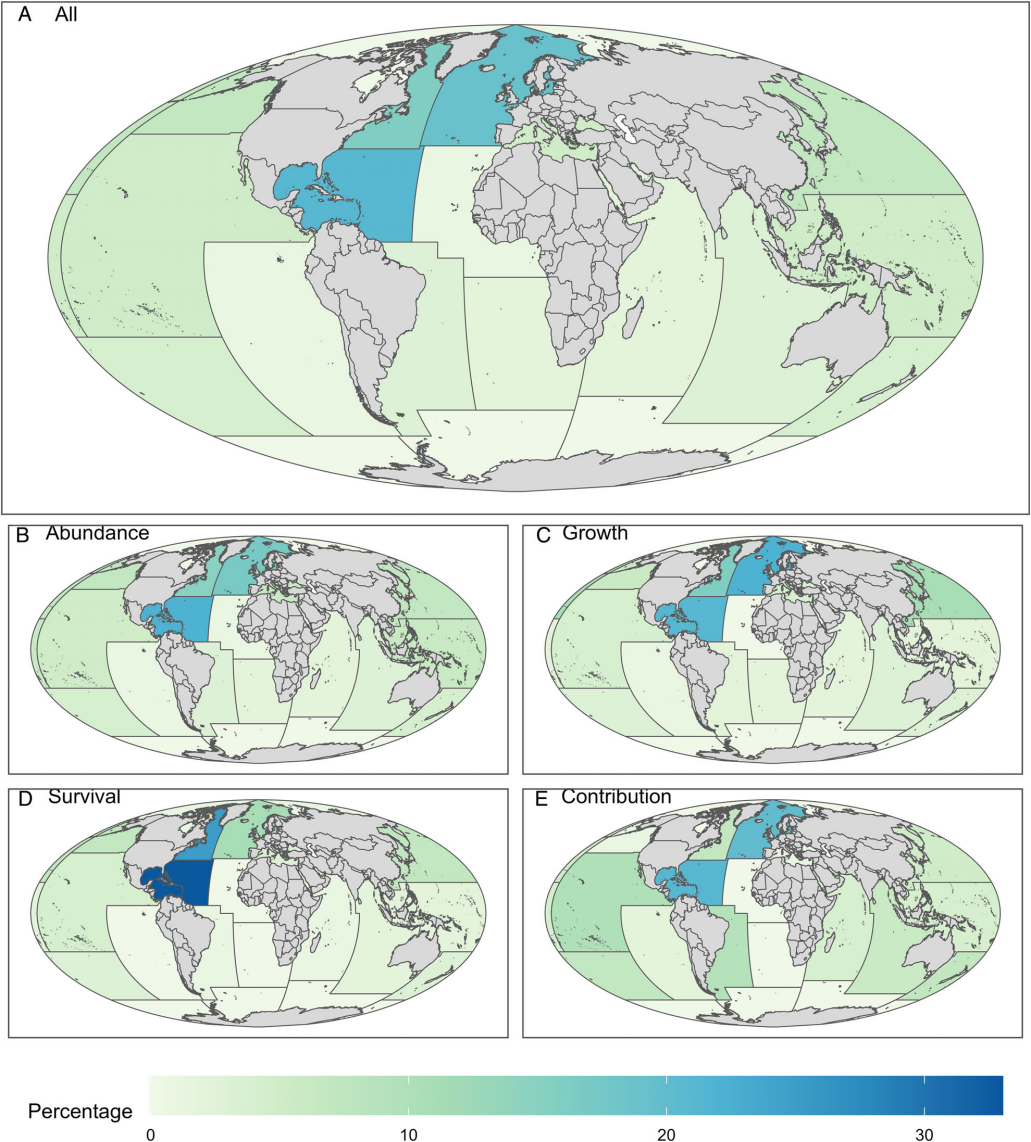
Geographic coverage across FAO regions of papers studying juvenile habitat quality published since 1970. Regions are coloured according to the proportion of total studies across all areas investigating (A) any metric of juvenile habitat quality, (B) abundance, (C) growth, (D) survival and (E) juvenile–adult contribution as metrics of juvenile habitat quality.

Studies of juvenile habitat quality covered a large geographical area, including all FAO fishing areas except the Southern Ocean (Atlantic, Indian Ocean and Pacific Antarctic Regions; Fig. 9A). Over half (56%) of the 874 studies were concentrated in Western Central (21%), Northeast (19%) and Northwest (16%) Atlantic. A further 23% were in the Northwest (7%), Northeast (7%), Western Central (5%) and Eastern Central (5%) Pacific. The Mediterranean and Black Sea contributed 5% of studies. Relatively few studies were in the Southwest Pacific (4%) or Southwest Atlantic (3%). No more than 25 studies (<3%) were in any of the other regions including the Eastern (3%) and Western (2%) Indian Ocean, the Southeast Atlantic (2%) and Pacific (<1%) Oceans, the Eastern Central Atlantic (<1%) or the Arctic Sea (<1%).

Application of certain metrics of habitat quality were even less evenly distributed than the studies overall (Fig. 9). Survival studies were concentrated in the Western Central and Northwest Atlantic, with less attention in the Northeast Atlantic (Fig. 9D). The Western Central and Northeast Atlantic were important for studies of juvenile–adult contribution, but the Northwest Atlantic and Northeast Pacific were less studied, with no juvenile–adult contribution or survival studies in the Arctic Sea, Eastern Central Atlantic or Southeast Atlantic (Fig. 9D, E).

Measures also differed among geographic regions. The 117 studies using visual census to assess abundance were highly represented in the Western Central Atlantic (27%) and the Mediterranean and Black Sea (15%). Of 30 studies adopting underwater video, most (30%) were in the Northeast Pacific. Almost half (49%) of the 37 mark–recapture studies for assessing abundance were in the Western Central Atlantic. For growth, 55% of the 29 studies using RNA‐based indices were in the Northeast Atlantic. Tethering was mainly conducted (77% of 39 studies) in the Northwest and Western Central Atlantic.

In summary, our knowledge of juvenile habitat quality is derived from restricted geographic areas. The limited diversity of measures being applied is exacerbated by geographic constraints and biases since many approaches are concentrated in specific regions.

### (4) Spatial and temporal scales of sampling

**Fig. 10:**
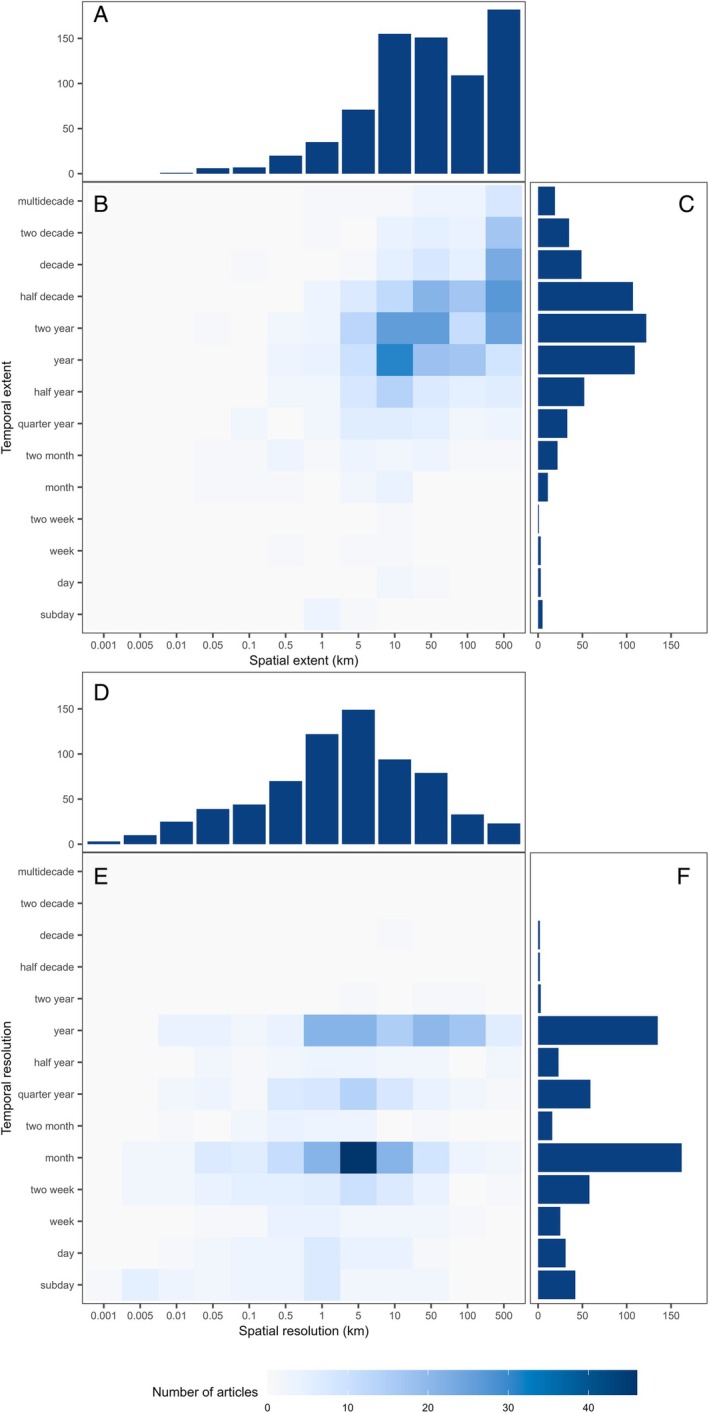
Combined heatmap and frequency bar charts of the spatial *versus* temporal scales of sampling for both the extent (A–C) and resolution (D–F) of papers studying juvenile habitat quality published since 1970. The frequency of spatial extents and resolutions (A and D, respectively) and the frequency of temporal extents and resolutions (C and F, respectively) include all records that address juvenile habitat in at least the spatial or temporal dimension. The frequencies of the combinations of spatial and temporal extents (B) and resolutions (E) share a common scale and contain only those studies addressing juvenile habitat in both dimensions, thus the numbers in heatmaps do not sum to the same as those in the frequency bar plots.

We recorded the spatiotemporal extent (duration) and resolution of studies. These represent the scales of sampling, but not necessarily the scales that characterise the processes being measured, which are determined by sensitivity of the measure and mobility of the species. The extent of studies was highly variable, spatially covering distances from 50 m to greater than 500 km and temporally covering durations from less than a day to multiple decades (Fig. 10). Studies were most commonly undertaken over extents of *ca*. 50 km (18–55 km) and over a two‐year period. While the temporal extent appears to be log‐normally distributed (Fig. 10C), the spatial extent is skewed toward larger scales (Fig. 10A). Spatial and temporal extent increased in parallel, with long‐term studies covering larger geographic areas (Fig. 10B).

**Fig. 11:**
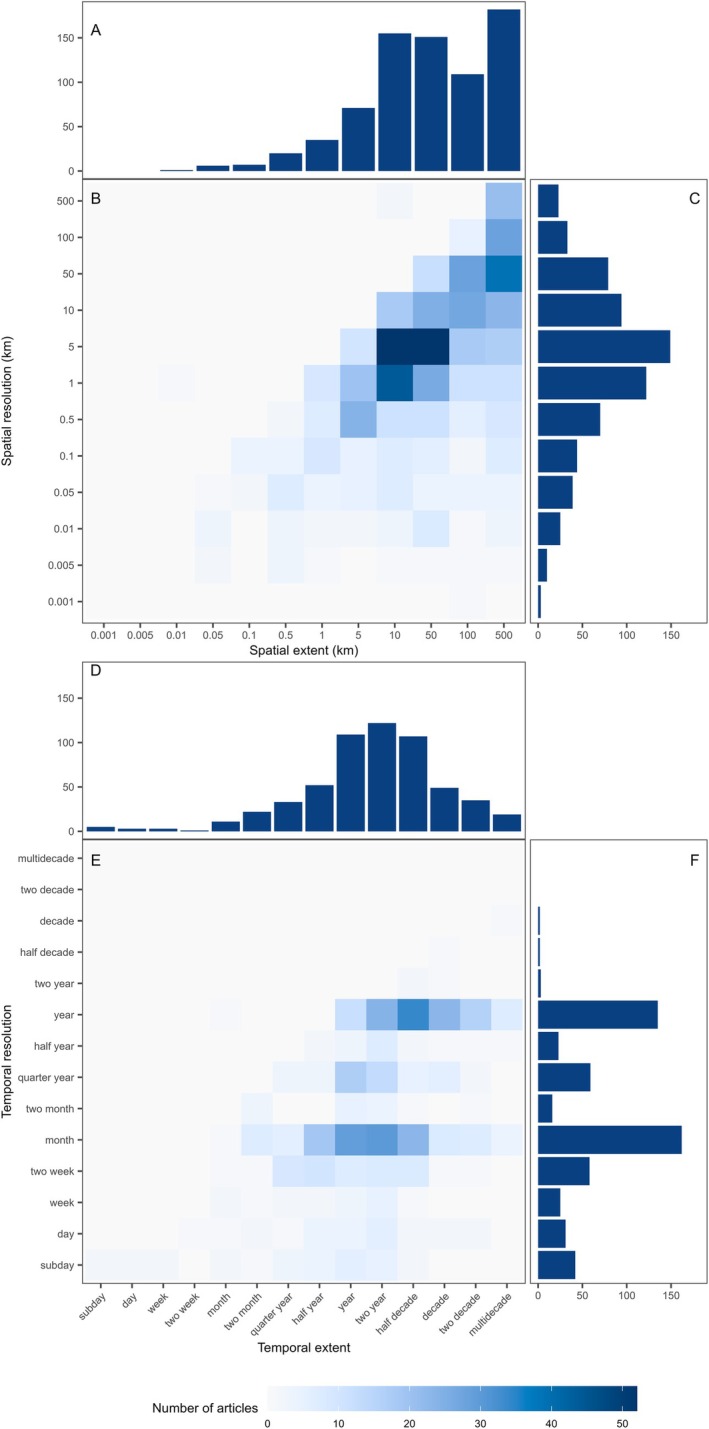
Combined heatmap and frequency bar charts of the extent and resolution of sampling for both spatial (A–C) and temporal (D–F) scales in papers studying juvenile habitat quality published since 1970. The frequency of spatial and temporal extents (A and D, respectively) and the frequency of spatial and temporal resolutions (C and F, respectively) include all records that address juvenile habitat in at least the spatial or temporal dimension, respectively. The frequencies of the combinations of spatial extents and resolutions (B) as well as temporal extents and resolutions (E) share a common scale and contain only those studies with information on both extent and resolution.

Spatial and temporal resolutions were equally variable, with high‐resolution studies sampling at 1 m and sub‐day scales. Spatial resolution increased proportionally with spatial extent (Fig. 11A–C), where the upper limit on resolution is, by definition, the extent and the lower limit trades off with extent due to resource constraints. Conversely, temporal resolution did not relate to temporal extent (Fig. 11D–F), where sampling designs led to higher rates of sampling across calendar months, quarter years (seasons), or calendar years than at other timescales. Some studies, however, utilised nested designs to compare processes at different scales (Able *et al*., 2012; Ciotti *et al*., 2013, 2013; Cuadros *et al*., 2017).

Patterns in scales of sampling suggest that resources impose restrictions on spatiotemporal coverage by (*i*) limiting ability to sample over large spatial and temporal extents, and (*ii*) reducing spatial and temporal resolution with increasing extent. Most studies were restricted to 1–3 years, which limits continuity and the ability to disentangle interannual and decadal level variability. Only where a research group secured consecutive projects (Archambault *et al*., 2018) or had access to long‐term monitoring programmes (van der Veer *et al*., 2022), could studies extend beyond a few years. Only 12% of studies utilised independent monitoring programmes (extensive/long‐term sampling efforts not conducted specifically for the purpose of addressing the study objectives), and even investigations of juvenile habitat at large extents were based on targeted research surveys (Ralph *et al*., 2013). This probably stems from the offshore focus of many monitoring programmes, which are aimed at adult stock assessment rather than inshore areas used by juveniles (Le Pape *et al*., 2020).

In summary, studies of juvenile habitat quality have sampled over a wide range of spatial and temporal scales. Studies with fine resolution and broad extent are relatively rare yet essential for understanding population‐level influences and fine‐scale drivers of variation in juvenile habitat quality.

### (5) Integration

By reviewing methods to measure juvenile habitat quality, we focused only on the start of the process to identify and protect habitats: integrating knowledge from different studies to evaluate juvenile habitat quality is equally critical (e.g. Heck, Hays & Orth, 2003; Lefcheck *et al*., 2019). One approach to combine metrics, used in 19 studies, was to calculate production, the rate of change in biomass or abundance per unit of habitat area. Definitions and units varied among studies but approaches fell into two categories: (*i*) absolute production indicating the total production of the study area [10 papers (e.g. Faunce & Serafy, 2008; Hughes *et al*., 2015)] and (*ii*) relative production, where production is compared among habitats [nine papers (e.g. Rudershausen & Buckel, 2020; Yeager, Acevedo & Layman, 2012)]. Two studies measured only abundance and converted that to areal production based on area size, but most studies calculating production reported two (mainly abundance and growth; seven studies), three (nine studies), or all four metrics of juvenile habitat quality (one study; Mace & Rozas, 2017). Not all the metrics reported in these papers were necessarily used in the calculation of production (e.g. Mace & Rozas, 2017).

Modelling methods represent an additional, crucial step for synthesising, generalising and building robust advice to inform management. Mathematical modelling is valuable for synthesising metrics of juvenile habitat quality and extending inferences to the population level (Lipcius *et al*., 2019, 2021) and across multiple spatiotemporal scales (Calò *et al*., 2013; Colloca *et al*., 2009; Nikolic *et al*., 2020). For instance, Hayes *et al*. (1996) used a stock‐recruitment framework to consider influences of variation in different components of habitat on population dynamics. Modelling approaches for integration of habitat‐quality data were not included our current review, but have been evaluated previously (Lipcius *et al*., 2019).

### (6) Summary and caveats

Our review focused on four “metrics” of juvenile habitat quality (i.e. abundance, growth, survival and juvenile–adult contribution) that are estimated using different “measures” or methods. Based on our review, we summarise the key opportunities and challenges associated with each metric (Table 1). Current applications of methods necessarily reflect feasibility and practicality considerations, rather than selection purely for their capacity to achieve relevant and robust measurements of juvenile habitat quality.

Our review indicated that measurements of survival and juvenile–adult contribution are employed much less frequently than abundance and growth, partly because they are more challenging to achieve and because there are fewer methodological options. We also found that combination of multiple metrics and measures in the same study is relatively uncommon, despite complementation between approaches offering a means to reduce uncertainty associated with any single approach. Limits to the range and combination of approaches used is exacerbated by the fact that metrics and measures tend to be concentrated in particular taxa, habitats or geographical locations (e.g. survival studies were concentrated in the Western Central and Northwest Atlantic). The unbalanced coverage of studies and restricted combination of approaches used limits comparative studies of juvenile habitat quality (e.g. among estuaries, between habitat types from different studies) because results among studies become confounded with method. Finally, our review confirmed that while sampling is conducted at a range of spatial and temporal scales, it is concentrated at intermediate extent and resolution. Our general understanding of juvenile habitat quality is therefore being built from many single‐metric (often abundance‐based) studies conducted on a limited diversity of species, habitats, and geographical regions and at a restricted range of spatiotemporal scales.

There are several important caveats to our review and the emergent results and recommendations. First, while we designed our methodology to be as comprehensive as possible, we recognise that a review of such a broad and diffuse topic as field measurement of juvenile habitat quality will partially be a product of the search and exclusion criteria applied. One challenge is in finding the right balance between including as many studies as possible without introducing bias by extending the list of search terms to those returning a narrow, proscribed set of taxa, habitats or locations. Highly precise search terms may add to the pool of papers included but create a bias in favour of certain types of studies. While we consider our review to be systematic, comprehensive and among the largest collations of field‐based juvenile habitat studies to date, our findings must nonetheless be interpreted in the context of the specific search and exclusion methodology we applied.

A second major caveat is that our review was restricted to empirical studies that used at least one of four metrics of habitat quality based on Beck *et al*. (2001). This precluded other data sources such as fluctuations in adult stocks following changes in juvenile habitat area (Heck *et al*., 2003), measurements of environmental hazards such as pollutants (Vasconcelos *et al*., 2011b) and local ecological knowledge on habitat quality from fishers and other communities (Kritzer *et al*., 2016; Ponton‐Cevallos *et al*., 2022; Serra‐Pereira *et al*., 2014). We also omitted studies whose primary focus was on ecological and habitat modelling (see Lipcius *et al*., 2019). These models often use the field data we discussed in this review, offer an approach for generalising field data‐based results (Gernez *et al*., 2023; Lefebvre du Prey *et al*., 2023) and can play a role in quantifying juvenile–adult contribution (Champagnat, Rivot & Le Pape, 2024).

Finally, we viewed each study as an independent unit and we did not factor into our analysis the specific aims of each study. Thus, we did not use emergent knowledge derived from interlinked studies of juvenile habitat published separately. By considering articles in isolation, we may miss insights from syntheses across related articles (e.g. multiple articles from a research programme or on a specific species). Within our primary focus on literature measuring juvenile habitat quality (Appendix S1), studies covered a wide range of specific aims and objectives, and these would have greatly influenced the methods selected.

## III. RECOMMENDATIONS

In this section, we make five recommendations for future field work, analyses and research. These recommendations emerged from our analysis of the reviewed studies and the opportunities and challenges listed in Table 1. They should be viewed in the context of the caveats resulting from limitations in the scope and approach of the review (Section II.6).

### (1) Look beyond abundance and use metrics related to habitat functioning

We recommend increased use of metrics of growth, and especially survival and juvenile–adult contribution, to provide critical information on habitat functioning. Abundance was the dominant metric measured and is likely to remain so because it is direct, and relatively easy/inexpensive to measure. Measuring patterns of abundance and habitat occupancy is an important first step in identifying habitats used by juveniles (Beck *et al*., 2001; Ford *et al*., 2010; Murase *et al*., 2020; Nagelkerken *et al*., 2015) and, due to frequent measurement, abundances are often the only long‐term data available. Limitations in using abundance to make inferences about habitat quality (Box 1), however, mean that inclusion of other metrics is a high priority. Growth and survival are rates that underpin how abundance translates to recruitment, and juvenile–adult contribution measures this recruitment enhancement directly.

The shift to, or inclusion of, rate‐based metrics will not only provide a more complete understanding of the full range of processes that determine juvenile habitat quality, it will also provide process‐based information on habitat functioning and enable easy integration with other studies of life processes and vital rates. Rates provide higher transferability and generalisation than abundance, increasing our ability to assess habitat quality and compare between habitat types and across seasons and years (Able, 1999). Our systematic review highlighted the various approaches available to measure growth, survival and juvenile–adult contribution at different spatial and temporal scales, including methods that can be applied as part of routine monitoring.

Exploring different metrics of juvenile habitat quality at early stages of a research programme can increase the efficiency and effectiveness of subsequent effort. The interpretation of metrics such as abundance, growth and survival in terms of juvenile habitat quality varies among species and study systems (Box 1; Searcy, Eggleston & Hare, 2007), but these may be less resource and time intensive to measure than juvenile–adult contribution directly. If it can be established that one of these metrics (or a combination, see Section III.3) accurately predicts juvenile–adult contribution, it may be used with increased confidence as a convenient proxy for juvenile habitat quality. Fodrie & Levin (2008), for example, established in California halibut *Paralichthys californicus* that expected contributions from juvenile habitats to subadult populations based on abundance metrics closely matched realised contributions inferred from natural tags. While such information provides a basis for using abundance as a metric of juvenile habitat quality in this particular instance, the same is not necessarily true for other species and study systems: Chittaro, Finley & Levin (2009) found that contribution rates from juvenile habitats to subadult populations of English sole *Parophrys vetulus* were unrelated to density or habitat area, while in a multispecies assessment the existence of this relationship varied among species (Vasconcelos *et al*., 2011a).

### (2) Design studies to be interpretable at different spatial and temporal scales

Studies should include sampling and experimental design considerations that enable the investigators, or others in subsequent analyses, to assess habitat quality at multiple spatial and temporal scales. A spatiotemporally explicit understanding of variation in juvenile habitat quality is necessary to capture both the fine scales at which individuals interact with habitats and the broad areas and longer periods of time over which these processes influence population dynamics. The ability to draw inferences at different spatial scales is important not only to build fundamental knowledge about the function of juvenile habitat (Nagelkerken *et al*., 2015; Sheaves *et al*., 2015), but also to meet a range of evidence needs in management and policy. For example, variation in habitat quality over the long term and at regional scales may be an important consideration in stock assessment or in determining the stability and persistence of habitats for conservation [e.g. informing on Marine Protected Area (MPA) design (Friedland, Ahrenholz & Guthrie, 1996)]. Meanwhile, a fine‐resolution understanding is necessary to evaluate localised impacts or mitigation measures (e.g. habitat restoration projects; Gernez *et al*., 2023). Careful integration of data collected across contrasting scales can reveal both fine‐scale mechanisms and population‐level consequences of variation in habitat quality (e.g. the cumulative works contributing to Archambault *et al*., 2018).

Although studies are not always designed to obtain fine‐grained and/or broad‐extent information, approaches are available to obtain measurements at these scales for most of the metrics of juvenile habitat quality (Section II.2). Juvenile–adult contribution, however, is a notable exception; in this case, refining natural and artificial tagging methods to achieve finer resolution estimates is a priority for method development (Section III.4). A more widespread reason that the studies we reviewed covered a relatively limited range of scales is likely to be restrictions on the resources required for sampling at relevant scales. In particular, integrating from fine resolution to broad extent in the same study poses challenges due to the general trade‐off between resolution and extent.

A number of solutions exist to overcome resource limitations associated with obtaining both fine‐resolution and broad‐extent data on juvenile habitat quality. Some processes can be resolved at microhabitat scales and then integrated to identify combined impacts at broader scales (Chapman, 1998; Hovel & Fonseca, 2005; Rooker, Holt & Holt, 1998). Models can be used as a platform for integration (Champagnat *et al*., 2024; Gernez *et al*., 2023; Vinagre *et al*., 2006a), but this still requires the synthesis of data from multiple studies. Methods that are high throughput but also able to detect fine‐resolution differences can provide broad yet detailed coverage, best minimising the trade‐offs between resolution and extent. For example, high‐throughput, fine‐resolution indices of individual growth were critical for revealing patterns and causes of growth variation in European plaice *Pleuronectes platessa* across diverse spatial (500 m – 100 km) and temporal (days – years) scales (Ciotti *et al*., 2013b). Similarly, the combination of measures (Section III.3) with different resolution provides an alternative option to characterise patterns at contrasting scales. For example, gut contents reflect feeding at the scale of a few hours and have been used to understand how specific saltmarshes support growth of juvenile European seabass *Dicentrarchus labrax* (Fonseca *et al*., 2011), while characterisation of growth variation among estuaries at a coastwide scale has been approached using biochemical indices, morphometric condition factor and otolith daily increment analysis (Vasconcelos *et al*., 2009; Vinagre *et al*., 2009a). Finally, rigorous use of sampling design theory and practice (Connolly, 1999; McAllister & Peterman, 1992) enables the spatial and temporal nesting of measures within a study and thus robust scaling up of results. For example, using nested designs to characterise dominant spatial and temporal scales of variation in juvenile growth can focus subsequent research on the scales at which key processes underlying habitat quality operate (Ciotti *et al*., 2013, 2013). Considering scale at the design stage will facilitate integration among studies, enabling coverage of broad spatial areas and long time series that are critical to understand influences of environmental variability and climate change on habitat.

### (3) More use of multiple metrics and measures

We recommend that, to the extent possible, study designs consider the use of multiple metrics and multiple measures for each metric. Beck *et al*. (2001) also recommended combining as many of the four metrics of habitat quality (abundance, growth, survival and juvenile–adult contribution) as possible. Most studies we reviewed used a single metric, or when using multiple metrics it was often growth combined with abundance.

Combining metrics can help to build a more accurate assessment of juvenile habitat quality. Given that there can be interactions among metrics of abundance, growth and survival, consequences for habitat quality are hard to predict based on assessment of one metric alone (Box 1). Comparisons among metrics, and particularly ground truthing against juvenile–adult contributions, can identify which are suitable proxies for juvenile habitat quality (see Section III.1). Even when contribution rates can be measured directly, complementary data on abundance, growth and/or survival still allow resolution of underlying processes at finer scales than measures of juvenile–adult contribution currently permit. It may not be possible to resolve the contribution made by a specific habitat to the adult population, but inferences can be drawn at these scales based on measurements of abundance, growth or survival. Similarly, a mechanistic understanding of how juvenile–adult contribution is controlled by habitat effects on abundance, growth and survival (Fodrie & Levin, 2008; Vasconcelos *et al*., 2011a) permits general insights and application to new locations and future scenarios (Champagnat *et al*., 2024; Fodrie & Levin, 2008). Finally, the combination of sensitive genomic and otolith chemistry methods provides an approach to “close the loop” on the life‐history connectivity of species: population genomics informs on the contribution of different spawning stocks to juvenile habitats, otolith chemistry identifies how these juvenile habitats contribute to adult populations and the combination of both can reveal how juvenile habitats moderate meta‐population dynamics (Ulrich *et al*., 2017).

Within each metric, combining measures provides more robust and informative assessments of habitat quality than single measures used in isolation. Multiple measures provide complementary lines of evidence and permit limitations associated with any one method to be overcome. Valid inferences from the two dominant measures of survival – tethering and cohort analysis – rely on multiple critical assumptions that are difficult to test: combining both measures could allow assessment of whether inferences are robust. Even when there is confidence in a single measure, this may only partially represent the process of interest, whereas a combination of measures can reveal the full picture. For example, measures relating both to growth (RNA‐based indices) and to energy storage (lipid content) were necessary to interpret changes in nutritional status of juvenile European plaice *P. platessa* (Ciotti *et al*., 2013a). Similarly, different measures were employed to separate predation (tethering study) and environmental stress (caging study) as agents of mortality in the coffee bean snail *Melampus bidentatus* (Johnson & Williams, 2017). As previously discussed, using different methods to measure single metrics also extends inferences across spatiotemporal scales, such as hourly scale variation inferred from diet analysis to integrated metrics of trophic status from geochemical analysis of tissues (Day *et al*., 2021; Woodland & Secor, 2011) or fine‐resolution (video survey) *versus* broad‐extent (beam trawl) estimates of abundance (Diaz, Cutter & Able, 2003). Critically, integration of multiple measures allows standardisation and comparison across habitat types that are typically sampled with different approaches (e.g. intertidal saltmarsh *versus* subtidal seagrass; Able, 1999).

Calculation of production is a powerful means to integrate abundance, growth and survival around a practical approximation/complementation for juvenile–adult contribution. Ley & Rolls (2018), for example, integrated metrics to estimate production of common snook *Centropomus undecimalis* in the Little Manatee River, Florida, USA. Few papers in our review reported production and those that did differed in approach. We stress the importance of reporting clear definitions, calculations and units: in our reviewed studies, the term “production” was often used loosely, not defined, and estimated by density rather than rate of change in biomass or abundance per unit area.

### (4) Accelerate field‐testing of emerging methods and development of new methods

Technology related to field sampling for habitat assessment is advancing rapidly (Brownscombe *et al*., 2022; Murphy & Jenkins, 2010) and we urge that investigators view long‐term and short‐term studies as opportunities to field‐test and explore promising new methods. A balance is needed between the continued application of existing methods, important for maintaining long‐term data sets and making broad comparisons, and new methods that overcome present limitations. Understanding how juvenile abundance, growth and survival interact to determine contribution to adult populations is complex, especially within dynamic coastal seascapes. While options exist to measure abundance and growth, easily applied methods may not provide inferences at suitable scales (Section III.3). Meanwhile, limited options exist to measure survival and juvenile–adult contribution, metrics that are important for accurately assessing juvenile habitat quality. Climate change creates previously unobserved (i.e. novel) conditions (Starzomski, 2013) that likely require concomitant advances in the design of habitat metrics and their measures. Technological advances can reduce trade‐offs in resource allocation, or they can facilitate new lines of enquiry into metrics: telemetry and video methods, for example, can increase spatiotemporal resolution and coverage beyond the limits imposed by capture sampling (Tamminga *et al*., 2015). Method development is therefore important for progress in assessing juvenile habitat quality.

The need for technical development pertains to both testing and calibrating emerging methods and to developing measures that leverage new technologies. In the past 20 years, new methods and innovative techniques have been adopted to measure juvenile habitat quality, such as video technology and acoustic telemetry (Brownscombe *et al*., 2022), but many remain in the “proof of concept” stage and have not reached their full potential or have yet sufficiently to overcome key limitations. For example, although acoustic telemetry is a powerful technique for understanding patterns of habitat use, it remains restricted to larger individuals and extending its application to common juvenile size classes requires advances in tag miniaturisation. Similarly, underwater video promises to increase spatial and temporal coverage, but existing systems (e.g. Baited Remote Underwater Video Systems, BRUVS) are not fully adapted to juvenile stages and advances in automated computer vision and AI are only just beginning to overcome observer time and video‐processing bottlenecks. Rapid developments in robotics and automation may push these technologies to exciting new capabilities (Dallolio *et al*., 2022). An example of a seemingly under‐used technology relates to the insights potentially gained about physiological states from molecular and “omics” techniques, which have revolutionised many fields of biology (Page & Lawley, 2022). Innovation is necessary to develop new options to measure survival since few suitable approaches exist.

### (5) Integrate habitat contributions over ontogeny

In order to draw appropriate inferences at the population scale, we recommend that studies of juvenile habitat quality are designed and interpreted with consideration of the broader ontogenetic context. This requires recognition of both the complexity of habitat use within the juvenile stage, during which habitat needs and demographic consequences of variation in habitat quality may change substantially, but also the regulatory role of the juvenile stage relative to other stages across the full life cycle.

Juveniles are often considered as comprising a single uniform stage, but in reality juveniles develop though a series of sub‐stages each with contrasting habitat needs (Bartolino *et al*., 2008; Nagelkerken *et al*., 2015; Sheaves *et al*., 2015). These sub‐stages can be defined by habitats used, shifts in diet, changes in growth rates, and other consistent and identifiable morphological or functional differences (Kaufman *et al*., 1992; Lo, Smith & Butler, 1995). Influences on population dynamics also change. Juvenile mortality typically declines exponentially with body size (Le Pape & Bonhommeau, 2015), and the influence of habitat on vital rates can shift dramatically as juveniles develop, often with high and variable post‐settlement mortality decoupling early juvenile abundance estimates from recruitment (Boyce *et al*., 2016). In some cases, the processes determining year‐class strength are restricted to short events spanning days or weeks (Holbrook & Schmitt, 2002; Steele & Forrester, 2002; van der Veer, Pihl & Bergman, 1990). As a consequence, habitat interactions occurring at one sub‐stage are not necessarily applicable to the juvenile stage as a whole or scalable to population‐level consequences.

In order to account for changing habitat needs throughout ontogeny, research programmes must either gather information about all juvenile sub‐stages or try to prioritise those of most interest. Gaining a comprehensive understanding of habitat requires extensive sampling in time and space (see Section III.2) and combining different metrics and measures adapted to different sub‐stages and habitats (see Section III.3). Efficiencies can, however, be gained by focusing on those juvenile sub‐stages that are of particular interest [e.g. habitat restoration (Champagnat *et al*., 2024; Puckett & Eggleston, 2012)] or that represent population bottlenecks with greatest influence on year‐class strength (e.g. habitat limitation; Le Pape & Bonhommeau, 2015). Empirical data, theory, conceptual models, and quantitative models, especially those that resolve sub‐stages within the juvenile stage, provide clues as to when population bottlenecks occur (Cantin & Post, 2018; Le Pape & Bonhommeau, 2015). For example, the concentration hypothesis (Iles & Beverton, 2000) uses simple life‐history characteristics to identify species for which year‐class strength is determined by compensatory density dependence through habitat limitation during the early juvenile stage (Archambault *et al*., 2014; Le Pape *et al*., 2003b). Highly resolved life‐cycle models can also be used to identify habitat requirements and limitations during key life stages and sub‐stages (Rose *et al*., 2018; van de Wolfshaar *et al*., 2015).

It is also important to put studies in context of the leverage that the juvenile stage has, relative to other life stages, over the population dynamics of the species investigated. The relative importance of juvenile stage‐specific processes in determining population dynamics can vary among taxa (Archambault *et al*., 2014; Houde, 1987), and conclusions need to be appropriately constrained in cases where there is ultimately little influence of juvenile habitat quality on variation in population size. Life‐cycle models can provide information on the role of juvenile stage bottlenecks relative to other life stages (Archambault *et al*., 2014; Dahlke *et al*., 2020; Fodrie *et al*., 2009; Levin & Stunz, 2005; Petitgas *et al*., 2013; van de Wolfshaar, HilleRisLambers & Gårdmark, 2011) and quantify population‐level impacts of changes in habitat quantity or quality (Rahikainen *et al*., 2017; Rose *et al*., 2018; Ruiz *et al*., 2009). For instance, Rochette *et al*. (2013) and Archambault *et al*. (2016) developed a life‐cycle model of the common sole *Solea solea* in the eastern English Channel to estimate juvenile–adult contribution. The model was then used to infer consequences of juvenile habitat degradation (Archambault *et al*., 2018; Champagnat *et al*., 2021) and restoration (Champagnat *et al*., 2024; Gernez *et al*., 2023) at population and fishery scales.

## IV. CONCLUSIONS

(1) Over 20 years since publication, there remains general agreement with Beck *et al*. (2001) that the quality of juvenile habitat is best measured as the contribution to the adult population (i.e. juvenile–adult contribution), itself underpinned by habitat‐specific abundance, growth and survival. Our review of marine and estuarine fish, crustaceans and molluscs over the period before and after publication of Beck *et al*. (2001) does not indicate a shift towards this approach. There has been an increase in studies of juvenile–adult contribution, but these remain rare, with most research still relying on measures of abundance.
(2) Studies generally focus on approaches that, despite being readily accessible, offer limited insights into the spatial and temporal complexities of “seascape” processes. Insights are further limited by the concentration of studies around a few geographic regions and taxa. Robust habitat science to support ecosystem‐based approaches to fisheries management must be emphasised and focused on utilising and expanding the range of available methods to measure habitat quality across diverse species, geographical locations, habitats and spatiotemporal scales.
(3) There exists an inevitable conflict between the complexity of fish–habitat interactions revealed by research and the simplicity of habitat quality indicators that can be practically considered by managers. Although management tools must unavoidably operate on a simplified model of reality, these models must be founded in a detailed mechanistic understanding of how juvenile habitats mediate interactions among abundance, growth and survival to determine contributions of juvenile habitats to adult populations. Put simply, we can have little confidence in estimates of habitat quality built from measures of abundance alone, unless we know why the juveniles are there. The assumption that habitats with high abundance produce the greatest contribution to adult populations is unlikely to be met universally, and too often remains unchallenged. While abundance monitoring is an important element of the evidence base, hypothesis‐driven studies integrating multiple metrics of habitat quality are critically needed for effective prediction and sound decision‐making.

## Supporting information


**Appendix S1.** Methods.
**Fig. S1.** PRISMA flow diagram for papers included in the systematic review.
**Appendix S2.** Papers included in the quantitative synthesis.
**Table S1.** Categories for methods used to measure abundance as a metric of juvenile habitat quality.
**Table S2.** Categories for methods used to measure growth as a metric of juvenile habitat quality.
**Table S3.** Categories for methods used to measure survival as a metric of juvenile habitat quality.
**Table S4.** Categories for methods used to measure juvenile–adult contribution as a metric of juvenile habitat quality.
