# Supplementary material for: Measuring juvenile habitat quality for fishes and invertebrates
Source: Biol Rev Camb Philos Soc. 2025 Jul 30;100(6):2346–95. doi: 10.1111/brv.70050 (PMC12586312; doi:10.1111/brv.70050)
Supplement: Supplementary file 1 — Appendix S1. Methods. Fig. S1. PRISMA flow diagram for papers included in the systematic review. Appendix S2. Papers included in the quantitative synthesis. Table S1. Categories for methods used to measure abundance as a metric of juvenile habitat quality. Table S2. Categories for methods used to measure growth as a metric of juvenile habitat quality. Table S3. Categories for methods used to measure survival as a metric of juvenile habitat quality. Table S4. Categories for methods used to measure juvenile–adult contribution as a metric of juvenile habitat quality. [file BRV-100-2346-s001.docx]

Ciotti et al.: Measuring juvenile habitat quality for fishes and invertebrates

Supporting Information

**Appendix S1: Methods**

We performed a search of the scientific literature to obtain papers measuring habitat quality for juvenile fishes and invertebrates. We focused on practical, *in situ* approaches rather than studies based exclusively on modelling or laboratory experiments. We included studies of marine or estuarine fish, crustaceans and molluscs: this covers most commercially important species and those with a life cycle that includes discrete juvenile habitat but does miss some relevant taxa such as bait worms (Flach & Beukema, 1994). We did not include salmonids, many of which have a specific life-history pattern, with heavy reliance on fresh water, and which are subject to a substantial and independent literature.

The search was conducted on two dates, 27th June 2019 and 3rd July 2021, using the *Web of Science* Core Collection with the following terms:

TS = (nurser* OR "habitat complex*" OR EJH OR "juvenile habitat" OR "structural* complex*" OR EFH)

AND

TS = (marine OR estuar* OR coast* OR nearshore OR seascape OR seagrass* OR SAV OR mangrove* OR marsh* OR saltmarsh* OR wetland* OR kelp* OR macrophyte* OR lagoon* OR brackish OR sand* OR mud* OR tidal* OR soft*bottom)

AND

TS = (juvenile* OR recruit* OR post-larva* OR post-settle* OR sub-adult* OR young OR YOY OR anadromous OR age-0 OR natal OR pup OR fry OR fingerling OR smelt)

AND

TS = (growth OR surviv* OR recruit* OR densit* OR abundance* OR product* OR movement OR connect* OR emigrat* OR migrat* OR condition)

After removal of duplicates, both searches returned a total of 3,768 unique papers. The *Web of Science* Core Collection starts in 1970, but the search found no papers prior to 1982 suggesting that the bulk of contemporary literature on juvenile habitat quality had been captured. Papers were then screened in three steps against pre-determined selection criteria (Fig. S1) in order to remove those that did not meet the aim of the review.

First, we performed title screening by reading through a randomly ordered list of titles, to remove any that were clearly out of the scope of the selection criteria (Fig. S1). Any paper of potential marginal relevance was retained at this stage, until more information was available (i.e. abstract, full text) to make a better assessment. At least two of the authors screened every title, and the paper was retained in cases of disagreement.

Second, we applied the criteria to abstracts (Fig. S1). All 13 authors performed abstract screening, and initial cross-calibration was undertaken to ensure that the criteria were defined clearly and applied consistently. In order to achieve this, all authors screened a common set of 15 abstracts and then met to compare decisions and refine the criteria. Multivariate analyses were performed and discussed to identify dissimilarities among authors. This process was repeated for a further two sets of 15 abstracts and a final set of 50. Abstracts were then randomly assigned to 12 of the authors for screening proper: each abstract was viewed by two authors and the thirteenth author made the final decision in the event of disagreement. Papers that did not contain an abstract were automatically retained.

The third step was to screen the papers based on the full text (Fig. S1). Papers that were retained after abstract screening were re-distributed randomly to authors for this task. Given that authors had substantial prior experience from abstract screening and access to the full-text made the criteria easier to assess, each paper was viewed by a single author at this stage. An additional 12 papers were excluded at the full-text screening stage because they could not be accessed or were in a language not spoken by the authors, leaving 874 papers, which formed the basis of this review.

Once screening had been completed, a pre-defined set of information was extracted from each paper to meet the aim and objectives of the review. This task was completed by the authors and involved reading the end of the introduction (to understand the context of the study) as well as the methods and results sections to obtain the relevant information. Data extraction focused on the species, location, years, habitat type, spatial/temporal extent/resolution of sampling, and the methods used to measure juvenile habitat quality. Methods were categorised at two levels. First, we established which of the four ‘metrics’ of juvenile habitat quality, as formulated by Beck *et al.* (2001), had been applied: abundance, growth, survival or juvenile–adult contribution. Within the growth metric, we included not only direct measures of growth, but also those reflecting energetic status, nutritional condition, and feeding. Juvenile–adult contribution refers to measurements of the contribution of juveniles from a given habitat to the adult population and is sometimes called ‘connectivity’. Second, we recorded ‘measures’, or methods used to quantify each metric, according to predetermined categories (Tables S1 – S4). Data were extracted to a common Access database form. In the case of categorical data, the database was pre-populated with options agreed in advance by the authors, but free text was available for when information fell outside pre-determined categories. Geographic location was recorded with sufficient resolution to position the study on a world map and was obtained either from written coordinates or by reading maps. Geographic location was not recorded for six studies where isolation of single representative points in space was not possible (e.g. study sites spanned different ocean basins). Spatial resolution was defined as the linear distance (km), by water, between neighbouring locations at the finest scale of comparison. Spatial extent was the linear distance (km), by water, across the entire sampling region being compared.​ Temporal resolution was defined as the time interval between neighbouring timepoints at the finest scale of comparison. Temporal extent was the total duration between the first and last sampling occasions for a study. Even though papers could involve multiple species and multiple metrics sampled with different experimental designs, data were only recorded at the level of the paper and not split by each individual metric or habitat. For example, if a study measured growth in species A and abundance in species B we would record that the study measured growth and abundance and focused on species A and B.

Following extraction, data were checked, processed and analysed in R (R Development Core Team, 2023). Since not all papers fitted the pre-defined options for some data fields, categories were re-considered after data extraction, to consolidate multiple categories or establish new ones, as appropriate. Species names were synonymised and full taxonomies were constructed against the NCBI Taxonomy Database (https://www.ncbi.nlm.nih.gov/taxonomy) using *taxize* (Chamberlain *et al.*, 2020) and *taxizedb* (Chamberlain & Arendsee, 2021) packages on 24th September 2023. Study locations were grouped into FAO Major Fishing Areas using shapefiles downloaded on 17th August 2021 from <https://data.apps.fao.org/map/catalog/srv/eng/catalog.search#/metadata/ac02a460-da52-11dc-9d70-0017f293bd28>. Data were summarised by taking the sum of articles per metric, and per measure within each metric. These sums are visually represented as the total number of articles published for each metric/measure, both overall and for each year. Furthermore, percentages were calculated for each measure per year relative to the total number of occurrences of the respective metric in the given year. Since individual papers often employed multiple approaches, the total occurrences exceeded the number of papers published. Similar approaches were applied to represent the distribution of studies across taxonomic groups, habitats, FAO Major Fishing Areas, and spatiotemporal scales.

**REFERENCES**

Beck, M. W., Heck, K. L., Able, K. W., Childers, D. L., Eggleston, D. B., Gillanders, B. M., Halpern, B., Hays, C. G., Hoshino, K., Minello, T. J., Orth, R. J., Sheridan, P. F. & Weinstein, M. P. (2001). The identification, conservation, and management of estuarine and marine nurseries for fish and invertebrates. *BioScience* **51**(8), 633–641.

Chamberlain, S. & Arendsee, Z. (2021). taxizedb: Tools for working with 'taxonomic' databases (Version R package version 0.3.0). Retrieved from https://CRAN.R-project.org/package=taxizedb

Chamberlain, S., Szoecs, E., Foster, Z., Arendsee, Z., Boettiger, C., Ram, K., Bartomeus, I., Baumgartner, J., O'Donnell, J., Oksanen, J., Tzovaras, B. G., Marchand, P., Tran, V., Salmon, M., Li, G. & Grenié, M. (2020). taxize: Taxonomic information from around the web (Version R package version 0.9.98). Retrieved from https://github.com/ropensci/taxize

Flach, E. C. & Beukema, J. J. (1994). Density-governing mechanisms in populations of the lugworm *Arenicola marina* on tidal flats. *Marine Ecology Progress Series* **115**(1-2), 139–149.

R Development Core Team. (2023). R: A language and environment for statistical computing (Version Version 4.3.0). Vienna, Austria. Retrieved from http://www.R-project.org


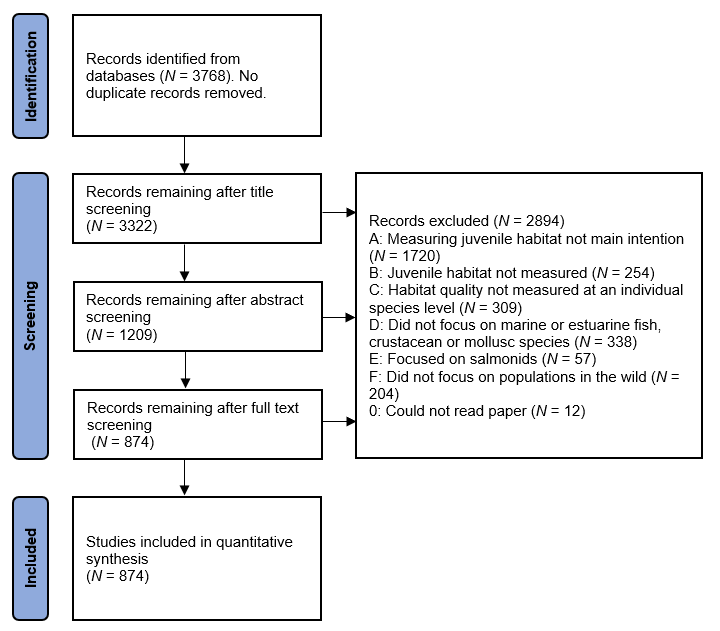
 **Fig. S1.** PRISMA flow diagram for papers included in the systematic review. Papers were excluded based on six criteria, A – F. A: since we wanted to assess the methods that had been used to measure juvenile habitat quality, we excluded papers where this was not the main intention of the paper. Studies of connectivity between the juvenile stage and other life-history stages (e.g. studies of larval dispersal and genetic connectivity) were only included where there was a main focus on quality of juvenile habitats. Studies that focused exclusively on comparing spawning locations or adult habitats were excluded using this criterion. B: the emphasis of the review is on methods to measure juvenile habitat quality, so we excluded papers that did not involve data collection, such as purely review, theoretical, method development or modelling studies. C: the review focuses on methods used to measure juvenile habitat quality for single species, so we excluded those focusing on community- or assemblage-level metrics (e.g. diversity or assemblage structure). Studies of multiple species were included if separate assessments of habitat quality were made for each. D: given the breadth of related general ecological literature about habitat use, we restricted the review to key commercial groups that typically have distinct juvenile habitats. We also focused solely on marine and estuarine systems, given the inherent differences from the biology and physical environment in freshwater systems. Under this criterion we therefore excluded anything that was not a marine or estuarine fish, crustacean or mollusc. E: we excluded salmonids since they have a unique life history, often occupy fresh water during juvenile stages and are the subject of a large and somewhat independent body of research. F: the emphasis of the review is on methods to measure juvenile habitat quality *in situ*, so we excluded papers that studied populations in the laboratory. Studies focusing on cultured populations in the wild (e.g. evaluating stock enhancement) were included provided that this involved measurement of juvenile habitat quality (rather than comparing culture or stocking protocols for example). Exclusion criteria are not mutually exclusive: the most common exclusion criterion across reviewers was used to generate the totals in the diagram, but if criteria were equally common the reason was recorded in the order A–F.

**Appendix S2. Papers included in the quantitative synthesis**

Able, K. W., Grothues, T. M., Shaw, M. J., VanMorter, S. M., Sullivan, M. C. & Ambrose, D. D. (2020). Alewife (*Alosa pseudoharengus*) spawning and nursery areas in a sentinel estuary: spatial and temporal patterns. *Environmental Biology of Fishes* **103**(11), 1419–1436.

Able, K. W., Grothues, T. M., Turnure, J. T., Byrne, D. M. & Clerkin, P. (2012). Distribution, movements, and habitat use of small striped bass (*Morone saxatilis*) across multiple spatial scales. *Fishery Bulletin* **110**(2), 176–192.

Able, K. W., Hagan, S. M. & Brown, S. A. (2003). Mechanisms of marsh habitat alteration due to phragmites: Response of young-of-the-year Mummichog (*Fundulus heteroclitus*) to treatment for Phragmites removal. *Estuaries* **26**(2B), 484–494.

Abookire, A. A. & Norcross, B. L. (1998). Depth and substrate as determinants of distribution of juvenile flathead sole (*Hippoglossoides elassodon*) and rock sole (*Pleuronectes bilineatus*), in Kachemak Bay, Alaska. *Journal of Sea Research* **39**(1–2), 113–123.

Aburto-Oropeza, O., Dominguez-Guerrero, I., Cota-Nieto, J. & Plomozo-Lugo, T. (2009). Recruitment and ontogenetic habitat shifts of the yellow snapper (*Lutjanus argentiventris*) in the Gulf of California. *Marine Biology* **156**(12), 2461–2472.

Acosta, C. A. (1999). Benthic dispersal of Caribbean spiny lobsters among insular habitats: Implications for the conservation of exploited marine species. *Conservation Biology* **13**(3), 603–612.

Acosta, C. A. & Butler, M. J. (1997). Role of mangrove habitat as a nursery for juvenile spiny lobster, *Panulirus argus*, in Belize. *Marine and Freshwater Research* **48**(8), 721–727.

Adams, A. J. & Ebersole, J. P. (2002). Use of back-reef and lagoon habitats by coral reef fishes. *Marine Ecology Progress Series* **228**, 213–226.

Adams, A. J. & Ebersole, J. P. (2004). Processes influencing recruitment inferred from distributions of coral reef fishes. *Bulletin of Marine Science* **75**(2), 153–174.

Adams, A. J., Locascio, J. V. & Robbins, B. D. (2004). Microhabitat use by a post-settlement stage estuarine fish: evidence from relative abundance and predation among habitats. *Journal of Experimental Marine Biology and Ecology* **299**(1), 17–33.

Adams, A. J., Wolfe, R. K., Kellison, G. T. & Victor, B. C. (2006). Patterns of juvenile habitat use and seasonality of settlement by permit, *Trachinotus falcatus*. *Environmental Biology of Fishes* **75**(2), 209–217.

Adnan, N. A., Loneragan, N. R. & Connolly, R. M. (2002). Variability of, and the influence of environmental factors on, the recruitment of postlarval and juvenile *Penaeus merguiensis* in the Matang mangroves of Malaysia. *Marine Biology* **141**(2), 241–251.

Afonso, A. S. & Gruber, S. H. (2007). Pueruli settlement in the Caribbean spiny lobster, *Panulirus argus*, at Bimini, Bahamas. *Crustaceana* **80**(11), 1355–1371.

Afonso, P., Tempera, F. & Menezes, G. (2008). Population structure and habitat preferences of red porgy (*Pagrus pagrus*) in the Azores, central North Atlantic. *Fisheries Research* **93**(3), 338–345.

Aguilar, C., Gonzalez-Sanson, G., Cabrera, Y., Ruiz, A. & Curry, R. A. (2014). Inter-habitat variation in density and size composition of reef fishes from the Cuban Northwestern shelf. *Revista De Biologia Tropical* **62**(2), 589–602.

Aguilar, M. & Stotz, W. B. (2000). Settlement sites of juvenile scallops *Argopecten purpuratus* (Lamarck, 1819) in the subtidal zone at Puerto Aldea, Tongoy Bay, Chile. *Journal of Shellfish Research* **19**(2), 749–755.

Aguirre, J. D. & McNaught, D. C. (2011). Habitat modification affects recruitment of abalone in central New Zealand. *Marine Biology* **158**(3), 505–513.

Al-Wazzan, Z., Vay, L. L., Behbehani, M. & Gimenez, L. (2020). Scale-dependent spatial and temporal patterns of abundance and population structure of the xanthid crab *Leptodius exaratus* on rocky shores in Kuwait. *Regional Studies in Marine Science* **37**, 101325.

Albaina, A., Taylor, M. I. & Fox, C. J. (2012). Molecular detection of plaice remains in the stomachs of potential predators on a flatfish nursery ground. *Marine Ecology Progress Series* **444**, 223–238.

Alberts-Hubatsch, H., Lee, S. Y., Diele, K., Wolff, M. & Nordhaus, I. (2014). Microhabitat use of early benthic stage mud crabs, *Scylla serrata* (Forskal, 1775), in Eastern Australia. *Journal of Crustacean Biology* **34**(5), 604–610.

Alemany, F., Alvarez, I., García, A., Cortés, D., Ramírez, T., Quintanilla, J., Alvarez, F. & Rodríguez, J. M. (2006). Postflexion larvae and juvenile daily growth patterns of the Alboran Sea sardine (*Sardina pilchardus* Walb.): influence of wind. *Scientia Marina* **70**, 93–104.

Alford, S. B. & Rozas, L. P. (2019). Effects of nonnative Eurasian Watermilfoil, *Myriophyllum spicatum*, on nekton habitat quality in a Louisiana oligohaline estuary. *Estuaries and Coasts* **42**(3), 613–628.

Allen, M. J. & Herbinson, K. T. (1990). Settlement of juvenile California halibut, *Paralichthys californicus*, along the coasts of Los Angeles, Orange, and San Diego counties in 1989. *California Cooperative Oceanic Fisheries Investigations Reports* **31**, 84–96.

Allen, R. L. & Baltz, D. M. (1997). Distribution and microhabitat use by flatfishes in a Louisiana estuary. *Environmental Biology of Fishes* **50**(1), 85–103.

Almeida, A. C., Baeza, J. A., Fransozo, V., Castilho, A. L. & Fransozo, A. (2012). Reproductive biology and recruitment of *Xiphopenaeus kroyeri* in a marine protected area in the Western Atlantic: implications for resource management. *Aquatic Biology* **17**(1), 57–69.

Almeida, M. J., Flores, A. A. V. & Queiroga, H. (2008). Effect of crab size and habitat type on the locomotory activity of juvenile shore crabs, *Carcinus maenas*. *Estuarine Coastal and Shelf Science* **80**(4), 509–516.

Almeida, M. J., Gonzalez-Gordillo, J. I., Flores, A. A. V. & Queiroga, H. (2011). Cannibalism, post-settlement growth rate and size refuge in a recruitment-limited population of the shore crab *Carcinus maenas*. *Journal of Experimental Marine Biology and Ecology* **410**, 72–79.

Alonso, V. A. G., Diaz, M. V., Pajaro, M. & Capitanio, F. L. (2021). Ontogeny versus environmental forcing off the Southwest Atlantic Ocean: Nutritional condition of Fuegian sprat (*Sprattus fuegensis*) early stages. *Fisheries Oceanography* **30**, 653–665.

Amara, R. (2004). 0-group flatfish growth conditions on a nursery ground (Bay of Canche, Eastern English Channel). *Hydrobiologia* **518**(1–3), 23–32.

Amara, R., Meziane, T., Gilliers, C., Hermell, G. & Laffargue, P. (2007). Growth and condition indices in juvenile sole *Solea solea* measured to assess the quality of essential fish habitat. *Marine Ecology Progress Series* **351**, 201–208.

Amara, R. & Paul, C. (2003). Seasonal patterns in the fish and epibenthic crustaceans community of an intertidal zone with particular reference to the population dynamics of plaice and brown shrimp. *Estuarine Coastal and Shelf Science* **56**(3–4), 807–818.

Amara, R., Selleslagh, J., Billon, G. & Minier, C. (2009). Growth and condition of 0-group European flounder, *Platichthys flesus* as indicator of estuarine habitat quality. *Hydrobiologia* **627**(1), 87–98.

Amaral, V., Cabral, H. N., Jenkins, S., Hawkins, S. & Paula, J. (2009). Comparing quality of estuarine and nearshore intertidal habitats for *Carcinus maenas*. *Estuarine Coastal and Shelf Science* **83**(2), 219–226.

Amaral, V., Cabral, H. N. & Paula, J. (2008). Implications of habitat-specific growth and physiological condition on juvenile crab population structure. *Marine and Freshwater Research* **59**(8), 726–734.

Amorim, E., Ramos, S., Elliott, M. & Bordalo, A. A. (2016). Immigration and early life stages recruitment of the European flounder (*Platichthys flesus*) to an estuarine nursery: The influence of environmental factors. *Journal of Sea Research* **107**, 56–66.

Amorim, E., Ramos, S., Elliott, M. & Bordalo, A. A. (2018). Dynamic habitat use of an estuarine nursery seascape: Ontogenetic shifts in habitat suitability of the European flounder (*Platichthys flesus*). *Journal of Experimental Marine Biology and Ecology* **506**, 49–60.

Andersen, A. K., Schou, J., Sparrebohn, C. R., Nicolajsen, H. & Stottrup, J. G. (2005). The quality of release habitat for reared juvenile flounder, *Platichthys flesus*, with respect to salinity and depth. *Fisheries Management and Ecology* **12**(3), 211–219.

Anderson, J. M., Burns, E. S., Meese, E. N., Farrugia, T. J., Stirling, B. S., White, C. F., Logan, R. K., O'Sullivan, J., Winkler, C. & Lowe, C. G. (2021). Interannual nearshore habitat use of young of the year white sharks off Southern California. *Frontiers in Marine Science* **8**, 645142.

Anstead, K. A., Schaffler, J. J. & Jones, C. M. (2016). Coast-wide nursery contribution of new recruits to the population of Atlantic menhaden. *Transactions of the American Fisheries Society* **145**(3), 627–636.

Anstead, K. A., Schaffler, J. J. & Jones, C. M. (2017). Contribution of nursery areas to the adult population of Atlantic menhaden. *Transactions of the American Fisheries Society* **146**(1), 36–46.

Arena, P. T., Quinn, T. P., Jordan, L. K. B., Sherman, R. L., Harttung, F. M. & Spieler, R. E. (2004). Presence of juvenile blackfin snapper, *Lutjanus buccanella*, and snowy grouper, *Epinephelus niveatus*, on shallow-water artificial reefs. *Proceedings of the Gulf and Caribbean Fisheries Institute* **55**, 700–712.

Ariyama, H. & Secor, D. H. (2010). Effect of environmental factors, especially hypoxia and typhoons, on recruitment of the gazami crab *Portunus trituberculatus* in Osaka Bay, Japan. *Fisheries Science* **76**(2), 315–324.

Armstrong, D. A., Rooper, C. & Gunderson, D. (2003). Estuarine production of juvenile Dungeness crab (*Cancer magister*) and contribution to the Oregon-Washington coastal fishery. *Estuaries* **26**(4B), 1174–1188.

Aschenbrenner, A., Hackradt, C. W. & Ferreira, B. P. (2016). Spatial variation in density and size structure indicate habitat selection throughout life stages of two Southwestern Atlantic snappers. *Marine Environmental Research* **113**, 49–55.

Asjes, A., Gonzalez-Irusta, J. M. & Wright, P. J. (2016). Age-related and seasonal changes in haddock *Melanogrammus aeglefinus* distribution: implications for spatial management. *Marine Ecology Progress Series* **553**, 203–217.

Avigliano, E., Pisonero, J., Bordel, N., Domanico, A. & Volpedo, A. V. (2019). Mixed-stock and discriminant models use for assessing recruitment sources of estuarine fish populations in La Plata Basin (South America). *Journal of the Marine Biological Association of the United Kingdom* **99**(6), 1429–1433.

Aylesworth, L. A., Xavier, J. H., Oliveira, T. P. R., Tenorio, G. D., Diniz, A. F. & Rosa, I. L. (2015). Regional-scale patterns of habitat preference for the seahorse *Hippocampus reidi* in the tropical estuarine environment. *Aquatic Ecology* **49**(4), 499–512.

Bacheler, N. M., Paramore, L. M., Buckel, J. A. & Hightower, J. E. (2009*b*). Abiotic and biotic factors influence the habitat use of an estuarine fish. *Marine Ecology Progress Series* **377**, 263–277.

Baillie, C. J., Fear, J. M. & Fodrie, F. J. (2015). Ecotone effects on seagrass and saltmarsh habitat use by juvenile nekton in a temperate estuary. *Estuaries and Coasts* **38**(5), 1414–1430.

Baker, R. & Minello, T. J. (2010). Growth and mortality of juvenile white shrimp *Litopenaeus setiferus* in a marsh pond. *Marine Ecology Progress Series* **413**, 95–104.

Baltus, C. A. M. & Van der Veer, H. W. (1995). Nursery areas of solenette *Buglossidium luteum* (Risso, 1810) and scaldfish *Arnoglossus laterna* (Walbaum, 1792) in the southern North Sea. *Netherlands Journal of Sea Research* **34**(1–3), 81–88.

Baltz, D. M., Fleeger, J. W., Rakocinski, C. F. & McCall, J. N. (1998). Food, density, and microhabitat: factors affecting growth and recruitment potential of juvenile saltmarsh fishes. *Environmental Biology of Fishes* **53**(1), 89–103.

Bangley, C. W., Paramore, L., Dedman, S. & Rulifson, R. A. (2018). Delineation and mapping of coastal shark habitat within a shallow lagoonal estuary. *PLoS One* **13**(4), e0195221.

Barbier, P., Meziane, T., Foret, M., Tremblay, R., Robert, R. & Olivier, F. (2017). Nursery function of coastal temperate benthic habitats: New insight from the bivalve recruitment perspective. *Journal of Sea Research* **121**, 11–23.

Barbour, A. B. & Adams, A. J. (2012). Biologging to examine multiple life stages of an estuarine-dependent fish. *Marine Ecology Progress Series* **457**, 241–250.

Barbour, A. B., Adams, A. J. & Lorenzen, K. (2014*a*). Emigration-corrected seasonal survival of a size-structured fish population in a nursery habitat. *Marine Ecology Progress Series* **514**, 191–205.

Barbour, A. B., Adams, A. J. & Lorenzen, K. (2014*b*). Size-based, seasonal, and multidirectional movements of an estuarine fish species in a habitat mosaic. *Marine Ecology Progress Series* **507**, 263–276.

Barioto, J. G., Stanski, G., Grabowski, R. C., Costa, R. C. & Castilho, A. L. (2017). Ecological distribution of *Penaeus schmitti* (Dendrobranchiata: Penaeidae) juveniles and adults on the southern coast of Sao Paulo state, Brazil. *Marine Biology Research* **13**(6), 693–703.

Bartol, I. K., Mann, R. & Luckenbach, M. (1999). Growth and mortality of oysters (*Crassostrea virginica*) on constructed intertidal reefs: effects of tidal height and substrate level. *Journal of Experimental Marine Biology and Ecology* **237**(2), 157–184.

Bartulovic, V., Glamuzina, B., Lucic, D., Conides, A., Jasprica, N. & Dulcic, J. (2007). Recruitment and food composition of juvenile thin-lipped grey mullet, *Liza ramada* (Risso, 1826), in the Neretva River estuary (Eastern Adriatic, Croatia). *Acta Adriatica* **48**(1), 25–37.

Beal, B. F., Coffin, C. R., Randall, S. F., Goodenow, C. A., Pepperman, K. E. & Ellis, B. W. (2020). Interactive effects of shell hash and predator exclusion on 0-year class recruits of two infaunal intertidal bivalve species in Maine, USA. *Journal of Experimental Marine Biology and Ecology* **530**, 151441.

Becker, A., Cowley, P. D. & Whitfield, A. K. (2010). Use of remote underwater video to record littoral habitat use by fish within a temporarily closed South African estuary. *Journal of Experimental Marine Biology and Ecology* **391**(1–2), 161–168.

Becker, A. & Taylor, M. D. (2017). Nocturnal sampling reveals usage patterns of intertidal marsh and subtidal creeks by penaeid shrimp and other nekton in south-eastern Australia. *Marine and Freshwater Research* **68**(4), 780–787.

Beggs, S. E. & Nash, R. D. M. (2007). Variability in settlement and recruitment of 0-group dab *Limanda limanda* L. in Port Erin Bay, Irish Sea. *Journal of Sea Research* **58**(1), 90–99.

Behringer, D. C., Butler, M. J., Herrnkind, W. F., Hunt, J. H., Acosta, C. A. & Sharp, W. C. (2009). Is seagrass an important nursery habitat for the Caribbean spiny lobster, *Panulirus argus*, in Florida? *New Zealand Journal of Marine and Freshwater Research* **43**(1), 327–337.

Belcher, C. N. & Jennings, C. A. (2010). Utility of mesohabitat features for determining habitat associations of subadult sharks in Georgia's estuaries. *Environmental Biology of Fishes* **88**(4), 349–359.

Berghahn, R., Ludemann, K. & Ruth, M. (1995). Differences in individual growth of newly settled O-group plaice (*Pleuronectes platessa* L) in the intertidal of neighbouring Wadden Sea areas. *Netherlands Journal of Sea Research* **34**(1–3), 131–138.

Bertelli, C. M. & Unsworth, R. K. F. (2014). Protecting the hand that feeds us: Seagrass (*Zostera marina*) serves as commercial juvenile fish habitat. *Marine Pollution Bulletin* **83**(2), 425–429.

Bertelsen, R. D., Butler, M. J., Herrnkind, W. F. & Hunt, J. H. (2009). Regional characterisation of hard-bottom nursery habitat for juvenile Caribbean spiny lobster (*Panulirus argus*) using rapid assessment techniques. *New Zealand Journal of Marine and Freshwater Research* **43**(1), 299–312.

Beukema, J. J. & Dekker, R. (2003). Redistribution of spat-sized *Macoma balthica* in the Wadden Sea in cold and mild winters. *Marine Ecology Progress Series* **265**, 117–122.

Beyst, B., Mees, J. & Cattrijsse, A. (1999). Early postlarval fish in the hyperbenthos of the Dutch Delta (south-west Netherlands). *Journal of the Marine Biological Association of the United Kingdom* **79**(4), 709–724.

Biagi, F., Gambaccini, S. & Zazzetta, M. (1998). Settlement and recruitment in fishes: the role of coastal areas. *Italian Journal of Zoology* **65**, 269–274.

Biole, F. G., Thompson, G. A., Vargas, C. V., Leisen, M., Barra, F., Volpedo, A. V. & Avigliano, E. (2019). Fish stocks of *Urophycis brasiliensis* revealed by otolith fingerprint and shape in the Southwestern Atlantic Ocean. *Estuarine Coastal and Shelf Science* **229**, 106406.

Blanc, A., Du Sel, G. P. & Daguzan, J. (1998). Habitat and diet of early stages of *Sepia officinalis* L. (Cephalopoda) in Morbihan Bay, France. *Journal of Molluscan Studies* **64**, 263–274.

Blanco-Martinez, Z. & Perez-Castaneda, R. (2017). Does the relative value of submerged aquatic vegetation for penaeid shrimp vary with proximity to a tidal inlet? Preliminary evidence from a subtropical coastal lagoon. *Marine and Freshwater Research* **68**(3), 581–591.

Blanco-Martinez, Z., Perez-Castaneda, R., Sanchez-Martinez, J. G., Benavides-Gonzalez, F., Rabago-Castro, J. L., Vazquez-Sauceda, M. D. & Garrido-Olvera, L. (2020). Density-dependent condition of juvenile penaeid shrimps in seagrass-dominated aquatic vegetation beds located at different distance from a tidal inlet. *Peerj* **8**, e10496.

Bolle, L. J., Dapper, R., Witte, J. I. & Vanderveer, H. W. (1994). Nursery grounds of dab (*Limanda limanda* L) in the southern North Sea. *Netherlands Journal of Sea Research* **32**(3–4), 299–307.

Bolle, L. J., Rijnsdorp, A. D. & van der Veer, H. W. (2001). Recruitment variability in dab (*Limanda limanda*) in the southeastern North Sea. *Journal of Sea Research* **45**(3–4), 255–270.

Bouchoucha, M., Brach-Papa, C., Gonzalez, J. L., Lenfant, P. & Darnaude, A. M. (2018). Growth, condition and metal concentration in juveniles of two Diplodus species in ports. *Marine Pollution Bulletin* **126**, 31–42.

Bourque, J. F., Dodson, J. J., Ryan, D. A. J. & Marcogliese, D. J. (2006). Cestode parasitism as a regulator of early life-history survival in an estuarine population of rainbow smelt *Osmerus mordax*. *Marine Ecology Progress Series* **314**, 295–307.

Boutin, B. P. & Targett, T. E. (2019). Density, growth, production, and feeding dynamics of juvenile weakfish (*Cynoscion regalis*) in Delaware Bay and salt marsh tributaries: spatiotemporal comparison of nursery habitat quality. *Estuaries and Coasts* **42**(1), 274–291.

Bradbury, I. R., Laurel, B. J., Robichaud, D., Rose, G. A., Snelgrove, P. V. R., Gregory, R. S., Cote, D. & Windle, M. J. S. (2008). Discrete spatial dynamics in a marine broadcast spawner: Re-evaluating scales of connectivity and habitat associations in Atlantic cod (*Gadus morhua* L.) in coastal Newfoundland. *Fisheries Research* **91**(2–3), 299–309.

Brady, D. C. & Targett, T. E. (2013). Movement of juvenile weakfish *Cynoscion regalis* and spot *Leiostomus xanthurus* in relation to diel-cycling hypoxia in an estuarine tidal tributary. *Marine Ecology Progress Series* **491**, 199–219.

Brame, A. B., McIvor, C. C., Peebles, E. B. & Hollander, D. J. (2014). Site fidelity and condition metrics suggest sequential habitat use by juvenile common snook. *Marine Ecology Progress Series* **509**, 255–269.

Bromilow, A. M. & Lipcius, R. N. (2017). Mechanisms governing ontogenetic habitat shifts: role of trade-offs, predation, and cannibalism for the blue crab. *Marine Ecology Progress Series* **584**, 145–159.

Brown, E. J., Kokkalis, A. & Stottrup, J. G. (2019). Juvenile fish habitat across the inner Danish waters: Habitat association models and habitat growth models for European plaice, flounder and common sole informed by a targeted survey. *Journal of Sea Research* **155**, 101795.

Brown, J. A. (2006). Using the chemical composition of otoliths to evaluate the nursery role of estuaries for English sole *Pleuronectes vetulus* populations. *Marine Ecology Progress Series* **306**, 269–281.

Bruce, B. D., Harasti, D., Lee, K., Gallen, C. & Bradford, R. (2019). Broad-scale movements of juvenile white sharks *Carcharodon carcharias* in eastern Australia from acoustic and satellite telemetry. *Marine Ecology Progress Series* **619**, 1–15.

Brule, T., Mena-Loria, A., Perez-Diaz, E. & Renan, X. (2011). Diet of juvenle gag *Mycteroperca microlepis* from a non-estuarine seagrass bed habitat in the southern Gulf of Mexico. *Bulletin of Marine Science* **87**(1), 31–43.

Burke, J. S., Kenworthy, W. J. & Wood, L. L. (2009). Ontogenetic patterns of concentration indicate lagoon nurseries are essential to common grunts stocks in a Puerto Rican bay. *Estuarine Coastal and Shelf Science* **81**(4), 533–543.

Burke, J. S., Miller, J. M. & Hoss, D. E. (1991). Immigration and settlement pattern of *Paralichthys dentatus* and *P. lethostigma* in an estuarine nursery ground, North Carolina, USA. *Netherlands Journal of Sea Research* **27**(3–4), 393–405.

Burns, N. M., Hopkins, C. R., Bailey, D. M. & Wright, P. J. (2020). Otolith chemoscape analysis in whiting links fishing grounds to nursery areas. *Communications Biology* **3**(1), 690.

Burnsed, S. W., Lowerre-Barbieri, S., Bickford, J. & Leone, E. H. (2020). Recruitment and movement ecology of red drum *Sciaenops ocellatus* differs by natal estuary. *Marine Ecology Progress Series* **633**, 181–196.

Bussotti, S., Guidetti, P. & Belmonte, G. (2003). Distribution patterns of the cardinal fish, *Apogon imberbis*, in shallow marine caves in southern Apulia (SE Italy). *Italian Journal of Zoology* **70**(2), 153–157.

Cabral, H. (2003). Differences in growth rates of juvenile *Solea solea* and *Solea senegalensis* in the Tagus estuary, Portugal. *Journal of the Marine Biological Association of the United Kingdom* **83**(4), 861–868.

Cabral, H. N. (2000). Comparative feeding ecology of sympatric *Solea solea* and *S. senegalensis*, within the nursery areas of the Tagus estuary, Portugal. *Journal of Fish Biology* **57**(6), 1550–1562.

Cabral, H. N., Vasconcelos, R., Vinagre, C., Franca, S., Fonseca, V., Maia, A., Reis-Santos, P., Lopes, M., Ruano, M., Campos, J., Freitas, V., Santos, P. T. & Costa, M. J. (2007). Relative importance of estuarine flatfish nurseries along the Portuguese coast. *Journal of Sea Research* **57**(2–3), 209–217.

Cantafaro, A., Ardizzone, G., Enea, M., Ligas, A. & Colloca, F. (2017). Assessing the importance of nursery areas of European hake (*Merluccius merluccius*) using a body condition index. *Ecological Indicators* **81**, 383–389.

Carbines, G., Jiang, W. M. & Beentjes, M. P. (2004). The impact of oyster dredging on the growth of blue cod, *Parapercis colias*, in Foveaux Strait, New Zealand. *Aquatic Conservation-Marine and Freshwater Ecosystems* **14**(5), 491–504.

Carl, J. D., Sparrevohn, C. R., Nicolajsen, H. & Stottrup, J. G. (2008). Substratum selection by juvenile flounder *Platichthys flesus* (L.): effect of ephemeral filamentous macroalgae. *Journal of Fish Biology* **72**(10), 2570–2578.

Carlisle, A. B., Litvin, S. Y., Hazen, E. L., Madigan, D. J., Goldman, K. J., Lea, R. N. & Block, B. A. (2015). Reconstructing habitat use by juvenile salmon sharks links upwelling to strandings in the California Current. *Marine Ecology Progress Series* **525**, 217–228.

Carlson, J. K., Heupel, M. R., Bethea, D. M. & Hollensead, L. D. (2008). Coastal habitat use and residency of juvenile Atlantic sharpnose sharks (*Rhizoprionodon terraenovae*). *Estuaries and Coasts* **31**(5), 931–940.

Carr, M. H. (1994). Effects of macroalgal dynamics on recruitment of a temperate reef fish. *Ecology* **75**(5), 1320–1333.

Carroll, J. M., Furman, B. T., Tettelbach, S. T. & Peterson, B. J. (2012). Balancing the edge effects budget: bay scallop settlement and loss along a seagrass edge. *Ecology* **93**(7), 1637–1647.

Cartamil, D., Wegner, N. C., Kacev, D., Ben-aderet, N., Kohin, S. & Graham, J. B. (2010). Movement patterns and nursery habitat of juvenile thresher sharks *Alopias vulpinus* in the Southern California Bight. *Marine Ecology Progress Series* **404**, 249–258.

Cerutti-Pereyra, F., Thums, M., Austin, C. M., Bradshaw, C. J. A., Stevens, J. D., Babcock, R. C., Pillans, R. D. & Meekan, M. G. (2014). Restricted movements of juvenile rays in the lagoon of Ningaloo Reef, Western Australia – evidence for the existence of a nursery. *Environmental Biology of Fishes* **97**(4), 371–383.

Cheminee, A., Francour, P. & Harmelin-Vivien, M. (2011). Assessment of *Diplodus* spp. (Sparidae) nursery grounds along the rocky shore of Marseilles (France, NW Mediterranean). *Scientia Marina* **75**(1), 181–188.

Cheminee, A., Merigot, B., Vanderklift, M. A. & Francour, P. (2016). Does habitat complexity influence fish recruitment? *Mediterranean Marine Science* **17**(1), 39–46.

Cheminee, A., Rider, M., Lenfant, P., Zawadzki, A., Merciere, A., Crec'hriou, R., Mercader, M., Saragoni, G., Neveu, R., Ternon, Q. & Pastor, J. (2017). Shallow rocky nursery habitat for fish: Spatial variability of juvenile fishes among this poorly protected essential habitat. *Marine Pollution Bulletin* **119**(1), 245–254.

Cheminee, A., Sala, E., Pastor, J., Bodilis, P., Thiriet, P., Mangialajo, L., Cottalorda, J. M. & Francour, P. (2013). Nursery value of Cystoseira forests for Mediterranean rocky reef fishes. *Journal of Experimental Marine Biology and Ecology* **442**, 70–79.

Chin, B. S., Nakagawa, M., Noda, T., Wada, T. & Yamashita, Y. (2013). Determining optimal release habitat for black rockfish, *Sebastes schlegelii*: Examining growth rate, feeding condition, and return rate. *Reviews in Fisheries Science* **21**(3–4), 286–298.

Chittaro, P. M., Finley, R. J. & Levin, P. S. (2009). Spatial and temporal patterns in the contribution of fish from their nursery habitats. *Oecologia* **160**(1), 49–61.

Chittaro, P. M., Usseglio, P. & Sale, P. (2005). Variation in fish density, assemblage composition and relative rates of predation among mangrove, seagrass and coral reef habitats. *Environmental Biology of Fishes* **72**(2), 175–187.

Chua, C. Y. Y. & Chou, L. M. (1994). The use of artificial reefs in enhancing fish communities in Singapore. *Hydrobiologia* **285**(1–3), 177–187.

Cianciotto, A. C., Shenker, J. M., Adams, A. J., Rennert, J. J. & Heuberger, D. (2019). Modifying mosquito impoundment management to enhance nursery habitat value for juvenile common Snook (*Centropomus undecimalis*) and Atlantic tarpon (*Megalops atlanticus*). *Environmental Biology of Fishes* **102**(2), 403–416.

Cigliano, J. A. & Kliman, R. M. (2014). Density, age structure, and length of queen conch (*Strombus gigas*) in shallow-water aggregations in the Sapodilla Cayes Marine Reserve, Belize. *Caribbean Journal of Science* **48**(1), 18–30.

Ciotti, B. J., Targett, T. E. & Burrows, M. T. (2013*a*). Decline in growth rate of juvenile European plaice (*Pleuronectes platessa*) during summer at nursery beaches along the west coast of Scotland. *Canadian Journal of Fisheries and Aquatic Sciences* **70**(5), 720–734.

Ciotti, B. J., Targett, T. E. & Burrows, M. T. (2013*b*). Spatial variation in growth rate of early juvenile European plaice *Pleuronectes platessa*. *Marine Ecology Progress Series* **475**, 213–232.

Ciotti, B. J., Targett, T. E., Nash, R. D. M. & Burrows, M. T. (2013*c*). Small-scale spatial and temporal heterogeneity in growth and condition of juvenile fish on sandy beaches. *Journal of Experimental Marine Biology and Ecology* **448**, 346–359.

Collatos, C., Abel, D. C. & Martin, K. L. (2020). Seasonal occurrence, relative abundance, and migratory movements of juvenile sandbar sharks, *Carcharhinus plumbeus*, in Winyah Bay, South Carolina. *Environmental Biology of Fishes* **103**(7), 859–873.

Colloca, F., Bartolino, V., Lasinio, G. J., Maiorano, L., Sartor, P. & Ardizzone, G. (2009). Identifying fish nurseries using density and persistence measures. *Marine Ecology Progress Series* **381**, 287–296.

Colloca, F., Cardinale, M. & Ardizzone, G. D. (1997). Biology, spatial distribution and population dynamics of *Lepidotrigla cavillone* (Pisces: Triglidae) in the central Tyrrhenian Sea. *Fisheries Research* **32**(1), 21–32.

Colloca, F., Garofalo, G., Bitetto, I., Facchini, M. T., Grati, F., Martiradonna, A., Mastrantonio, G., Nikolioudakis, N., Ordinas, F., Scarcella, G., Tserpes, G., Tugores, M. P., Valavanis, V., Carlucci, R., Fiorentino, F., *et al*. (2015). The seascape of demersal fish nursery areas in the North Mediterranean Sea, a first step towards the implementation of spatial planning for trawl fisheries. *PLoS One* **10**(3), e0119590.

Collocott, S. J., Vivier, L. & Cyrus, D. P. (2014). Prawn community structure in the subtropical Mfolozi-Msunduzi estuarine system, KwaZulu-Natal, South Africa. *African Journal of Aquatic Science* **39**(2), 127–140.

Colombano, D. D., Manfree, A. D., O'Rear, T. A., Durand, J. R. & Moyle, P. B. (2020). Estuarine-terrestrial habitat gradients enhance nursery function for resident and transient fishes in the San Francisco Estuary. *Marine Ecology Progress Series* **637**, 141–157.

Conrath, C. L. & Musick, J. A. (2007). The sandbar shark summer nursery within bays and lagoons of the Eastern Shore of Virginia. *Transactions of the American Fisheries Society* **136**(4), 999–1007.

Conrath, C. L. & Musick, J. A. (2008). Investigations into depth and temperature habitat utilization and overwintering grounds of juvenile sandbar sharks, *Carcharhinus plumbeus*: the importance of near shore North Carolina waters. *Environmental Biology of Fishes* **82**(2), 123–131.

Conrath, C. L. & Musick, J. A. (2010). Residency, space use and movement patterns of juvenile sandbar sharks (*Carcharhinus plumbeus*) within a Virginia summer nursery area. *Marine and Freshwater Research* **61**(2), 223–235.

Conroy, C. W., Piccoli, P. M. & Secor, D. H. (2015). Carryover effects of early growth and river flow on partial migration in striped bass *Morone saxatilis*. *Marine Ecology Progress Series* **541**, 179–194.

Cooper, D. W., Duffy-Anderson, J. T., Norcross, B. L., Holladay, B. A. & Stabeno, P. J. (2014). Nursery areas of juvenile northern rock sole (*Lepidopsetta polyxystra*) in the eastern Bering Sea in relation to hydrography and thermal regimes. *ICES Journal of Marine Science* **71**(7), 1683–1695.

Copeman, L., Ryer, C., Spencer, M., Ottmar, M., Iseri, P., Sremba, A., Wells, J. & Parrish, C. (2018). Benthic enrichment by diatom-sourced lipid promotes growth and condition in juvenile Tanner crabs around Kodiak Island, Alaska. *Marine Ecology Progress Series* **597**, 161–178.

Correa, B. & Vianna, M. (2016). Spatial and temporal distribution patterns of the silver mojarra *Eucinostomus argenteus* (Perciformes: Gerreidae) in a tropical semi-enclosed bay. *Journal of Fish Biology* **89**(1), 641–660.

Cortes, F., Jaureguizar, A. J., Menni, R. C. & Guerrero, R. A. (2011). Ontogenetic habitat preferences of the narrownose smooth-hound shark, *Mustelus schmitti*, in two Southwestern Atlantic coastal areas. *Hydrobiologia* **661**(1), 445–456.

Costa, M. D. P., Muelbert, J. H., Moraes, L. E., Vieira, J. P. & Castello, J. P. (2014). Estuarine early life stage habitat occupancy patterns of whitemouth croaker *Micropogonias furnieri* (Desmarest, 1830) from the Patos Lagoon, Brazil. *Fisheries Research* **160**, 77–84.

Costa, T. L. A., Thayer, J. A. & Mendes, L. F. (2015). Population characteristics, habitat and diet of a recently discovered stingray *Dasyatis marianae*: implications for conservation. *Journal of Fish Biology* **86**(2), 527–543.

Costalago, D., Kisten, Y., Clemmesen, C. & Strydom, N. A. (2020). Growth and nutritional condition of anchovy larvae on the west and southeast coasts of South Africa. *Marine Ecology Progress Series* **644**, 119–128.

Cote, D., Scruton, D. A., Niezgoda, G. H., McKinley, R. S., Rowsell, D. F., Lindstrom, R. T., Ollerhead, L. M. N. & Whitt, C. J. (1998). A coded acoustic telemetry system for high precision monitoring of fish location and movement: Application to the study of nearshore nursery habitat of juvenile Atlantic cod (*Gadus morhua*). *Marine Technology Society Journal* **32**(1), 54–62.

Cowan, D. F. (1999). Method for assessing relative abundance, size distribution, and growth of recently settled and early juvenile lobsters (*Homarus americanus*) in the lower intertidal, zone. *Journal of Crustacean Biology* **19**(4), 738–751.

Cowley, P. D., Kerwath, S. E., Childs, A. R., Thorstad, E. B., Okland, F. & Naesje, T. F. (2008). Estuarine habitat use by juvenile dusky kob *Argyrosomus japonicus* (Sciaenidae), with implications for management. *African Journal of Marine Science* **30**(2), 247–253.

Crawley, K. R., Hyndes, G. A. & Ayvazian, S. G. (2006). Influence of different volumes and types of detached macrophytes on fish community structure in surf zones of sandy beaches. *Marine Ecology Progress Series* **307**, 233–246.

Crear, D. P., Latour, R. J., Friedrichs, M. A. M., St-Laurent, P. & Weng, K. C. (2020). Sensitivity of a shark nursery habitat to a changing climate. *Marine Ecology Progress Series* **652**, 123–136.

Criscoli, A., Carpentieri, P., Colloca, F., Belluscio, A. & Ardizzone, G. (2017). Identification and characterization of nursery areas of red mullet *Mullus barbatus* in the Central Tyrrhenian Sea. *Marine and Coastal Fisheries* **9**(1), 203–215.

Crona, B. I. & Ronnback, P. (2005). Use of replanted mangroves as nursery grounds by shrimp communities in Gazi Bay, Kenya. *Estuarine Coastal and Shelf Science* **65**(3), 535–544.

Cuadros, A., Joan, M. B., Luis, C. C., Thiriet, P., Pastor, J., Arroyo, N. L. & Cheminee, A. (2017). Seascape attributes, at different spatial scales, determine settlement and post-settlement of juvenile fish. *Estuarine Coastal and Shelf Science* **185**, 120–129.

Cuadros, A., Moranta, J., Cardona, L., Thiriet, P., Francour, P., Vidal, E., Sintes, J. & Cheminee, A. (2019). Juvenile fish in Cystoseira forests: influence of habitat complexity and depth on fish behaviour and assemblage composition. *Mediterranean Marine Science* **20**(2), 380–392.

Cuevas-Gomez, G. A., Perez-Jimenez, J. C., Mendez-Loeza, I., Carrera-Fernandez, M. & Castillo-Geniz, J. L. (2020). Identification of a nursery area for the critically endangered hammerhead shark (*Sphyrna lewini*) amid intense fisheries in the southern Gulf of Mexico. *Journal of Fish Biology* **97**(4), 1087–1096.

Cure, K., Hobbs, J. P. A. & Harvey, E. S. (2015). High recruitment associated with increased sea temperatures towards the southern range edge of a Western Australian endemic reef fish *Choerodon rubescens* (family Labridae). *Environmental Biology of Fishes* **98**(4), 1059–1067.

Curran, M. C. & Able, K. W. (2002). Annual stability in the use of coves near inlets as settlement areas for winter flounder (*Pseudopleuronectes americanus*). *Estuaries* **25**(2), 227–234.

Curran, M. C. & Wilber, D. H. (2019). Seasonal and interannual variability in flatfish assemblages in a southeastern USA estuary. *Estuaries and Coasts* **42**(5), 1374–1386.

Curtis, T. H., Adams, D. H. & Burgess, G. H. (2011). Seasonal distribution and habitat associations of bull sharks in the Indian River Lagoon, Florida: A 30-year synthesis. *Transactions of the American Fisheries Society* **140**(5), 1213–1226.

Curtis, T. H., Metzger, G., Fischer, C., McBride, B., McCallister, M., Winn, L. J., Quinlan, J. & Ajemian, M. J. (2018). First insights into the movements of young-of-the-year white sharks (*Carcharodon carcharias*) in the western North Atlantic Ocean. *Scientific Reports* **8**, 10794.

Curtis, T. H., Parkyn, D. C. & Burgess, G. H. (2013). Use of human-altered habitats by bull sharks in a Florida nursery area. *Marine and Coastal Fisheries* **5**(1), 28–38.

D'Onghia, G., Maiorano, P., Sion, L., Giove, A., Capezzuto, F., Carlucci, R. & Tursi, A. (2010). Effects of deep-water coral banks on the abundance and size structure of the megafauna in the Mediterranean Sea. *Deep-Sea Research Part II-Topical Studies in Oceanography* **57**(5–6), 397–411.

da Silva, A. R., Barioto, J. G., Grabowski, R. C. & Castilho, A. L. (2017). Temporal and bathymetric distribution of juveniles and adults of the speckled swimming crab *Arenaeus cribrarius* from coastal waters of southern Brazil. *Biologia* **72**(3), 325–332.

Dahlgren, C. P. & Eggleston, D. B. (2001). Spatio-temporal variability in abundance, size and microhabitat associations of early juvenile Nassau grouper *Epinephelus striatus* in an off-reef nursery system. *Marine Ecology Progress Series* **217**, 145–156.

Daly, B. & Konar, B. (2008). Effects of macroalgal structural complexity on nearshore larval and post-larval crab composition. *Marine Biology* **153**(6), 1055–1064.

Dance, M. A. & Rooker, J. R. (2015). Habitat- and bay-scale connectivity of sympatric fishes in an estuarine nursery. *Estuarine Coastal and Shelf Science* **167**, 447–457.

Dance, M. A. & Rooker, J. R. (2016). Stage-specific variability in habitat associations of juvenile red drum across a latitudinal gradient. *Marine Ecology Progress Series* **557**, 221–235.

Dantas, D. V., Barletta, M., Lima, A. R. A., Ramos, J. D. A., da Costa, M. F. & Saint-Paul, U. (2012). Nursery habitat shifts in an estuarine ecosystem: patterns of use by sympatric catfish species. *Estuaries and Coasts* **35**(2), 587–602.

Davy, L. E., Simpfendorfer, C. A. & Heupel, M. R. (2015). Movement patterns and habitat use of juvenile mangrove whiprays (*Himantura granulata*). *Marine and Freshwater Research* **66**(6), 481–492.

Day, E. & Branch, G. M. (2002). Effects of sea urchins (*Parechinus angulosus*) on recruits and juveniles of abalone (*Haliotis midae*). *Ecological Monographs* **72**(1), 133–149.

Day, L., Brind'Amour, A., Cresson, P., Chouquet, B. & Le Bris, H. (2021). Contribution of estuarine and coastal habitats within nursery to the diets of juvenile fish in spring and autumn. *Estuaries and Coasts* **44**(4), 1100–1117.

Day, L., Le Bris, H., Saulnier, E., Pinsivy, L. & Brind'Amour, A. (2020). Benthic prey production index estimated from trawl survey supports the food limitation hypothesis in coastal fish nurseries. *Estuarine Coastal and Shelf Science* **235**, 106594.

de Araujo, A. L. F., Dantas, R. P. & Pessanha, A. L. M. (2016). Feeding ecology of three juvenile mojarras (Gerreidae) in a tropical estuary of northeastern Brazil. *Neotropical Ichthyology* **14**(1), e150039.

de Carvalho-Souza, G. F., Gonzalez-Ortegon, E., Baldeo, F., Vilas, C., Drake, P. & Llope, M. (2019). Natural and anthropogenic effects on the early life stages of European anchovy in one of its essential fish habitats, the Guadalquivir estuary. *Marine Ecology Progress Series* **617**, 67–79.

de Freitas, R. H. A., Rosa, R. S., Wetherbee, B. M. & Gruber, S. H. (2009). Population size and survivorship for juvenile lemon sharks (*Negaprion brevirostris*) on their nursery grounds at a marine protected area in Brazil. *Neotropical Ichthyology* **7**(2), 205–212.

de la Moriniere, E. C., Pollux, B. J. A., Nagelkerken, I. & van der Velde, G. (2002). Post-settlement life cycle migration patterns and habitat preference of coral reef fish that use seagrass and mangrove habitats as nurseries. *Estuarine Coastal and Shelf Science* **55**(2), 309–321.

de la Moriniere, E. C., Pollux, B. J. A., Nagelkerken, I. & van der Velde, G. (2003). Diet shifts of Caribbean grunts (Haemulidae) and snappers (Lutjanidae) and the relation with nursery-to-coral reef migrations. *Estuarine Coastal and Shelf Science* **57**(5–6), 1079–1089.

De Raedemaecker, F., Brophy, D., O'Connor, I. & Comerford, S. (2012*a*). Habitat characteristics promoting high density and condition of juvenile flatfish at nursery grounds on the west coast of Ireland. *Journal of Sea Research* **73**, 7–17.

De Raedemaecker, F., Brophy, D., O'Connor, I. & O'Neill, B. (2012*b*). Dependence of RNA:DNA ratios and Fulton's K condition indices on environmental characteristics of plaice and dab nursery grounds. *Estuarine Coastal and Shelf Science* **98**, 60–70.

De Raedemaecker, F., Keating, J., Brophy, D., O'Connor, I. & Mc Grath, D. (2011*a*). Spatial variability in diet, condition and growth of juvenile plaice (*Pleuronectes platessa*) at sandy beach nursery grounds on the south-west coast of Ireland. *Journal of the Marine Biological Association of the United Kingdom* **91**(6), 1215–1223.

De Raedemaecker, F., O'Connor, I., Brophy, D. & Black, A. (2011*b*). Macrobenthic prey availability and the potential for food competition between 0 year group *Pleuronectes platessa* and *Limanda limanda*. *Journal of Fish Biology* **79**(7), 1918–1939.

Debarros, P. & Holst, J. C. (1995). Identification of geographic origin of Norwegian spring-spawning herring (*Clupea harengus* L) based on measurements of scale annuli. *ICES Journal of Marine Science* **52**(5), 863–872.

Delany, J., Myers, A. A. & McGrath, D. (1998). Recruitment, immigration and population structure of two coexisting limpet species in mid-shore tidepools, on the West Coast of Ireland. *Journal of Experimental Marine Biology and Ecology* **221**(2), 221–230.

Dias, M., Silva, A., Cabral, H. N. & Vinagre, C. (2014). Diet of marine fish larvae and juveniles that use rocky intertidal pools at the Portuguese coast. *Journal of Applied Ichthyology* **30**(5), 970–977.

Diaz-Gil, C., Smee, S. L., Cotgrove, L., Follana-Berna, G., Hinz, H., Marti-Puig, P., Grau, A., Palmer, M. & Catalan, I. A. (2017). Using stereoscopic video cameras to evaluate seagrass meadows nursery function in the Mediterranean. *Marine Biology* **164**(6), 137.

Diaz, R. J., Cutter, G. R. & Able, K. W. (2003). The importance of physical and biogenic structure to juvenile fishes on the shallow inner continental shelf. *Estuaries* **26**(1), 12–20.

Dinning, K. M. & Rochette, R. (2019). Evidence that mud seafloor serves as recruitment habitat for settling and early benthic phase of the American lobster *Homarus americanus* H. (Decapoda: Astacidea: Nephropidae). *Journal of Crustacean Biology* **39**(5), 594–601.

Dittel, A. I., Epifanio, C. E. & Fogel, M. L. (2006). Trophic relationships of juvenile blue crabs (*Callinectes sapidus*) in estuarine habitats. *Hydrobiologia* **568**, 379–390.

Dolbeth, M., Martinho, F., Viegas, I., Cabral, H. & Pardal, M. A. (2008). Estuarine production of resident and nursery fish species: Conditioning by drought events? *Estuarine Coastal and Shelf Science* **78**(1), 51–60.

Dorel, D., Koutsikopoulos, C., Desaunay, Y. & Marchand, J. (1991). Seasonal distribution of young sole (*Solea solea* (L)) in the nursery ground of the Bay of Vilaine (Northern Bay of Biscay). *Netherlands Journal of Sea Research* **27**(3–4), 297–306.

Dorenbosch, M., Grol, M. G. G., Nagelkerken, I. & van der Velde, G. (2006). Seagrass beds and mangroves as potential nurseries for the threatened Indo-Pacific humphead wrasse, *Cheilinus undulatus* and Caribbean rainbow parrotfish, *Scarus quacamaia*. *Biological Conservation* **129**(2), 277–282.

Dorenbosch, M., Verweij, M. C., Nagelkerken, I., Jiddawi, N. & van der Velde, G. (2004). Homing and daytime tidal movements of juvenile snappers (Lutjanidae) between shallow-water nursery habitats in Zanzibar, western Indian Ocean. *Environmental Biology of Fishes* **70**(3), 203–209.

Dorf, B. A. & Powell, J. C. (1997). Distribution, abundance, and habitat characteristics of juvenile tautog (*Tautoga onitis*, Family Labridae) in Narragansett Bay, Rhode Island, 1988–1992. *Estuaries* **20**(3), 589–600.

Drymon, J. M., Ajemian, M. J. & Powers, S. P. (2014). Distribution and dynamic habitat use of young bull sharks *Carcharhinus leucas* in a highly stratified northern Gulf of Mexico estuary. *PLoS One* **9**(5), e97124.

Dufour, V., Cantou, M. & Lecomte, F. (2009). Identification of sea bass (*Dicentrarchus labrax*) nursery areas in the north-western Mediterranean Sea. *Journal of the Marine Biological Association of the United Kingdom* **89**(7), 1367–1374.

Dumbauld, B. R., Murphy, J. R., McCoy, L. & Lewis, N. S. (2021). A comparison of the juvenile dungeness crab *Metacarcinus magister* habitat provided by contemporary oyster aquaculture versus historical native oysters in a US West Coast estuary. *Journal of Shellfish Research* **40**(1), 161–175.

Durieux, E. D. H., Begout, M. L., Pinet, P. & Sasal, P. (2010). Digenean metacercariae parasites as natural tags of habitat use by 0-group common sole *Solea solea* in nearshore coastal areas: A case study in the embayed system of the Pertuis Charentais (Bay of Biscay, France). *Journal of Sea Research* **64**(1–2), 107–117.

Dwyer, R. G., Campbell, H. A., Cramp, R. L., Burke, C. L., Micheli-Campbell, M. A., Pillans, R. D., Lyon, B. J. & Franklin, C. E. (2020). Niche partitioning between river shark species is driven by seasonal fluctuations in environmental salinity. *Functional Ecology* **34**(10), 2170–2185.

Eby, L. A., Crowder, L. B., McClellan, C. M., Peterson, C. H. & Powers, M. J. (2005). Habitat degradation from intermittent hypoxia: impacts on demersal fishes. *Marine Ecology Progress Series* **291**, 249–261.

Eggertsen, M., Chacin, D. H., van Lier, J., Eggertsen, L., Fulton, C. J., Wilson, S., Halling, C. & Berkstrom, C. (2020). Seascape configuration and fine-scale habitat complexity shape parrotfish distribution and function across a coral reef lagoon. *Diversity* **12**(10), 391.

Eggleston, D. B. (1995). Recruitment in Nassau grouper *Epinephelus striatus* – postsettlement abundance, microhabitat features, and ontogenic habitat shifts. *Marine Ecology Progress Series* **124**(1–3), 9–22.

Eggleston, D. B. & Dahlgren, C. P. (2001). Distribution and abundance of Caribbean spiny lobsters in the Key West National Wildlife Refuge: relationship to habitat features and impact of an intensive recreational fishery. *Marine and Freshwater Research* **52**(8), 1567–1576.

Eggleston, D. B., Etherington, L. L. & Elis, W. E. (1998). Organism response to habitat patchiness: species and habitat-dependent recruitment of decapod crustaceans. *Journal of Experimental Marine Biology and Ecology* **223**(1), 111–132.

Eggleston, D. B. & Lipcius, R. N. (1992). Shelter selection by spiny lobster under variable predation risk, social conditions, and shelter size. *Ecology* **73**(3), 992–1011.

Eggleston, D. B., Lipcius, R. N. & Miller, D. L. (1992). Artificial shelters and survival of juvenile caribbean spiny lobster *Panulirus argus* – spatial, habitat, and lobster size effects. *Fishery Bulletin* **90**(4), 691–702.

Ehresmann, R. K., Beaudreau, A. H. & Green, K. M. (2018). Movement patterns of juvenile sablefish within a nursery area in Southeast Alaska. *Transactions of the American Fisheries Society* **147**(6), 1052–1066.

Elliott, S. A. M., Turrell, W. R., Heath, M. R. & Bailey, D. M. (2017). Juvenile gadoid habitat and ontogenetic shift observations using stereo-video baited cameras. *Marine Ecology Progress Series* **568**, 123–135.

Ellis, J. K. & Musick, J. A. (2007). Ontogenetic changes in the diet of the sandbar shark, *Carcharhinus plumbeus*, in lower Chesapeake Bay and Virginia (USA) coastal waters. *Environmental Biology of Fishes* **80**(1), 51–67.

Ellis, W. L. & Bell, S. S. (2004). Conditional use of mangrove habitats by fishes: Depth as a cue to avoid predators. *Estuaries* **27**(6), 966–976.

Elston, C., Cowley, P. D., von Brandis, R. G. & Lea, J. (2021). Residency and habitat use patterns by sympatric stingrays at a remote atoll in the Western Indian Ocean. *Marine Ecology Progress Series* **662**, 97–114.

Epifanio, C. E., Dittel, A. I., Rodriguez, R. A. & Targett, T. E. (2003). The role of macroalgal beds as nursery habitat for juvenile blue crabs, *Callinectes sapidus*. *Journal of Shellfish Research* **22**(3), 881–886.

Escalas, A., Ferraton, F., Paillon, C., Vidy, G., Carcaillet, F., Salen-Picard, C., Le Loc'h, F., Richard, P. & Darnaude, A. M. (2015). Spatial variations in dietary organic matter sources modulate the size and condition of fish juveniles in temperate lagoon nursery sites. *Estuarine Coastal and Shelf Science* **152**, 78–90.

Espino, F., Gonzalez, J. A., Haroun, R. & Tuya, F. (2015). Abundance and biomass of the parrotfish *Sparisoma cretense* in seagrass meadows: temporal and spatial differences between seagrass interiors and seagrass adjacent to reefs. *Environmental Biology of Fishes* **98**(1), 121–133.

Etherington, L. L. & Eggleston, D. B. (2003). Spatial dynamics of large-scale, multistage crab (*Callinectes sapidus*) dispersal: determinants and consequences for recruitment. *Canadian Journal of Fisheries and Aquatic Sciences* **60**(7), 873–887.

Everett, B. I., Cliff, G., Dudley, S. F. J., Wintner, S. P. & van der Elst, R. P. (2015). Do sawfish *Pristis* spp. represent South Africa's first local extirpation of marine elasmobranchs in the modern era? *African Journal of Marine Science* **37**(2), 275–284.

Fairchild, E. A., Sulikowski, J., Rennels, N., Howell, W. H. & Gurshin, C. W. D. (2008). Distribution of winter flounder, *Pseudopleuronectes americanus*, in the Hampton-Seabrook Estuary, New Hampshire: Observations from a field study. *Estuaries and Coasts* **31**(6), 1158–1173.

Fairclough, D. V. (2016). Similar cryptic behaviour during the early juvenile phase of two unrelated reef fishes: *Epinephelides armatus* and *Bodianus frenchii*. *Marine and Freshwater Behaviour and Physiology* **49**(2), 109–117.

Fairclough, D. V., Edmonds, J. S., Lenanton, R. C. J., Jackson, G., Keay, I. S., Crisafulli, B. M. & Newman, S. J. (2011). Rapid and cost-effective assessment of connectivity among assemblages of *Choerodon rubescens* (Labridae), using laser ablation ICP-MS of sagittal otoliths. *Journal of Experimental Marine Biology and Ecology* **403**(1–2), 46–53.

Farrugia, T. J., Espinoza, M. & Lowe, C. G. (2011). Abundance, habitat use and movement patterns of the shovelnose guitarfish (*Rhinobatos productus*) in a restored southern California estuary. *Marine and Freshwater Research* **62**(6), 648–657.

Faunce, C. H. & Serafy, J. E. (2007). Nearshore habitat use by gray snapper (*Lutjanus griseus*) and bluestriped grunt (*Haemulon sciurus*): environmental gradients and ontogenetic shifts. *Bulletin of Marine Science* **80**(3), 473–495.

Faunce, C. H. & Serafy, J. E. (2008). Growth and secondary production of an eventual reef fish during mangrove residency. *Estuarine Coastal and Shelf Science* **79**(1), 93–100.

Fedewa, E. J., Miller, J. A., Hurst, T. P. & Jiang, D. (2017). The potential effects of pre-settlement processes on post-settlement growth and survival of juvenile northern rock sole (*Lepidopsetta polyxystra*) in Gulf of Alaska nursery habitats. *Estuarine Coastal and Shelf Science* **189**, 46–57.

Feldman, K. L., Armstrong, D. A., Eggleston, D. B. & Dumbauld, B. R. (1997). Effects of substrate selection and post-settlement survival on recruitment success of the thalassinidean shrimp *Neotrypaea californiensis* to intertidal shell and mud habitats. *Marine Ecology Progress Series* **150**(1–3), 121–136.

Fernandez-Delgado, C., Baldo, F., Vilas, C., Garcia-Gonzalez, D., Cuesta, J. A., Gonzalez-Ortegon, E. & Drake, P. (2007). Effects of the river discharge management on the nursery function of the Guadalquivir river estuary (SW Spain). *Hydrobiologia* **587**, 125–136.

Field, J. M. & Butler, M. J. (1994). The influence of temperature, salinity, and postlarval transport on the distribution of juvenile spiny lobsters, *Panulirus argus* (Latreille, 1804), in Florida Bay. *Crustaceana* **67**, 26–45.

Figueira, W. F., Booth, D. J. & Gregson, M. A. (2008). Selective mortality of a coral reef damselfish: role of predator-competitor synergisms. *Oecologia* **156**(1), 215–226.

Finn, M. D. & Kingsford, M. J. (1996). Two-phase recruitment of apogonids (Pisces) on the Great Barrier Reef. *Marine and Freshwater Research* **47**(2), 423–432.

Flaherty, K. E., Switzer, T. S., Winner, B. L. & Keenan, S. F. (2014). Regional correspondence in habitat occupancy by gray snapper (*Lutjanus griseus*) in estuaries of the Southeastern United States. *Estuaries and Coasts* **37**(1), 206–228.

Florin, A. B., Sundblad, G. & Bergstrom, U. (2009). Characterisation of juvenile flatfish habitats in the Baltic Sea. *Estuarine Coastal and Shelf Science* **82**(2), 294–300.

Fodrie, F. J., Heck, K. L., Andrus, C. F. T. & Powers, S. P. (2020). Determinants of the nursery role of seagrass meadows in the sub-tropical Gulf of Mexico: inshore-offshore connectivity for snapper and grouper. *Marine Ecology Progress Series* **647**, 135–147.

Fodrie, F. J. & Herzka, S. Z. (2008). Tracking juvenile fish movement and nursery contribution within and coastal embayments via otolith microchemistry. *Marine Ecology Progress Series* **361**, 253–265.

Fodrie, F. J. & Levin, L. A. (2008). Linking juvenile habitat utilization to population dynamics of California halibut. *Limnology and Oceanography* **53**(2), 799–812.

Fodrie, F. J., Levin, L. A. & Lucas, A. J. (2009). Use of population fitness to evaluate the nursery function of juvenile habitats. *Marine Ecology Progress Series* **385**, 39–49.

Fodrie, F. J. & Mendoza, G. (2006). Availability, usage and expected contribution of potential nursery habitats for the California halibut. *Estuarine Coastal and Shelf Science* **68**(1–2), 149–164.

Fonseca, V. F., Vasconcelos, R. P., Tanner, S. E., Franca, S., Serafim, A., Lopes, B., Company, R., Bebianno, M. J., Costa, M. J. & Cabral, H. N. (2015). Habitat quality of estuarine nursery grounds: Integrating non-biological indicators and multilevel biological responses in *Solea senegalensis*. *Ecological Indicators* **58**, 335–345.

Fonseca, V. F., Vinagre, C. & Cabral, H. N. (2006). Growth variability of juvenile soles *Solea solea* and *Solea senegalensis*, and comparison with RNA:DNA ratios in the Tagus Estuary, Portugal. *Journal of Fish Biology* **68**(5), 1551–1562.

Ford, J. R., Shima, J. S. & Swearer, S. E. (2016). Interactive effects of shelter and conspecific density shape mortality, growth, and condition in juvenile reef fish. *Ecology* **97**(6), 1373–1380.

Forrester, G. E. & Swearer, S. E. (2002). Trace elements in otoliths indicate the use of open-coast versus bay nursery habitats by juvenile California halibut. *Marine Ecology Progress Series* **241**, 201–213.

Fortibuoni, T., Bahri, T., Camilleri, M., Garofalo, G., Gristina, M. & Fiorentino, F. (2010). Nursery and spawning areas of deep-water rose shrimp, *Parapenaeus longirostris* (Decapoda: Penaeidae), in the Strait of Sicily (Central Mediterranean Sea). *Journal of Crustacean Biology* **30**(2), 167–174.

Fowler, A. J., Hamer, P. A. & Kemp, J. (2017). Age-related otolith chemistry profiles help resolve demographics and meta-population structure of a widely-dispersed, coastal fishery species. *Fisheries Research* **189**, 77–94.

Fowler, A. J. & Jennings, P. R. (2003). Dynamics in 0+ recruitment and early life history for snapper (*Pagrus auratus*, Sparidae) in South Australia. *Marine and Freshwater Research* **54**(8), 941–956.

Fox, A. G. & Peterson, D. L. (2019). Movement and out-migration of juvenile Atlantic sturgeon in Georgia, USA. *Transactions of the American Fisheries Society* **148**(5), 952–962.

Fox, C. J., Targett, T. E., Ciotti, B. J., de Kroon, K., Hortsmeyer, L. & Burrows, M. T. (2014). Size variation of 0-group plaice: Are earlier influences on growth potential a contributing factor? *Journal of Sea Research* **88**, 59–66.

Franca, S., Vinagre, C., Costa, M. J. & Cabral, H. N. (2004). Use of the coastal areas adjacent to the Douro estuary as a nursery area for pouting, *Trisopterus luscus* Linnaeus, 1758. *Journal of Applied Ichthyology* **20**(2), 99–104.

Francis, M. P. (2013). Temporal and spatial patterns of habitat use by juveniles of a small coastal shark (*Mustelus lenticulatus*) in an estuarine nursery. *PLoS One* **8**(2), e57021.

Franco, A., Fiorin, R., Zucchetta, M., Torricelli, P. & Franzoi, P. (2010). Flounder growth and production as indicators of the nursery value of marsh habitats in a Mediterranean lagoon. *Journal of Sea Research* **64**(4), 457–464.

Freire, J., Carabel, S., Verisimo, P., Bernardez, C. & Fernandez, L. (2009). Patterns of juvenile habitat use by the spider crab *Maja brachydactyla* as revealed by stable isotope analyses. *Scientia Marina* **73**(1), 39–49.

Freitas, V., Costa-Dias, S., Campos, J., Bio, A., Santos, P. & Antunes, C. (2009). Patterns in abundance and distribution of juvenile flounder, *Platichthys flesus*, in Minho estuary (NW Iberian Peninsula). *Aquatic Ecology* **43**(4), 1143–1153.

Freitas, V., Witte, J. I. J., Tulp, I. & van der Veer, H. W. (2016). Shifts in nursery habitat utilization by 0-group plaice in the western Dutch Wadden Sea. *Journal of Sea Research* **111**, 65–75.

Froeschke, B. F., Stunz, G. W., Robillard, M. M. R., Williams, J. & Froeschke, J. T. (2013). A modeling and field approach to identify Essential Fish Habitat for juvenile bay whiff (*Citharichthys spilopterus*) and southern flounder (*Paralichthys lethostigma*) within the Aransas Bay complex, TX. *Estuaries and Coasts* **36**(5), 881–892.

Froeschke, J. T., Stunz, G. W., Sterba-Boatwright, B. & Wildhaber, M. L. (2010). An empirical test of the 'shark nursery area concept' in Texas bays using a long-term fisheries-independent data set. *Aquatic Biology* **11**(1), 65–76.

Fry, B. (2008). Open bays as nurseries for Louisiana brown shrimp. *Estuaries and Coasts* **31**(4), 776–789.

Fry, B., Baltz, D. M., Benfield, M. C., Fleeger, J. W., Gace, A., Haas, H. L. & Quinones-Rivera, Z. J. (2003). Stable isotope indicators of movement and residency for brown shrimp (*Farfantepenaeus aztecus*) in coastal Louisiana marshscapes. *Estuaries* **26**(1), 82–97.

Fry, G., Milton, D. A., Van Der Velde, T., Stobutzki, I., Andamari, R., Badrudin & Sumiono, B. (2009). Reproductive dynamics and nursery habitat preferences of two commercially important Indo-Pacific red snappers *Lutjanus erythropterus* and *L. malabaricus*. *Fisheries Science* **75**(1), 145–158.

Fuji, T., Kasai, A., Suzuki, K. W., Ueno, M. & Yamashita, Y. (2011). Migration ecology of juvenile temperate seabass *Lateolabrax japonicus*: a carbon stable-isotope approach. *Journal of Fish Biology* **78**(7), 2010–2025.

Fuji, T., Kasai, A., Ueno, M. & Yamashita, Y. (2016*a*). Importance of estuarine nursery areas for the adult population of the temperate seabass *Lateolabrax japonicus*, as revealed by otolith Sr:Ca ratios. *Fisheries Oceanography* **25**(4), 448–456.

Fuji, T., Kasai, A., Ueno, M. & Yamashita, Y. (2016*b*). The importance of estuarine production of large prey for the gowth of juvenile temperate seabass (*Lateolabrax japonicus*). *Estuaries and Coasts* **39**(4), 1208–1220.

Fujioka, K., Fukuda, H., Furukawa, S., Tei, Y., Okamoto, S. & Ohshimo, S. (2018). Habitat use and movement patterns of small (age-0) juvenile Pacific bluefin tuna (*Thunnus orientalis*) relative to the Kuroshio. *Fisheries Oceanography* **27**(3), 185–198.

Furey, N. B. & Rooker, J. R. (2013). Spatial and temporal shifts in suitable habitat of juvenile southern flounder (*Paralichthys lethostigma*). *Journal of Sea Research* **76**, 161–169.

Gallagher, M. B. & Heppell, S. S. (2010). Essential habitat identification for age-0 rockfish along the central Oregon coast. *Marine and Coastal Fisheries* **2**(1), 60–72.

Garces, C., Niklitschek, E. J., Plaza, G., Cerna, F., Leisen, M., Toledo, P. & Barra, F. (2019). Anchoveta *Engraulis ringens* along the Chilean coast: Management units, demographic units and water masses: Insights from multiple otolith-based approaches. *Fisheries Oceanography* **28**(6), 735–750.

Garofalo, G., Ceriola, L., Gristina, M., Fiorentino, F. & Pace, R. (2010). Nurseries, spawning grounds and recruitment of *Octopus vulgaris* in the Strait of Sicily, central Mediterranean Sea. *ICES Journal of Marine Science* **67**(7), 1363–1371.

Garofalo, G., Fortibuoni, T., Gristina, M., Sinopoli, M. & Fiorentino, F. (2011). Persistence and co-occurrence of demersal nurseries in the Strait of Sicily (central Mediterranean): Implications for fishery management. *Journal of Sea Research* **66**(1), 29–38.

Garwood, J. A., Allen, D. M., Kimball, M. E. & Boswell, K. M. (2019). Site fidelity and habitat use by young-of-the-year transient fishes in salt marsh intertidal creeks. *Estuaries and Coasts* **42**(5), 1387–1396.

Geange, S. W. (2010). Effects of larger heterospecifics and structural refuge on the survival of a coral reef fish, *Thalassoma hardwicke*. *Marine Ecology Progress Series* **407**, 197–207.

Geary, B. W., Mikulas, J. J., Rooker, J. R., Landry, A. M. & Dellapenna, T. M. (2007). Patterns of habitat use by newly settled red snapper in the northwestern Gulf of Mexico. In W. F. Patterson, J. H. Cowan, G. R. Fitzhugh, & D. L. Nieland (Eds.), *Red Snapper Ecology and Fisheries in the U.S. Gulf of Mexico* (Vol. 60, pp. 25-38). Bethesda: Amer Fisheries Soc.

George, L. W., Martins, A. P. B., Heupel, M. R. & Simpfendorfer, C. A. (2019). Fine-scale movements of juvenile blacktip reef sharks *Carcharhinus melanopterus* in a shallow nearshore nursery. *Marine Ecology Progress Series* **623**, 85–97.

Gerard, T., Malca, E., Muhling, B. A., Mateo, I. & Lamkin, J. T. (2015). Isotopic signatures in the otoliths of reef-associated fishes of southern Florida: Linkages between nursery grounds and coral reefs. *Regional Studies in Marine Science* **2**, 95–104.

Gericke, R. L., Heck, K. L. & Fodrie, F. J. (2014). Interactions between northern-shifting tropical species and native species in the northern Gulf of Mexico. *Estuaries and Coasts* **37**(4), 952–961.

Giannoulaki, M., Iglesias, M., Tugores, M. P., Bonanno, A., Patti, B., De Felice, A., Leonori, I., Bigot, J. L., Ticina, V., Pyrounaki, M. M., Tsagarakis, K., Machias, A., Somarakis, S., Schismenou, E., Quinci, E., *et al*. (2013). Characterizing the potential habitat of European anchovy *Engraulis encrasicolus* in the Mediterranean Sea, at different life stages. *Fisheries Oceanography* **22**(2), 69–89.

Gibson, R. N., Pihl, L., Burrows, M. T., Modin, J., Wennhage, H. & Nickell, L. A. (1998). Diel movements of juvenile plaice *Pleuronectes platessa* in relation to predators, competitors, food availability and abiotic factors on a microtidal nursery ground. *Marine Ecology Progress Series* **165**, 145–159.

Gibson, R. N., Robb, L., Wennhage, H. & Burrows, M. T. (2002). Ontogenetic changes in depth distribution of juvenile flatfishes in relation to predation risk and temperature on a shallow-water nursery ground. *Marine Ecology Progress Series* **229**, 233–244.

Gillanders, B. M. (1997). Patterns of abundance and size structure in the blue groper, *Achoerodus viridis* (Pisces, Labridae): Evidence of links between estuaries and coastal reefs. *Environmental Biology of Fishes* **49**(2), 153–173.

Gillanders, B. M. (2002). Connectivity between juvenile and adult fish populations: do adults remain near their recruitment estuaries? *Marine Ecology Progress Series* **240**, 215–223.

Gillanders, B. M. & Kingsford, M. J. (1996). Elements in otoliths may elucidate the contribution of estuarine recruitment to sustaining coastal reef populations of a temperate reef fish. *Marine Ecology Progress Series* **141**(1–3), 13–20.

Gilliers, C., Amara, R., Bergeron, J. P. & Le Pape, O. (2004). Comparison of growth and condition indices of juvenile flatfish in different coastal nursery grounds. *Environmental Biology of Fishes* **71**(2), 189–198.

Gilliers, C., Le Pape, O., Desaunay, Y., Bergeron, J. P., Schreiber, N., Guerault, D. & Amara, R. (2006*a*). Growth and condition of juvenile sole (*Solea solea* L.) as indicators of habitat quality in coastal and estuarine nurseries in the Bay of Biscay with a focus on sites exposed to the Erika oil spill. *Scientia Marina* **70**, 183–192.

Gilliers, C., Le Pape, O., Desaunay, Y., Morin, J., Guerault, D. & Amara, R. (2006*b*). Are growth and density quantitative indicators of essential fish habitat quality? An application to the common sole *Solea solea* nursery grounds. *Estuarine Coastal and Shelf Science* **69**(1–2), 96–106.

Glass, L. A., Rooker, J. R., Kraus, R. T. & Holt, G. J. (2008). Distribution, condition, and growth of newly settled southern flounder (*Paralichthys lethostigma*) in the Galveston Bay Estuary, TX. *Journal of Sea Research* **59**(4), 259–268.

Glaus, K. B. J., Brunnschweiler, J. M., Piovano, S., Mescam, G., Genter, F., Fluekiger, P. & Rico, C. (2019). Essential waters: Young bull sharks in Fiji's largest riverine system. *Ecology and Evolution* **9**(13), 7574–7585.

Goldberg, R., Phelan, B., Pereira, J., Hagan, S., Clark, P., Bejda, A., Calabrese, A., Studholme, A. & Able, K. W. (2002). Variability in habitat use by young-of-the-year winter flounder, *Pseudopleuronectes americanus*, in three northeastern US estuaries. *Estuaries* **25**(2), 215–226.

Gonzalez-Ortegon, E., Subida, M. D., Cuesta, J. A., Arias, A. M., Fernandez-Delgado, C. & Drake, P. (2010). The impact of extreme turbidity events on the nursery function of a temperate European estuary with regulated freshwater inflow. *Estuarine Coastal and Shelf Science* **87**(2), 311–324.

Goode, K. L., Dunphy, B. J. & Parsons, D. M. (2020). Environmental metabolomics as an ecological indicator: Metabolite profiles in juvenile fish discriminate sites with different nursery habitat qualities. *Ecological Indicators* **115**, 106361.

Gorman, A. M., Gregory, R. S. & Schneider, D. C. (2009). Eelgrass patch size and proximity to the patch edge affect predation risk of recently settled age 0 cod (*Gadus*). *Journal of Experimental Marine Biology and Ecology* **371**(1), 1–9.

Gosselin, L. A. & Chia, F. S. (1995). Distribution and dispersal of early juvenile snails: Effectiveness of intertidal microhabitats as refuges and food sources. *Marine Ecology Progress Series* **128**(1–3), 213–223.

Gotceitas, V., Fraser, S. & Brown, J. A. (1997). Use of eelgrass beds (*Zostera marina*) by juvenile Atlantic cod (*Gadus morhua*). *Canadian Journal of Fisheries and Aquatic Sciences* **54**(6), 1306–1319.

Granados-Dieseldorff, P. & Baltz, D. M. (2008). Habitat use by nekton along a stream-order gradient in a Louisiana estuary. *Estuaries and Coasts* **31**(3), 572–583.

Grant, S. M. & Brown, J. A. (1998). Nearshore settlement and localized populations of Atlantic cod (*Gadus morhua*) in shallow coastal waters of Newfoundland. *Canadian Journal of Fisheries and Aquatic Sciences* **55**(6), 1317–1327.

Gray, C. A., Haddy, J. A., Fearman, J., Barnes, L. M., Macbeth, W. G. & Kendall, B. W. (2012). Reproduction, growth and connectivity among populations of *Girella tricuspidata* (Pisces: Girellidae). *Aquatic Biology* **16**(1), 53–68.

Grecay, P. A. & Targett, T. E. (1996). Spatial patterns in condition and feeding of juvenile weakfish in Delaware Bay. *Transactions of the American Fisheries Society* **125**(5), 803–808.

Griffiths, M. H. (1997). Influence of prey availability on the distribution of dusky kob *Argyrosomus japonicus* (Sciaenidae) in the Great Fish River estuary, with notes on the diet of early juveniles from three other estuarine systems. *South African Journal of Marine Science* **18**, 137–145.

Grimes, T. M., Tinker, M. T., Hughes, B. B., Boyer, K. E., Needless, L., Beheshti, K. & Lewison, R. L. (2020). Characterizing the impact of recovering sea otters on commercially important crabs in California estuaries. *Marine Ecology Progress Series* **655**, 123–137.

Grol, M. G. G., Dorenbosch, M., Kokkelmans, E. M. G. & Nagelkerken, I. (2008). Mangroves and seagrass beds do not enhance growth of early juveniles of a coral reef fish. *Marine Ecology Progress Series* **366**, 137–146.

Grol, M. G. G., Nagelkerken, I., Bosch, N. & Meesters, E. H. (2011*a*). Preference of early juveniles of a coral reef fish for distinct lagoonal microhabitats is not related to common measures of structural complexity. *Marine Ecology Progress Series* **432**, 221–233.

Grol, M. G. G., Nagelkerken, I., Rypel, A. L. & Layman, C. A. (2011*b*). Simple ecological trade-offs give rise to emergent cross-ecosystem distributions of a coral reef fish. *Oecologia* **165**(1), 79–88.

Gruss, A., Pirtle, J. L., Thorson, J. T., Lindeberg, M. R., Neff, A. D., Lewis, S. G. & Essington, T. E. (2021). Modeling nearshore fish habitats using Alaska as a regional case study. *Fisheries Research* **238**, 105905.

Guidetti, P. & Boero, F. (2004). Desertification of Mediterranean rocky reefs caused by date-mussel, *Lithophaga lithophaga* (Mollusca : Bivalvia), fishery: effects on adult and juvenile abundance of a temperate fish. *Marine Pollution Bulletin* **48**(9–10), 978–982.

Guinand, B., Fustier, M. A., Labonne, M., Jourdain, E., Calves, I., Quiniou, L., Cerqueira, F. & Laroche, J. (2013). Genetic structure and heterozygosity-fitness correlation in young-of-the-year sole (*Solea solea* L.) inhabiting three contaminated West-European estuaries. *Journal of Sea Research* **80**, 35–49.

Guindon, K. Y. & Miller, J. M. (1995). Growth potential of juvenile southern flounder, *Paralichthys lethostigma*, in low salinity nursery areas of Pamlico Sound, North Carolina, USA. *Netherlands Journal of Sea Research* **34**(1–3), 89–100.

Gunnarsson, B., Jonasson, J. P. & McAdam, B. J. (2010). Variation in hatch date distributions, settlement and growth of juvenile plaice (*Pleuronectes platessa* L.) in Icelandic waters. *Journal of Sea Research* **64**(1–2), 61–67.

Guttridge, T. L., Gruber, S. H., Franks, B. R., Kessel, S. T., Gledhill, K. S., Uphill, J., Krause, J. & Sims, D. W. (2012). Deep danger: intra-specific predation risk influences habitat use and aggregation formation of juvenile lemon sharks *Negaprion brevirostris*. *Marine Ecology Progress Series* **445**, 279–291.

Gutzler, B. C., Butler, M. J. & Behringer, D. C. (2015). Casitas: a location-dependent ecological trap for juvenile Caribbean spiny lobsters, *Panulirus argus*. *ICES Journal of Marine Science* **72**, 177–184.

Haggarty, D. R., Lotterhos, K. E. & Shurin, J. B. (2017). Young-of-the-year recruitment does not predict the abundance of older age classes in black rockfish in Barkley Sound, British Columbia, Canada. *Marine Ecology Progress Series* **574**, 113–126.

Hale, E. A., Park, I. A., Fisher, M. T., Wong, R. A., Stangl, M. J. & Clark, J. H. (2016). Abundance estimate for and habitat use by early juvenile Atlantic sturgeon within the Delaware River Estuary. *Transactions of the American Fisheries Society* **145**(6), 1193–1201.

Halliday, I. A. (1995). Influence of natural fluctuations in seagrass cover on commercial prawn nursery grounds in a subtropical estuary. *Marine and Freshwater Research* **46**(8), 1121–1126.

Hamilton, R. J., Almany, G. R., Brown, C. J., Pita, J., Peterson, N. A. & Choat, H. (2017). Logging degrades nursery habitat for an iconic coral reef fish. *Biological Conservation* **210**, 273–280.

Hammer, L. J., Furey, N. B., Koh, W. Y. & Sulikowski, J. A. (2020). Movements of juvenile winter flounder in a southern Maine estuary. *Northeastern Naturalist* **27**(3), 502–519.

Hammerschlag, N. & Serafy, J. E. (2010). Nocturnal fish utilization of a subtropical mangrove-seagrass ecotone. *Marine Ecology-an Evolutionary Perspective* **31**(2), 364–374.

Harasti, D., Lee, K., Bruce, B., Gallen, C. & Bradford, R. (2017). Juvenile white sharks *Carcharodon carcharias* use estuarine environments in south-eastern Australia. *Marine Biology* **164**(3), 58.

Harding, J. M. & Mann, R. (2001). Diet and habitat use by bluefish, *Pomatomus saltatrix*, in a Chesapeake Bay estuary. *Environmental Biology of Fishes* **60**(4), 401–409.

Harding, J. M. & Mann, R. (2003). Influence of habitat on diet and distribution of striped bass (*Morone saxatilis*) in a temperate estuary. *Bulletin of Marine Science* **72**(3), 841–851.

Harlay, X., Koubbi, P. & Grioche, A. (2001). Ecology of plaice (*Pleuronectes platessa*) in fish assemblages of beaches of the Opale Coast (North of France) during spring 1997. *Cybium* **25**(1), 67–80.

Haynes, P. S., Brophy, D. & McGrath, D. (2011). The early life history of turbot (*Psetta maxima* L.) on nursery grounds along the west coast of Ireland: 2007–2009, as described by otolith microstructure. *Fisheries Research* **110**(3), 478–482.

Haynes, P. S., Brophy, D. & McGrath, D. (2012). Variability in the early life stages of juvenile plaice (*Pleuronectes platessa*) on west of Ireland nursery grounds: 2000–2007. *Journal of the Marine Biological Association of the United Kingdom* **92**(2), 395–406.

Haynes, P. S., Brophy, D., McGrath, D., O'Callaghan, R., Comerford, S. & Casburn, P. (2010). Annual and spatial variation in the abundance length and condition of juvenile turbot (*Psetta maxima* L.) on nursery grounds on the west coast of Ireland: 2000–2007. *Journal of Sea Research* **64**(4), 494–504.

Hazell, R. W. A., Schoeman, D. S. & Noffke, M. N. (2002). Do fluctuations in the somatic growth rate of rock lobster (*Jasus lalandii*) encompass all size classes? A re-assessment of juvenile growth. *Fishery Bulletin* **100**(3), 510–518.

Heck, K. L., Coen, L. D. & Morgan, S. G. (2001). Pre- and post-settlement factors as determinants of juvenile blue crab *Callinectes sapidus* abundance: results from the north-central Gulf of Mexico. *Marine Ecology Progress Series* **222**, 163–176.

Hegde, M. R., Padate, V. P. & Rivonker, C. U. (2016). Seasonal variations in habitat selection and catch trends of Sciaenids (Family: Sciaenidae) from the tropical waters off Goa, west coast of India. *Indian Journal of Geo-Marine Sciences* **45**(8), 943–951.

Henderson, A. C., Jourdan, A. & Bell, K. (2016). Assessing the incidental value of a marine reserve to a lemon shark *Negaprion brevirostris* nursery. *Aquatic Conservation– Marine and Freshwater Ecosystems* **26**(3), 482–491.

Henderson, A. C., McClellan, K. & Calosso, M. (2010). Preliminary assessment of a possible lemon shark nursery in the Turks & Caicos Islands, British West Indies. *Caribbean Journal of Science* **46**(1), 29–38.

Henderson, P. A. (2019). A long-term study of whiting, *Merlangius merlangus* (L) recruitment and population regulation in the Severn Estuary, UK. *Journal of Sea Research* **155**, 101825.

Henderson, P. A. & Seaby, R. M. (2005). The role of climate in determining the temporal variation in abundance, recruitment and growth of sole *Solea solea* in the Bristol Channel. *Journal of the Marine Biological Association of the United Kingdom* **85**(1), 197–204.

Henderson, P. A. & Seaby, R. M. H. (1994). On the factors influencing juvenile flatfish abundance in the lower Severn Estuary, England. *Netherlands Journal of Sea Research* **32**(3–4), 321–330.

Hendon, J. R. & Rakocinski, C. F. (2016). Habitat-specific growth, survival and diet of late juvenile hatchery-reared spotted seatrout (*Cynoscion nebulosus*). *Journal of Experimental Marine Biology and Ecology* **484**, 1–10.

Henriques, M. & Almada, V. C. (1998). Juveniles of non-resident fish found in sheltered rocky subtidal areas. *Journal of Fish Biology* **52**(6), 1301–1304.

Henry, F., Filipuci, I., Billon, G., Courcot, L., Kerambrun, E. & Amara, R. (2012). Metal concentrations, growth and condition indices in European juvenile flounder (*Platichthys flesus*) relative to sediment contamination levels in four Eastern English Channel estuaries. *Journal of Environmental Monitoring* **14**(12), 3211–3219.

Henseler, C., Kotterba, P., Bonsdorff, E., Nordstrom, M. C. & Oesterwind, D. (2020). Habitat utilization and feeding ecology of small round goby in a shallow brackish lagoon. *Marine Biodiversity* **50**(5), 88.

Hernandez, A., Plaza, G., Gutierrez, J., Cerna, F. & Niklitschek, E. J. (2020). Spatiotemporal analysis of the daily growth traits of the prerecruits of a small pelagic fish in response to environmental drivers. *Fisheries Oceanography* **29**(6), 457–469.

Herzka, S. Z., Holt, S. A. & Holt, G. J. (2002). Characterization of settlement patterns of red drum *Sciaenops ocellatus* larvae to estuarine nursery habitat: a stable isotope approach. *Marine Ecology Progress Series* **226**, 143–156.

Heupel, M. R. & Hueter, R. E. (2002). Importance of prey density in relation to the movement patterns of juvenile blacktip sharks (*Carcharhinus limbatus*) within a coastal nursery area. *Marine and Freshwater Research* **53**(2), 543–550.

Heupel, M. R. & Simpfendorfer, C. A. (2011). Estuarine nursery areas provide a low-mortality environment for young bull sharks *Carcharhinus leucas*. *Marine Ecology Progress Series* **433**, 237–244.

Heupel, M. R., Simpfendorfer, C. A. & Hueter, R. E. (2004). Estimation of shark home ranges using passive monitoring techniques. *Environmental Biology of Fishes* **71**(2), 135–142.

Hiddink, J. G., Marijnissen, S. A. E., Troost, K. & Wolff, W. J. (2002). Predation on O-group and older year classes of the bivalve *Macoma balthica*: interaction of size selection and intertidal distribution of epibenthic predators. *Journal of Experimental Marine Biology and Ecology* **269**(2), 223–248.

Hindell, J. S., Jenkins, G. P. & Keough, M. J. (2002). Variability in the numbers of post-settlement King George whiting (Sillaginidae: *Sillaginodes punctata*, Cuvier) in relation to predation, habitat complexity and artificial cage structure. *Journal of Experimental Marine Biology and Ecology* **268**(1), 13–31.

Hinz, H., Renones, O., Gouraguine, A., Johnson, A. F. & Moranta, J. (2019). Fish nursery value of algae habitats in temperate coastal reefs. *Peerj* **7**, e6797.

Hiraoka, Y., Fujioka, K., Fukuda, H., Watai, M. & Ohshimo, S. (2019). Interannual variation of the diet shifts and their effects on the fatness and growth of age-0 Pacific bluefin tuna (*Thunnus orientalis*) off the southwestern Pacific coast of Japan. *Fisheries Oceanography* **28**(4), 419–433.

Hixon, M. A. & Jones, G. P. (2005). Competition, predation, and density-dependent mortality in demersal marine fishes. *Ecology* **86**(11), 2847–2859.

Hobbs, J. A., Bennett, W. A. & Burton, J. E. (2006). Assessing nursery habitat quality for native smelts (Osmeridae) in the low-salinity zone of the San Francisco estuary. *Journal of Fish Biology* **69**(3), 907–922.

Holladay, B. A. & Norcross, B. L. (1995). August diet of age-0 Pacific halibut in nearshore waters of Kodiak Island, Alaska. *Environmental Biology of Fishes* **44**(4), 403–416.

Hollensead, L. D., Grubbs, R. D., Carlson, J. K. & Bethea, D. M. (2016). Analysis of fine-scale daily movement patterns of juvenile *Pristis pectinata* within a nursery habitat. *Aquatic Conservation-Marine and Freshwater Ecosystems* **26**(3), 492–505.

Hollensead, L. D., Grubbs, R. D., Carlson, J. K. & Bethea, D. M. (2018). Assessing residency time and habitat use of juvenile smalltooth sawfish using acoustic monitoring in a nursery habitat. *Endangered Species Research* **37**, 119–131.

Holsman, K. K., McDonald, P. S. & Armstrong, D. A. (2006). Intertidal migration and habitat use by subadult Dungeness crab *Cancer magister* in a NE Pacific estuary. *Marine Ecology Progress Series* **308**, 183–195.

Holst, J. C. & Slotte, A. (1998). Effects of juvenile nursery on geographic spawning distribution in Norwegian spring-spawning herring (*Clupea harengus* L.). *ICES Journal of Marine Science* **55**(6), 987–996.

Horinouchi, M. & Sano, M. (2001). Effects of changes in seagrass shoot density and leaf height on the abundance of juveniles of *Acentrogobius pflaumii* in a Zostera marina bed. *Ichthyological Research* **48**(2), 179–185.

Horinouchi, M. & Sano, R. (1999). Effects of changes in seagrass shoot density and leaf height on abundances and distribution patterns of juveniles of three gobiid fishes in a Zostera marina bed. *Marine Ecology Progress Series* **183**, 87–94.

Hovel, K. A. (2003). Habitat fragmentation in marine landscapes: relative effects of habitat cover and configuration on juvenile crab survival in California and North Carolina seagrass beds. *Biological Conservation* **110**(3), 401–412.

Hovel, K. A. & Fonseca, M. S. (2005). Influence of seagrass landscape structure on the juvenile blue crab habitat-survival function. *Marine Ecology Progress Series* **300**, 179–191.

Hovel, K. A. & Lipcius, R. N. (2001). Habitat fragmentation in a seagrass landscape: Patch size and complexity control blue crab survival. *Ecology* **82**(7), 1814–1829.

Hovel, K. A. & Lipcius, R. N. (2002). Effects of seagrass habitat fragmentation on juvenile blue crab survival and abundance. *Journal of Experimental Marine Biology and Ecology* **271**(1), 75–98.

Howarth, L. M., Pickup, S. E., Evans, L. E., Cross, T. J., Hawkins, J. P., Roberts, C. M. & Stewart, B. D. (2015*a*). Sessile and mobile components of a benthic ecosystem display mixed trends within a temperate marine reserve. *Marine Environmental Research* **107**, 8–23.

Howarth, L. M., Roberts, C. M., Hawkins, J. P., Steadman, D. J. & Beukers-Stewart, B. D. (2015*b*). Effects of ecosystem protection on scallop populations within a community-led temperate marine reserve. *Marine Biology* **162**(4), 823–840.

Howell, P. T., Molnar, D. R. & Harris, R. B. (1999). Juvenile winter flounder distribution by habitat type. *Estuaries* **22**(4), 1090–1095.

Hughes, B. B., Levey, M. D., Fountain, M. C., Carlisle, A. B., Chavez, F. P. & Gleason, M. G. (2015). Climate mediates hypoxic stress on fish diversity and nursery function at the land-sea interface. *Proceedings of the National Academy of Sciences of the United States of America* **112**(26), 8025–8030.

Huijbers, C. M., Grol, M. G. G. & Nagelkerken, I. (2008). Shallow patch reefs as alternative habitats for early juveniles of some mangrove/seagrass-associated fish species in Bermuda. *Revista De Biologia Tropical* **56**, 161–169.

Huijbers, C. M., Nagelkerken, I., Debrot, A. O. & Jongejans, E. (2013). Geographic coupling of juvenile and adult habitat shapes spatial population dynamics of a coral reef fish. *Ecology* **94**(8), 1859–1870.

Hurst, T. P. (2016). Shallow-water habitat use by Bering Sea flatfishes along the central Alaska Peninsula. *Journal of Sea Research* **111**, 37–46.

Hurst, T. P. & Abookire, A. A. (2006). Temporal and spatial variation in potential and realized growth rates of age-0 year northern rock sole. *Journal of Fish Biology* **68**(3), 905–919.

Hurst, T. P., Abookire, A. A. & Knoth, B. (2010). Quantifying thermal effects on contemporary growth variability to predict responses to climate change in northern rock sole (*Lepidopsetta polyxystra*). *Canadian Journal of Fisheries and Aquatic Sciences* **67**(1), 97–107.

Hurst, T. P., Cooper, D. W., Duffy-Anderson, J. T. & Farley, E. V. (2015). Contrasting coastal and shelf nursery habitats of Pacific cod in the southeastern Bering Sea. *ICES Journal of Marine Science* **72**(2), 515–527.

Hurst, T. P., Miller, J. A., Ferm, N., Heintz, R. A. & Farley, E. V. (2018). Spatial variation in potential and realized growth of juvenile Pacific cod in the southeastern Bering Sea. *Marine Ecology Progress Series* **590**, 171–185.

Hussey, N. E., DiBattista, J. D., Moore, J. W., Ward, E. J., Fisk, A. T., Kessel, S., Guttridge, T. L., Feldheim, K. A., Franks, B. R., Gruber, S. H., Weideli, O. C. & Chapman, D. D. (2017). Risky business for a juvenile marine predator? Testing the influence of foraging strategies on size and growth rate under natural conditions. *Proceedings of the Royal Society B – Biological Sciences* **284**(1852), 20170166.

Hussy, K., Mosegaard, H., Hinrichsen, H. H. & Bottcher, U. (2003). Using otolith microstructure to analyse growth of juvenile Baltic cod *Gadus morhua*. *Marine Ecology Progress Series* **258**, 233–241.

Huston, C. A., Stevens, P. W., Blaxton, R. M., Tolley, G., Scharer, R. M., Tornwall, B. M. & Poulakis, G. R. (2017). Diel movements of juvenile smalltooth sawfish: implications for defining the size of a nursery hotspot. *Endangered Species Research* **34**, 311–322.

Hwang, S. D., Lee, T. W. & Hwang, S. W. (2008). Age, growth and life history of gunnel, *Pholis fangi*, in the Yellow Sea. *Fisheries Research* **93**(1–2), 72–76.

Hyndes, G. A., Potter, I. C. & Hesp, S. A. (1996). Relationships between the movements, growth, age structures, anal reproductive biology of the teleosts *Sillago burrus* and *S. vittata* in temperate marine waters. *Marine Biology* **126**(3), 549–558.

Islam, M. S., Hibino, M., Nakayama, K. & Tanaka, M. (2006*a*). Condition of larval and early juvenile Japanese temperate bass *Lateolabrax japonicus* related to spatial distribution and feeding in the Chikugo estuarine nursery ground in the Ariake Bay, Japan. *Journal of Sea Research* **55**(2), 141–155.

Islam, M. S., Hibino, M. & Tanaka, M. (2006*b*). Distribution and dietary relationships of the Japanese temperate bass *Lateolabrax japonicus* juveniles with two contrasting copepod assemblages in estuarine nursery grounds in the Ariake Sea, Japan. *Journal of Fish Biology* **68**(2), 569–593.

Islam, M. S., Hibino, M. & Tanaka, M. (2007). Distribution and diet of the roughskin sculpin, *Trachidermus fasciatus*, larvae and juveniles in the Chikugo River estuary, Ariake Bay, Japan. *Ichthyological Research* **54**(2), 160–167.

Islam, M. S. & Tanaka, M. (2005). Nutritional condition, starvation status and growth of early juvenile Japanese sea bass (*Lateolabrax japonicus*) related to prey distribution and feeding in the nursery ground. *Journal of Experimental Marine Biology and Ecology* **323**(2), 172–183.

Islam, M. S. & Tanaka, M. (2006). Spatial variability in nursery functions along a temperate estuarine gradient: role of detrital versus algal trophic pathways. *Canadian Journal of Fisheries and Aquatic Sciences* **63**(8), 1848–1864.

Isnard, E., Tournois, J., McKenzie, D. J., Ferraton, F., Bodin, N., Aliaume, C. & Darnaude, A. M. (2015). Getting a good start in life? A comparative analysis of the quality of lagoons as juvenile habitats for the gilthead seabream *Sparus aurata* in the Gulf of Lions. *Estuaries and Coasts* **38**(6), 1937–1950.

James, N. C., Leslie, T. D., Potts, W. M., Whitfield, A. K. & Rajkaran, A. (2019). The importance of different juvenile habitats as nursery areas for a ubiquitous estuarine-dependent marine fish species. *Estuarine Coastal and Shelf Science* **226**, 106270.

Jaxion-Harm, J. & Szedlmayer, S. T. (2015). Depth and artificial reef type effects on size and distribution of red snapper in the northern Gulf of Mexico. *North American Journal of Fisheries Management* **35**(1), 86–96.

Jayawardane, P., McClusky, D. S. & Tytler, P. (2002). Factors influencing migration of *Penaeus indicus* in the Negombo lagoon on the west coast of Sri Lanka. *Fisheries Management and Ecology* **9**(6), 351–363.

Jelbart, J. E., Ross, P. M. & Connolly, R. M. (2007*a*). Fish assemblages in seagrass beds are influenced by the proximity of mangrove forests. *Marine Biology* **150**(5), 993–1002.

Jelbart, J. E., Ross, P. M. & Connolly, R. M. (2007*b*). Patterns of small fish distributions in seagrass beds in a temperate Australian estuary. *Journal of the Marine Biological Association of the United Kingdom* **87**(5), 1297–1307.

Jennings, D. E., Gruber, S. H., Franks, B. R., Kessel, S. T. & Robertson, A. L. (2008). Effects of large-scale anthropogenic development on juvenile lemon shark (*Negaprion brevirostris*) populations of Bimini, Bahamas. *Environmental Biology of Fishes* **83**(4), 369–377.

Jennings, S., Lancaster, J. E., Ryland, J. S. & Shackley, S. E. (1991). The age structure and growth dynamics of young-of-the-year bass, *Dicentrarchus labrax*, populations. *Journal of the Marine Biological Association of the United Kingdom* **71**(4), 799–810.

Jin, B. S., Xu, W., Guo, L., Chen, J. K. & Fu, C. Z. (2014). The impact of geomorphology of marsh creeks on fish assemblage in Changjiang River estuary. *Chinese Journal of Oceanology and Limnology* **32**(2), 469–479.

Johnson, D. S. & Williams, B. L. (2017). Sea level rise may increase extinction risk of a saltmarsh ontogenetic habitat specialist. *Ecology and Evolution* **7**(19), 7786–7795.

Johnson, D. W. (2007). Habitat complexity modifies post-settlement mortality and recruitment dynamics of a marine fish. *Ecology* **88**(7), 1716–1725.

Johnson, E. G. & Eggleston, D. B. (2010). Population density, survival and movement of blue crabs in estuarine salt marsh nurseries. *Marine Ecology Progress Series* **407**, 135–147.

Johnson, K. D., Grabowski, J. H. & Smee, D. L. (2014). Omnivory dampens trophic cascades in estuarine communities. *Marine Ecology Progress Series* **507**, 197–206.

Johnston, C. A. & Lipcius, R. N. (2012). Exotic macroalga *Gracilaria vermiculophylla* provides superior nursery habitat for native blue crab in Chesapeake Bay. *Marine Ecology Progress Series* **467**, 137–146.

Jokinen, H., Wennhage, H., Ollus, V., Aro, E. & Norkko, A. (2016). Juvenile flatfish in the northern Baltic Sea – long-term decline and potential links to habitat characteristics. *Journal of Sea Research* **107**, 67–75.

Jones, C. L., Anderson, T. W. & Edwards, M. S. (2013). Evaluating eelgrass site quality by the settlement, performance, and survival of a marine fish. *Journal of Experimental Marine Biology and Ecology* **445**, 61–68.

Jones, D. L., Walter, J. F., Brooks, E. N. & Serafy, J. E. (2010). Connectivity through ontogeny: fish population linkages among mangrove and coral reef habitats. *Marine Ecology Progress Series* **401**, 245–258.

Jordan, L. K. B., Lindeman, K. C. & Spieler, R. E. (2012). Depth-variable settlement patterns and predation influence on newly settled reef fishes (*Haemulon* spp., Haemulidae). *PLoS One* **7**(12), e50897.

Joseph, V., Locke, A. & Godin, J. G. J. (2006). Spatial distribution of fishes and decapods in eelgrass (*Zostera marina* L.) and sandy habitats of a New Brunswick estuary, eastern Canada. *Aquatic Ecology* **40**(1), 111–123.

Joyeux, E., Carpentier, A., Corre, F., Haie, S. & Petillon, J. (2017). Impact of salt-marsh management on fish nursery function in the bay of Aiguillon (French Atlantic coast), with a focus on European sea bass diet. *Journal of Coastal Conservation* **21**(3), 435–444.

Juanes, F., Clarke, P. J. & Murt, J. (2013). Fall and winter estuarine recruitment of bluefish *Pomatomus saltatrix*: selectivity for large lipid-rich prey increases depleted energy levels. *Marine Ecology Progress Series* **492**, 235–252.

Jud, Z. R., Layman, C. A. & Shenker, J. M. (2011). Diet of age-0 tarpon (*Megalops atlanticus*) in anthropogenically-modified and natural nursery habitats along the Indian River Lagoon, Florida. *Environmental Biology of Fishes* **90**(3), 223–233.

Kafayat, A. F., Martins, A. A., Shehu, L. A., Abdulwakil, O. S. & Abass, M. A. (2015). Life-stages, exploitation status and habitat use of *Lutjanus goreensis* (Perciformes: Lutjanidae) in coastal marine environments of Lagos, SW Nigeria. *Revista De Biologia Tropical* **63**(1), 199–212.

Kaifu, K., Miller, M. J., Aoyama, J., Washitani, I. & Tsukamoto, K. (2013). Evidence of niche segregation between freshwater eels and conger eels in Kojima Bay, Japan. *Fisheries Science* **79**(4), 593–603.

Kamenos, N. A., Moore, P. G. & Hall-Spencer, J. M. (2004*a*). Nursery-area function of maerl grounds for juvenile queen scallops *Aequipecten opercularis* and other invertebrates. *Marine Ecology Progress Series* **274**, 183–189.

Kamenos, N. A., Moore, P. G. & Hall-Spencer, J. M. (2004*b*). Small-scale distribution of juvenile gadoids in shallow inshore waters; what role does maerl play? *ICES Journal of Marine Science* **61**(3), 422–429.

Kamermans, P., Guindon, K. Y. & Miller, J. M. (1995). Importance of food availability for growth of juvenile southern flounder (*Paralichthys lethostigma*) in the Pamlico River estuary, North Carolina, USA. *Netherlands Journal of Sea Research* **34**(1–3), 101–109.

Kamimura, Y. & Shoji, J. (2013). Does macroalgal vegetation cover influence post-settlement survival and recruitment potential of juvenile black rockfish *Sebastes cheni*? *Estuarine Coastal and Shelf Science* **129**, 86–93.

Kanno, S., Schlaff, A. M., Heupel, M. R. & Simpfendorfer, C. A. (2019). Stationary video monitoring reveals habitat use of stingrays in mangroves. *Marine Ecology Progress Series* **621**, 155–168.

Kanou, K., Sano, M. & Kohno, H. (2007). Relationships between short-term variations in density of juvenile yellowfin goby *Acanthogobius flavimanus* and environmental variables on an estuarine mudflat. *Fisheries Science* **73**(1), 38–45.

Kanstinger, P. & Peck, M. A. (2009). Co-occurrence of European sardine (*Sardina pilchardus*), anchovy (*Engraulis encrasicolus*) and sprat (*Sprattus sprattus*) larvae in southern North Sea habitats: Abundance, distribution and biochemical–based condition. *Scientia Marina* **73**, 141–152.

Kato, Y., Togashi, H., Kurita, Y., Kamauchi, H. & Tayasu, I. (2020). Discrimination of nursery locations of juvenile Japanese flounder *Paralichthys olivaceus* on the Pacific coast of northern Japan based on carbon and nitrogen stable isotope ratios. *Fisheries Science* **86**(4), 615–623.

Keller, D. A., Gittman, R. K., Bouchillon, R. K. & Fodrie, F. J. (2017). Life stage and species identity affect whether habitat subsidies enhance or simply redistribute consumer biomass. *Journal of Animal Ecology* **86**(6), 1394–1403.

Kelley, D. (2002). Abundance, growth and first-winter survival of young bass in nurseries of south-west England. *Journal of the Marine Biological Association of the United Kingdom* **82**(2), 307–319.

Kellison, G. T., Eggleston, D. B., Taylor, J. C. & Burke, J. S. (2003). An assessment of biases associated with caging, tethering, and trawl sampling of summer flounder (*Paralichthys dentatus*). *Estuaries* **26**(1), 64–71.

Kenyon, R. A., Loneragan, N. R., Hughes, J. M. & Staples, D. J. (1997). Habitat type influences the microhabitat preference of juvenile tiger prawns (*Penaeus esculentus* Haswell and *Penaeus semisulcatus* de Haan). *Estuarine Coastal and Shelf Science* **45**(3), 393–403.

Kimirei, I. A., Nagelkerken, I., Mgaya, Y. D. & Huijbers, C. M. (2013). The mangrove nursery paradigm revisited: otolith stable isotopes support nursery-to-reef movements by Indo-Pacific fishes. *PLoS One* **8**(6), e66320.

Kimirei, I. A., Nagelkerken, I., Slooter, N., Gonzalez, E. T., Huijbers, C. M., Mgaya, Y. D. & Rypel, A. L. (2015). Demography of fish populations reveals new challenges in appraising juvenile habitat values. *Marine Ecology Progress Series* **518**, 225–237.

Kinney, M. J., Kacev, D., Sippel, T., Dewar, H. & Eguchi, T. (2020). Common thresher shark *Alopias vulpinus* movement: Bayesian inference on a data-limited species. *Marine Ecology Progress Series* **639**, 155–167.

Kisten, Y., Edworthy, C. & Strydom, N. A. (2020). Fine-scale habitat use by larval fishes in the Swartkops Estuary, South Africa. *Environmental Biology of Fishes* **103**(1), 125–136.

Kisten, Y., Pattrick, P., Strydom, N. A. & Perissinotto, R. (2015). Dynamics of recruitment of larval and juvenile Cape stumpnose *Rhabdosargus holubi* (Teleostei: Sparidae) into the Swartkops and Sundays estuaries, South Africa. *African Journal of Marine Science* **37**(1), 1–10.

Klein, M., Van Beveren, E., Rodrigues, D., Serrao, E. A., Caselle, J. E., Goncalves, E. J. & Borges, R. (2018). Small scale temporal patterns of recruitment and hatching of Atlantic horse mackerel (L.) at a nearshore reef area. *Fisheries Oceanography* **27**(6), 505–516.

Kneebone, J., Chisholm, J. & Skomal, G. (2014). Movement patterns of juvenile sand tigers (*Carcharias taurus*) along the east coast of the USA. *Marine Biology* **161**(5), 1149–1163.

Kneib, R. T. (2009). Genotypic variation does not explain differences in growth of mummichogs *Fundulus heteroclitus* from simple and complex tidal marsh landscapes. *Marine Ecology Progress Series* **386**, 207–219.

Knip, D. M., Heupel, M. R., Simpfendorfer, C. A., Tobin, A. J. & Moloney, J. (2011). Ontogenetic shifts in movement and habitat use of juvenile pigeye sharks *Carcharhinus amboinensis* in a tropical nearshore region. *Marine Ecology Progress Series* **425**, 233–246.

Koenig, C. C., Coleman, F. C., Eklund, A. M., Schull, J. & Ueland, J. (2007). Mangroves as essential nursery habitat for goliath grouper (*Epinephelus itajara*). *Bulletin of Marine Science* **80**(3), 567–585.

Kostecki, C., Rochette, S., Girardin, R., Blanchard, M., Desroy, N. & Le Pape, O. (2011). Reduction of flatfish habitat as a consequence of the proliferation of an invasive mollusc. *Estuarine Coastal and Shelf Science* **92**(1), 154–160.

Kostecki, C., Roussel, J. M., Desroy, N., Roussel, G., Lanshere, J., Le Bris, H. & Le Pape, O. (2012). Trophic ecology of juvenile flatfish in a coastal nursery ground: contributions of intertidal primary production and freshwater particulate organic matter. *Marine Ecology Progress Series* **449**, 221–232.

Kramer, S. H. (1991). Growth, mortality, and movements of juvenile California halibut *Paralichthys californicus* in shallow coastal and bay habitats of San Diego county, California. *Fishery Bulletin* **89**(2), 195–207.

Kraus, R. T. & Secor, D. H. (2005). Application of the nursery-role hypothesis to an estuarine fish. *Marine Ecology Progress Series* **291**, 301–305.

Kruck, N. C., Chargulaf, C. A., Saint-Paul, U. & Tibbetts, I. R. (2009). Early post-settlement habitat and diet shifts and the nursery function of tidepools during *Sillago* spp. recruitment in Moreton Bay, Australia. *Marine Ecology Progress Series* **384**, 207–219.

Kulp, R. E. & Peterson, B. J. (2016). Evaluating the impact of mesopredators on oyster restoration in the new york metropolitan region. *Journal of Shellfish Research* **35**(4), 801–807.

Kume, G., Yagishita, N., Furumitsu, K., Nakata, H., Suzuki, T., Handa, M. & Yamaguchi, A. (2015). The role of molecular methods to compare distribution and feeding habits in larvae and juveniles of two co-occurring sciaenid species *Nibea albiflora* and *Pennahia argentata*. *Estuarine Coastal and Shelf Science* **167**, 516–525.

Kurita, Y., Okazaki, Y. & Yamashita, Y. (2018). Ontogenetic habitat shift of age-0 Japanese flounder *Paralichthys olivaceus* on the Pacific coast of northeastern Japan: differences in timing of the shift among areas and potential effects on recruitment success. *Fisheries Science* **84**(2), 173–187.

Kurita, Y., Uehara, S., Okazaki, Y., Sakami, T., Nambu, R. & Tomiyama, T. (2017). Impact of the great tsunami in 2011 on the quality of nursery grounds for juvenile Japanese flounder *Paralichthys olivaceus* in Sendai Bay, Japan. *Fisheries Oceanography* **26**(2), 165–180.

Kurth, B. N., Peebles, E. B. & Stallings, C. D. (2019). Atlantic Tarpon (*Megalops atlanticus*) exhibit upper estuarine habitat dependence followed by foraging system fidelity after ontogenetic habitat shifts. *Estuarine Coastal and Shelf Science* **225**, 106248.

La Mesa, G., Louisy, P. & Vacchi, M. (2002). Assessment of microhabitat preferences in juvenile dusky grouper (*Epinephelus marginatus*) by visual sampling. *Marine Biology* **140**(1), 175–185.

Laffaille, P., Lefeuvre, J. C., Schricke, M. T. & Feunteun, E. (2001). Feeding ecology of 0-group sea bass, *Dicentrarchus labrax*, in salt marshes of Mont Saint Michel Bay (France). *Estuaries* **24**(1), 116–125.

Laffaille, P., Pétillon, J., Parlier, E., Valéry, L., Ysnel, F., Radureau, A., Feunteun, E. & Lefeuvre, J. C. (2005). Does the invasive plant *Elymus athericus* modify fish diet in tidal salt marshes? *Estuarine Coastal and Shelf Science* **65**(4), 739–746.

Laffargue, P., Lagardere, F., Rijnsdorp, A. D., Fillon, A. & Amara, R. (2007). Growth performances of juvenile sole *Solea solea* under environmental constraints of embayed nursery areas. *Aquatic Living Resources* **20**(3), 213–221.

Laidig, T. E. (2010). Influence of ocean conditions on the timing of early life history events for blue rockfish (*Sebastes mystinus*) off California. *Fishery Bulletin* **108**(4), 442–449.

Lancaster, J. E., Pawsons, M. G., Pickett, G. D. & Jennings, S. (1998). The impact of the 'Sea Empress' oil spill on seabass recruitment. *Marine Pollution Bulletin* **36**(9), 677–688.

Langan, J. A., McManus, M. C., Zemeckis, D. R. & Collie, J. S. (2020). Abundance and distribution of Atlantic cod (*Gadus morhua*) in a warming southern New England. *Fishery Bulletin* **118**(2), 145–156.

Laurel, B. J., Knoth, B. A. & Ryer, C. H. (2016). Growth, mortality, and recruitment signals in age-0 gadids settling in coastal Gulf of Alaska. *ICES Journal of Marine Science* **73**(9), 2227–2237.

Laurel, B. J., Ryer, C. H., Knoth, B. & Stoner, A. W. (2009). Temporal and ontogenetic shifts in habitat use of juvenile Pacific cod (*Gadus macrocephalus*). *Journal of Experimental Marine Biology and Ecology* **377**(1), 28–35.

Laurel, B. J., Ryer, C. H., Spencer, M., Iseri, P., Knoth, B. & Stoner, A. (2012). Effects of natural and anthropogenic disturbance on polychaete worm tubes and age-0 flatfish distribution. *Marine Ecology Progress Series* **466**, 193–203.

Lawrie, S. M. & McQuaid, C. D. (2001). Scales of mussel bed complexity: structure, associated biota and recruitment. *Journal of Experimental Marine Biology and Ecology* **257**(2), 135–161.

Lazzari, M. A. (2008). Habitat variability in young-of-the-year winter flounder, *Pseudopleuronectes americanus*, in Maine estuaries. *Fisheries Research* **90**(1–3), 296–304.

Lazzari, M. A. (2013). Use of submerged aquatic vegetation by young-of-the-year gadoid fishes in Maine estuaries. *Journal of Applied Ichthyology* **29**(2), 404–409.

Lazzari, M. A. (2015). Eelgrass, *Zostera marina*, as essential fish habitat for young-of-the-year winter flounder, *Pseudopleuronectes americanus* (Walbaum, 1792) in Maine estuaries. *Journal of Applied Ichthyology* **31**(3), 459–465.

Lazzari, M. A. & Stone, B. Z. (2006). Use of submerged aquatic vegetation as habitat by young-of-the-year epibenthic fishes in shallow Maine nearshore waters. *Estuarine Coastal and Shelf Science* **69**(3–4), 591–606.

Le, D. Q., Fui, S. Y., Piah, R. M., Ishimura, T., Sano, Y., Tanaka, K. & Shirai, K. (2019). Isotopic evidence of connectivity between an inshore vegetated lagoon (nursery habitat) and coastal artificial reefs (adult habitats) for the reef fish *Lethrinus lentjan* on the Terengganu coast, Malaysia. *Marine and Freshwater Research* **70**(12), 1675–1688.

Le, D. Q., Fui, S. Y., Tanaka, K., Suratman, S., Sano, Y. & Shirai, K. (2020). Feeding habitats of juvenile reef fishes in a tropical mangrove–seagrass continuum along a Malaysian shallow-water coastal lagoon. *Bulletin of Marine Science* **96**(3), 469–486.

Le, D. Q., Tanaka, K., Hii, Y. S., Sano, Y., Nanjo, K. & Shirai, K. (2018). Importance of seagrass-mangrove continuum as feeding grounds for juvenile pink ear emperor *Lethrinus lentjan* in Setiu Lagoon, Malaysia: Stable isotope approach. *Journal of Sea Research* **135**, 1–10.

Le Luherne, E., Le Pape, O., Murillo, L., Randon, M., Lebot, C. & Reveillac, E. (2017). Influence of green tides in coastal nursery grounds on the habitat selection and individual performance of juvenile fish. *PLoS One* **12**(1), e170110.

Le Pape, O., Baulier, L., Cloarec, A., Martin, J., Le Loc'h, F. & Desaunay, Y. (2007). Habitat suitability for juvenile common sole (*Solea solea*, L.) in the Bay of Biscay (France): A quantitative description using indicators based on epibenthic fauna. *Journal of Sea Research* **57**(2–3), 126–136.

Le Pape, O., Chauvet, F., Desaunay, Y. & Guerault, D. (2003*a*). Relationship between interannual variations of the river plume and the extent of nursery grounds for the common sole (*Solea solea*, L.) in Vilaine Bay. Effects on recruitment variability. *Journal of Sea Research* **50**(2–3), 177–185.

Le Pape, O., Chauvet, F., Mahevas, S., Lazure, P., Guerault, D. & Desaunay, Y. (2003*b*). Quantitative description of habitat suitability for the juvenile common sole (*Solea solea*, L.) in the Bay of Biscay (France) and the contribution of different habitats to the adult population. *Journal of Sea Research* **50**(2–3), 139–149.

Le Pape, O., Guerault, D. & Desaunay, Y. (2004). Effect of an invasive mollusc, American slipper limpet *Crepidula fornicata*, on habitat suitability for juvenile common sole *Solea solea* in the Bay of Biscay. *Marine Ecology Progress Series* **277**, 107–115.

Le Pape, O., Holley, J., Guerault, D. & Desaunay, Y. (2003*c*). Quality of coastal and estuarine essential fish habitats: estimations based on the size of juvenile common sole (*Solea solea* L.). *Estuarine Coastal and Shelf Science* **58**(4), 793–803.

Le Pape, O., Moderan, J., Beaunee, G., Riera, P., Nicolas, D., Savoye, N., Harmelin-Vivien, M., Darnaude, A. M., Brind'Amour, A., Le Bris, H., Cabral, H., Vinagre, C., Pasquaud, S., Franca, S. & Kostecki, C. (2013). Sources of organic matter for flatfish juveniles in coastal and estuarine nursery grounds: A meta-analysis for the common sole (*Solea solea*) in contrasted systems of Western Europe. *Journal of Sea Research* **75**, 85–95.

Lear, K. O., Poulakis, G. R., Scharer, R. M., Gleiss, A. C. & Whitney, N. M. (2019). Fine-scale behavior and habitat use of the endangered smalltooth sawfish (*Pristis pectinata*): insights from accelerometry. *Fishery Bulletin* **117**(4), 348–359.

Lebreton, B., Richard, P., Guillou, G. & Blanchard, G. F. (2013). Trophic shift in young-of-the-year Mugilidae during salt-marsh colonization. *Journal of Fish Biology* **82**(4), 1297–1307.

Leclus, F., Hennig, H., Melo, Y. C. & Boyd, A. J. (1994). Impact of the extent and locality of mud patches on the density and geographic distribution of juvenile Agulhas sole *Austroglossus pectoralis* (Soleidae). *South African Journal of Marine Science* **14**, 19–36.

Lee, C. L. & Lin, H. J. (2015). Ontogenetic habitat utilization patterns of juvenile reef fish in low-predation habitats. *Marine Biology* **162**(9), 1799–1811.

Lee, C. L., Wen, C. K. C., Huang, Y. H., Chung, C. Y. & Lin, H. J. (2019). Ontogenetic habitat usage of juvenile carnivorous fish among seagrass-coral mosaic habitats. *Diversity* **11**(2), 25.

Legare, B., Kneebone, J., DeAngelis, B. & Skomal, G. (2015). The spatiotemporal dynamics of habitat use by blacktip (*Carcharhinus limbatus*) and lemon (*Negaprion brevirostris*) sharks in nurseries of St. John, United States Virgin Islands. *Marine Biology* **162**(3), 699–716.

Leo, J. P., Minello, T. J. & Grant, W. E. (2018). Assessing variability in juvenile brown shrimp growth rates in small marsh ponds: an exercise in model evaluation and improvement. *Marine and Coastal Fisheries* **10**(3), 347–356.

Leone, C., Zucchetta, M., Capoccioni, F., Gravina, M. F., Franzoi, P. & Ciccotti, E. (2016). Stage-specific distribution models can predict eel (*Anguilla anguilla*) occurrence during settlement in coastal lagoons. *Estuarine Coastal and Shelf Science* **170**, 123–133.

Leslie, T., James, N. C., Potts, W. M. & Rajkaran, A. (2017). The relationship between habitat complexity and nursery provision for an estuarine-dependent fish species in a permanently open South African Estuary. *Estuarine Coastal and Shelf Science* **198**, 183–192.

Levin, P. S. (1991). Effects of microhabitat on recruitment variation in a gulf of maine reef fish. *Marine Ecology Progress Series* **75**(2–3), 183–189.

Lewis, N. S., Young, D. R., Folger, C. L. & DeWitt, T. H. (2021). Assessing the relative importance of estuarine nursery habitats – a Dungeness crab (*Cancer magister*) case study. *Estuaries and Coasts* **44**(4), 1062–1073.

Ley, J. A. & Rolls, H. J. (2018). Using otolith microchemistry to assess nursery habitat contribution and function at a fine spatial scale. *Marine Ecology Progress Series* **606**, 151–173.

Lin, N., Chen, Y. G., Jin, Y., Yuan, X. W., Ling, J. Z. & Jiang, Y. Z. (2018). Distribution of the early life stages of small yellow croaker in the Yangtze River estuary and adjacent waters. *Fisheries Science* **84**(2), 357–363.

Lindberg, W. J., Frazer, T. K., Portier, K. M., Vose, F., Loftin, J., Murie, D. J., Mason, D. M., Nagy, B. & Hart, M. K. (2006). Density-dependent habitat selection and performance by a large mobile reef fish. *Ecological Applications* **16**(2), 731–746.

Lipcius, R. N., Eggleston, D. B., Miller, D. L. & Luhrs, T. C. (1998). The habitat-survival function for Caribbean spiny lobster: an inverted size effect and non-linearity in mixed algal and seagrass habitats. *Marine and Freshwater Research* **49**(8), 807–816.

Lipcius, R. N., Seitz, R. D., Seebo, M. S. & Colon-Carrion, D. (2005). Density, abundance and survival of the blue crab in seagrass and unstructured salt marsh nurseries of Chesapeake Bay. *Journal of Experimental Marine Biology and Ecology* **319**(1–2), 69–80.

Litvin, S. Y., Weinstein, M. P. & Guida, V. G. (2014). Habitat utilization patterns determine the physiological condition of *Cynoscion regalis* during estuarine residency. *Marine Ecology Progress Series* **510**, 87–99.

Loher, T. & Armstrong, D. A. (2000). Effects of habitat complexity and relative larval supply on the establishment of early benthic phase red king crab (*Paralithodes camtschaticus* Tilesius, 1815) populations in Auke Bay, Alaska. *Journal of Experimental Marine Biology and Ecology* **245**(1), 83–109.

Lohrer, A. M., McCartain, L. D., Buckthought, D., MacDonald, L. & Parsons, D. M. (2018). Benthic structure and pelagic food sources determine post-settlement snapper (*Chrysophrys auratus*) abundance. *Frontiers in Marine Science* **5**, 427.

Loneragan, N. R., Kangas, M., Haywood, M. D. E., Kenyon, R. A., Caputi, N. & Sporer, E. (2013). Impact of cyclones and aquatic macrophytes on recruitment and landings of tiger prawns *Penaeus esculentus* in Exmouth Gulf, Western Australia. *Estuarine Coastal and Shelf Science* **127**, 46–58.

Loneragan, N. R., Kenyon, R. A., Haywood, M. D. E. & Staples, D. J. (1994). Population dynamics of juvenile tiger prawns (*Penaeus esculentus* and *P. semisulcatus*) in seagrass habitats of the western Gulf of Carpentaria, Australia. *Marine Biology* **119**(1), 133–143.

Loneragan, N. R., Kenyon, R. A., Staples, D. J., Poiner, I. R. & Conacher, C. A. (1998). The influence of seagrass type on the distribution and abundance of postlarval and juvenile tiger prawns (*Penaeus esculentus* and *P. semisulcatus*) in the western Gulf of Carpentaria, Australia. *Journal of Experimental Marine Biology and Ecology* **228**(2), 175–195.

Long, W. C., Grow, J. N., Majoris, J. E. & Hines, A. H. (2011). Effects of anthropogenic shoreline hardening and invasion by *Phragmites australis* on habitat quality for juvenile blue crabs (*Callinectes sapidus*). *Journal of Experimental Marine Biology and Ecology* **409**(1–2), 215–222.

Long, W. C., Sellers, A. J. & Hines, A. H. (2013). Mechanism by which coarse woody debris affects predation and community structure in Chesapeake Bay. *Journal of Experimental Marine Biology and Ecology* **446**, 297–305.

Lopez-Rasgado, F. J. & Herzka, S. Z. (2009). Assessment of habitat quality for juvenile California halibut (*Paralichthys californicus*) in a seasonally arid estuary. *Fishery Bulletin* **107**(3), 343–358.

Love, J. W., Johnson, A. K. & May, E. B. (2006*a*). Spatial and temporal differences of Atlantic Menhaden (*Brevoortia tyrannus*) recruitment across major Drainages (1966–2004) of the Chesapeake Bay watershed. *Estuaries and Coasts* **29**(5), 794–801.

Love, M. S., Schroeder, D. M., Lenarz, W., MacCall, A., Bull, A. S. & Thorsteinson, L. (2006*b*). Potential use of offshore marine structures in rebuilding an overfished rockfish species, bocaccio (*Sebastes paucispinis*). *Fishery Bulletin* **104**(3), 383–390.

Lowe, M. L., Morrison, M. A. & Taylor, R. B. (2015). Harmful effects of sediment-induced turbidity on juvenile fish in estuaries. *Marine Ecology Progress Series* **539**, 241–254.

Lugendo, B. R., Pronker, A., Cornelissen, I., de Groene, A., Nagelkerken, I., Dorenbosch, M., van der Velde, G. & Mgaya, Y. D. (2005). Habitat utilisation by juveniles of commercially important fish species in a marine embayment in Zanzibar, Tanzania. *Aquatic Living Resources* **18**(2), 149–158.

Lyons, K., Galloway, A. S., Adams, D. H., Reyier, E. A., Barker, A. M., Portnoy, D. S. & Frazier, B. S. (2020). Maternal provisioning gives young-of-the-year Hammerheads a head start in early life. *Marine Biology* **167**(11), 157.

Mace, M. M., Kimball, M. E. & Haffey, E. R. (2018). Recruitment and habitat use of early life stage tarpon (*Megalops atlanticus*) in South Carolina estuaries. *Estuaries and Coasts* **41**(3), 841–854.

Mace, M. M. & Rozas, L. P. (2017). Population dynamics and secondary production of juvenile white shrimp (*Litopenaeus setiferus*) along an estuarine salinity gradient. *Fishery Bulletin* **115**(1), 74–88.

Machut, L. S., Limburg, K. E., Schmidt, R. E. & Dit-Rman, D. (2007). Anthropogenic impacts on American eel demographics in Hudson River tributaries, New York. *Transactions of the American Fisheries Society* **136**(6), 1699–1713.

Maciel, T. R., Avigliano, E., de Carvalho, B. M., Miller, N. & Vianna, M. (2020). Population structure and habitat connectivity of *Genidens genidens* (Siluriformes) in tropical and subtropical coasts from Southwestern Atlantic. *Estuarine Coastal and Shelf Science* **242**, 106839.

MacPherson, E. (1998). Ontogenetic shifts in habitat use and aggregation in juvenile sparid fishes. *Journal of Experimental Marine Biology and Ecology* **220**(1), 127–150.

Macpherson, E., Biagi, F., Francour, P., Garcia-Rubies, A., Harmelin, J., Harmelin-Vivien, M., Jouvenel, J. Y., Planes, S., Vigliola, L. & Tunesi, L. (1997). Mortality of juvenile fishes of the genus *Diplodus* in protected and unprotected areas in the western Mediterranean Sea. *Marine Ecology Progress Series* **160**, 135–147.

Mahardja, B., Hobbs, J. A., Ikemiyagi, N., Benjamin, A. & Finger, A. J. (2019). Role of freshwater floodplain-tidal slough complex in the persistence of the endangered delta smelt. *PLoS One* **14**(1), e0208084.

Mai, A. C. G., Albuquerque, C. Q., Lemos, V. M., Schwingel, P. R., Ceni, G. F., Saint'Pierre, T. D. & Vieira, J. P. (2019). Coastal zone use and migratory behaviour of the southern population of *Mugil liza* in Brazil. *Journal of Fish Biology* **95**(5), 1207–1214.

Malloy, K. D., Yamashita, Y., Yamada, H. & Targett, T. E. (1996). Spatial and temporal patterns of juvenile stone flounder *Kareius bicoloratus* growth rates during and after settlement. *Marine Ecology Progress Series* **131**(1–3), 49–59.

Mamauag, S. S., Alino, P. M., Gonzales, R. O. M. & Deocadez, M. R. (2009). Patterns of demersal fish distribution derived from line fishing experiment in Calauag Bay, Philippines. *Philippine Agricultural Scientist* **92**(4), 370–387.

Manderson, J. P., Pessutti, J., Hilbert, J. G. & Juanes, F. (2004). Shallow water predation risk for a juvenile flatfish (winter flounder; *Pseudopleuronectes americanus*, Walbaum) in a northwest Atlantic estuary. *Journal of Experimental Marine Biology and Ecology* **304**(2), 137–157.

Manderson, J. P., Pessutti, J., Meise, C., Johnson, D. & Shaheen, P. (2003). Winter flounder settlement dynamics and the modification of settlement patterns by post-settlement processes in a NW Atlantic estuary. *Marine Ecology Progress Series* **253**, 253–267.

Manderson, J. P., Pessutti, J., Shaheen, P. & Juanes, F. (2006). Dynamics of early juvenile winter flounder predation risk on a North West Atlantic estuarine nursery ground. *Marine Ecology Progress Series* **328**, 249–265.

Manderson, J. P., Phelan, B. A., Meise, C., Stehlik, L. L., Bejda, A. J., Pessutti, J., Arlen, L., Draxler, A. & Stoner, A. W. (2002). Spatial dynamics of habitat suitability for the growth of newly settled winter flounder *Pseudopleuronectes americanus* in an estuarine nursery. *Marine Ecology Progress Series* **228**, 227–239.

Manderson, J. P., Phelan, B. A., Stoner, A. W. & Hilbert, J. (2000). Predator-prey relations between age-1 + summer flounder (*Paralichthys dentatus*, Linnaeus) and age-0 winter flounder (*Pseudopleuronectes americanus*, Walbaum): predator diets, prey selection, and effects of sediments and macrophytes. *Journal of Experimental Marine Biology and Ecology* **251**(1), 17–39.

Maravelias, C. & Papaconstantinou, C. (2003). Size-related habitat use, aggregation patterns and abundance of anglerfish (*Lophius budegassa*) in the Mediterranean Sea determined by generalized additive modelling. *Journal of the Marine Biological Association of the United Kingdom* **83**(5), 1171–1178.

Marie, A. D., Miller, C., Cawich, C., Piovano, S. & Rico, C. (2017). Fisheries-independent surveys identify critical habitats for young scalloped hammerhead sharks (*Sphyrna lewini*) in the Rewa Delta, Fiji. *Scientific Reports* **7**, 17273.

Markel, R. W., Lotterhos, K. E. & Robinson, C. L. K. (2017). Temporal variability in the environmental and geographic predictors of spatial-recruitment in nearshore rockfishes. *Marine Ecology Progress Series* **574**, 97–111.

Marks, R., Hesp, S. A., Johnston, D., Denhann, A. & Loneragan, N. (2020). Temporal changes in the growth of a crustacean species, *Portunus armatus*, in a temperate marine embayment: evidence of density dependence. *ICES Journal of Marine Science* **77**(2), 773–790.

Marsh, J. M., Mueter, F. J. & Quinn, T. J. (2020). Environmental and biological influences on the distribution and population dynamics of polar cod (*Boreogadus saida*) in the US Chukchi Sea. *Polar Biology* **43**(8), 1055–1072.

Martin, C. S., Vaz, S., Koubbi, P., Meaden, G. J., Engelhard, G. H., Lauria, V., Gardel, L., Coppin, F., Delavenne, J., Dupuis, L., Ernande, B., Foveau, A., Lelievre, S., Morin, J., Warembourg, C. & Carpentier, A. (2010). A digital atlas to link ontogenic shifts in fish spatial distribution to the environment of the eastern English Channel. Dab, *Limanda limanda* as a case-study. *Cybium* **34**(1), 59–71.

Martinho, F., Leitao, R., Neto, J. M., Cabral, H. N., Marques, J. C. & Pardal, M. A. (2007). The use of nursery areas by juvenile fish in a temperate estuary, Portugal. *Hydrobiologia* **587**, 281–290.

Martins, A. P. B., Heupel, M. R., Bierwagen, S. L., Chin, A. & Simpfendorfer, C. (2020). Diurnal activity patterns and habitat use of juvenile *Pastinachus ater* in a coral reef flat environment. *PLoS One* **15**(2), e0228280.

Martins, A. P. B., Heupel, M. R., Bierwagen, S. L., Chin, A. & Simpfendorfer, C. A. (2021). Tidal-diel patterns of movement, activity and habitat use by juvenile mangrove whiprays using towed-float GPS telemetry. *Marine and Freshwater Research* **72**(4), 534–541.

Mateo, I., Durbin, E., Appeldoorn, R., Adams, A., Juanes, F. & Durant, D. (2011*a*). Inferred growth of juvenile French grunts, *Haemulon flavolineatum*, and schoolmaster, *Lutjanus apodus*, in mangrove and seagrass habitats. *Bulletin of Marine Science* **87**(3), 339–350.

Mateo, I., Durbin, E. G., Bengtson, D. A. & Durant, D. (2011*b*). Variations in growth of tautog in nursery areas in Narragansett Bay and Rhode Island coastal ponds. *Marine and Coastal Fisheries* **3**(1), 271–278.

Mateo, I., Durbin, E. G., Bengtson, D. A., Kingsley, R., Swart, P. K. & Durant, D. (2010). Spatial and temporal variation in otolith chemistry for tautog (*Tautoga onitis*) in Narragansett Bay and Rhode Island coastal ponds. *Fishery Bulletin* **108**(2), 155–161.

Matich, P. & Heithaus, M. R. (2015). Individual variation in ontogenetic niche shifts in habitat use and movement patterns of a large estuarine predator (*Carcharhinus leucas*). *Oecologia* **178**(2), 347–359.

Matich, P., Nowicki, R. J., Davis, J., Mohan, J. A., Plumlee, J. D., Strickland, B. A., TinHan, T. C., Wells, R. J. D. & Fisher, M. (2020*a*). Does proximity to freshwater refuge affect the size structure of an estuarine predator (*Carcharhinus leucas*) in the north-western Gulf of Mexico? *Marine and Freshwater Research* **71**(11), 1501–1516.

Matich, P., Strickland, B. A. & Heithaus, M. R. (2020*b*). Long-term monitoring provides insight into estuarine top predator (*Carcharhinus leucas*) resilience following an extreme weather event. *Marine Ecology Progress Series* **639**, 169–183.

May-Ku, M. A., Criales, M. M., Montero-Munoz, J. L. & Ardisson, P. L. (2014). Differential use of *Thalassia testudinum* habitats by sympatric penaeids in a nursery ground of the southern Gulf of Mexico. *Journal of Crustacean Biology* **34**(2), 144–156.

Maynou, F., Lleonart, J. & Cartes, J. E. (2003). Seasonal and spatial variability of hake (*Merluccius merluccius* L.) recruitment in the NW Mediterranean. *Fisheries Research* **60**(1), 65–78.

Mbatha, F. L., Yemane, D., Ostrowski, M., Moloney, C. L. & Lipinski, M. R. (2019). Oxygen and temperature influence the distribution of deepwater Cape hake *Merluccius paradoxus* in the southern Benguela: a GAM analysis of a 10-year time-series. *African Journal of Marine Science* **41**(4), 413–427.

McAllister, J. D., Barnett, A., Lyle, J. M. & Semmens, J. M. (2015). Examining the functional role of current area closures used for the conservation of an overexploited and highly mobile fishery species. *ICES Journal of Marine Science* **72**(8), 2234–2244.

McAllister, J. D., Barnett, A., Lyle, J. M., Stehfest, K. M. & Semmens, J. M. (2018). Examining trends in abundance of an overexploited elasmobranch species in a nursery area closure. *Marine and Freshwater Research* **69**(3), 376–384.

McBride, R. S., MacDonald, T. C., Matheson, R. E., Rydene, D. A. & Hood, P. B. (2001). Nursery habitats for ladyfish, *Elops saurus*, along salinity gradients in two Florida estuaries. *Fishery Bulletin* **99**(3), 443–458.

McBride, R. S., Scherer, M. D. & Powell, J. C. (1995). Correlated variations in abundance, size, growth, and loss rates of age–0 bluefish in a southern New England estuary. *Transactions of the American Fisheries Society* **124**(6), 898–910.

McBride, R. S., Tweedie, M. K. & Oliveira, K. (2018). Reproduction, first-year growth, and expansion of spawning and nursery grounds of black sea bass (*Centropristis striata*) into a warming Gulf of Maine. *Fishery Bulletin* **116**(3–4), 323–336.

McCallister, M., Ford, R. & Gelsleichter, J. (2013). Abundance and distribution of sharks in northeast Florida waters and identification of potential nursery habitat. *Marine and Coastal Fisheries* **5**(1), 200–210.

McCormick, M. I. & Hoey, A. S. (2004). Larval growth history determines juvenile growth and survival in a tropical marine fish. *Oikos* **106**(2), 225–242.

McMahon, K. W., Berumen, M. L., Mateo, I., Elsdon, T. S. & Thorrold, S. R. (2011). Carbon isotopes in otolith amino acids identify residency of juvenile snapper (Family: Lutjanidae) in coastal nurseries. *Coral Reefs* **30**(4), 1135–1145.

McMahon, K. W., Berumen, M. L. & Thorrold, S. R. (2012). Linking habitat mosaics and connectivity in a coral reef seascape. *Proceedings of the National Academy of Sciences of the United States of America* **109**(38), 15372–15376.

McMillan, R. O., Armstrong, D. A. & Dinnel, P. A. (1995). Comparison of intertidal habitat use and growth rates of two northern Puget Sound cohorts of 0+ age dungeness crab, *Cancer magister*. *Estuaries* **18**(2), 390–398.

McNeill, S. E., Worthington, D. G., Ferrell, D. J. & Bell, J. D. (1992). Consistently outstanding recruitment of five species of fish to a seagrass bed in Botany Bay, NSW. *Australian Journal of Ecology* **17**(4), 359–365.

Meakin, C. A. & Qin, J. G. (2020). Evaluation of food competition and resource partitioning of recruiting fish with permanent residents in a seagrass habitat. *New Zealand Journal of Marine and Freshwater Research* **54**(2), 149–166.

Meise, C. J. & Stehlik, L. L. (2003). Habitat use, temporal abundance variability, and diet of blue crabs from a New Jersey estuarine system. *Estuaries* **26**(3), 731–745.

Mendes, C., Ramos, S., Elliott, M. & Bordalo, A. A. (2020). Feeding strategies and body condition of juvenile European flounder *Platichthys flesus* in a nursery habitat. *Journal of the Marine Biological Association of the United Kingdom* **100**(5), 795–806.

Mendo, T., Lyle, J. M., Moltschaniwskyj, N. A. & Semmens, J. M. (2015). Early post-settlement mortality of the scallop *Pecten fumatus* and the role of algal mats as a refuge from predation. *ICES Journal of Marine Science* **72**(8), 2322–2331.

Mendonca, V., Flores, A. A. V., Silva, A. C. F. & Vinagre, C. (2019). Do marine fish juveniles use intertidal tide pools as feeding grounds? *Estuarine Coastal and Shelf Science* **225**, 106255.

Meng, L., Cicchetti, G. & Raciti, S. (2005). Relationships between juvenile winter flounder and multiple-scale habitat variation in Narragansett Bay, Rhode Island. *Transactions of the American Fisheries Society* **134**(6), 1509–1519.

Mensink, P. J. & Shima, J. S. (2014). Patterns of co-occurrence and interactions between age classes of the common triplefin, *Forsterygion lapillum*. *Marine Biology* **161**(6), 1285–1298.

Merson, R. R. & Pratt, H. L. (2001). Distribution, movements and growth of young sandbar sharks, *Carcharhinus plumbeus*, in the nursery grounds of Delaware Bay. *Environmental Biology of Fishes* **61**(1), 13–24.

Methven, D. A. & Schneider, D. C. (1998). Gear-independent patterns of variation in catch of juvenile Atlantic cod (*Gadus morhua*) in coastal habitats. *Canadian Journal of Fisheries and Aquatic Sciences* **55**(6), 1430–1442.

Mikulas, J. J. & Rooker, J. R. (2008). Habitat use, growth, and mortality of post-settlement lane snapper (*Lutjanus synagris*) on natural banks in the northwestern Gulf of Mexico. *Fisheries Research* **93**(1–2), 77–84.

Miltner, R. J., Ross, S. W. & Posey, M. H. (1995). Influence of food and predation on the depth distribution of juvenile spot (*Leiostomus xanthurus*) in tidal nurseries. *Canadian Journal of Fisheries and Aquatic Sciences* **52**(5), 971–982.

Minello, T. J. (1998). Nekton densities in shallow estuarine habitats of Texas and Louisiana and the identification of essential fish habitat. In L. R. Benaka (Ed.), *Fish Habitat: Essential Fish Habitat and Rehabilitation* (Vol. 22, pp. 43–75). Bethesda: Amer Fisheries Soc.

Mirera, D. O. (2017). Intertidal mangrove boundary zones as nursery grounds for the mud crab *Scylla serrata*. *African Journal of Marine Science* **39**(3), 315–325.

Mislan, K. A. S. & Babcock, R. C. (2008). Survival and behaviour of juvenile red rock lobster, *Jasus edwardsii*, on rocky reefs with varying predation pressure and habitat complexity. *Marine and Freshwater Research* **59**(3), 246–253.

Mohan, J. A., Halden, N. M. & Rulifson, R. A. (2015). Habitat use of juvenile striped bass *Morone saxatilis* (Actinopterygii: Moronidae) in rivers spanning a salinity gradient across a shallow wind-driven estuary. *Environmental Biology of Fishes* **98**(4), 1105–1116.

Mohan, J. A. & Walther, B. D. (2018). Integrating multiple natural tags to link migration patterns and resource partitioning across a subtropical estuarine gradient. *Estuaries and Coasts* **41**(6), 1806–1820.

Moksnes, P. O. (2002). The relative importance of habitat-specific settlement, predation and juvenile dispersal for distribution and abundance of young juvenile shore crabs *Carcinus maenas* L. *Journal of Experimental Marine Biology and Ecology* **271**(1), 41–73.

Moksnes, P. O. & Heck, K. L. (2006). Relative importance of habitat selection and predation for the distribution of blue crab megalopae and young juveniles. *Marine Ecology Progress Series* **308**, 165–181.

Moksnes, P. O., Hedvall, O. & Reinwald, T. (2003). Settlement behavior in shore crabs *Carcinus maenas*: why do postlarvae emigrate from nursery habitats? *Marine Ecology Progress Series* **250**, 215–230.

Moksnes, P. O., Pihl, L. & van Montfrans, J. (1998). Predation on postlarvae and juveniles of the shore crab *Carcinus maenas*: importance of shelter, size and cannibalism. *Marine Ecology Progress Series* **166**, 211–225.

Moraes, M. C. M. & Lavrado, H. P. (2017). Distribution of loliginid squids in a eutrophicated tropical coastal bay. *Marine Biology Research* **13**(3), 330–341.

Morais, P., Dias, E., Babaluk, J. & Antunes, C. (2011). The migration patterns of the European flounder *Platichthys flesus* (Linnaeus, 1758) (Pleuronectidae, Pisces) at the southern limit of its distribution range: Ecological implications and fishery management. *Journal of Sea Research* **65**(2), 235–246.

Morat, F., Letourneur, Y., Dierking, J., Pecheyran, C., Bareille, G., Blamart, D. & Harmelin-Vivien, M. (2014). The great melting pot. Common sole population connectivity assessed by otolith and water fingerprints. *PLoS One* **9**(1), e86585.

Moreno, A., Lourenco, S., Pereira, J., Gaspar, M. B., Cabral, H. N., Pierce, G. J. & Santos, A. M. P. (2014). Essential habitats for pre-recruit *Octopus vulgaris* along the Portuguese coast. *Fisheries Research* **152**, 74–85.

Morgan, D. L., Ebner, B. C., Allen, M. G., Gleiss, A. C., Beatty, S. J. & Whitty, J. M. (2017). Habitat use and site fidelity of neonate and juvenile green sawfish *Pristis zijsron* in a nursery area in Western Australia. *Endangered Species Research* **34**, 235–249.

Morgan, D. L., Lear, K. O., Dobinson, E., Gleiss, A. C., Fazeldean, T., Pillans, R. D., Beatty, S. J. & Whitty, J. M. (2021). Seasonal use of a macrotidal estuary by the endangered dwarf sawfish, *Pristis clavata*. *Aquatic Conservation-Marine and Freshwater Ecosystems* **31**, 2164–2177.

Morgan, S. G., ZimmerFaust, R. K., Heck, K. L. & Coen, L. D. (1996). Population regulation of blue crabs *Callinectes sapidus* in the northern Gulf of Mexico: Postlarval supply. *Marine Ecology Progress Series* **133**(1–3), 73–88.

Morton, D. N. & Shima, J. S. (2013). Habitat configuration and availability influences the settlement of temperate reef fishes (Tripterygiidae). *Journal of Experimental Marine Biology and Ecology* **449**, 215–220.

Morton, R. M., Halliday, I. & Cameron, D. (1993). Movement of tagged juvenile tailor (*Pomatomus saltatrix*) in Moreton Bay, Queensland. *Australian Journal of Marine and Freshwater Research* **44**(6), 811–816.

Moura, R. L., Francini, R. B., Chaves, E. M., Minte-Vera, C. V. & Lindeman, K. C. (2011). Use of riverine through reef habitat systems by dog snapper (*Lutjanus jocu*) in eastern Brazil. *Estuarine Coastal and Shelf Science* **95**(1), 274–278.

Muller, C. & Strydom, N. A. (2017). Evidence for habitat residency and isotopic niche partitioning in a marine-estuarine-dependent species associated with mangrove habitats from the east coast of South Africa. *Estuaries and Coasts* **40**(6), 1642–1652.

Munnelly, R. T., Reeves, D. B., Chesney, E. J. & Baltz, D. M. (2021). Spatial and temporal influences of nearshore hydrography on fish assemblages associated with energy platforms in the northern Gulf of Mexico. *Estuaries and Coasts* **44**(1), 269–285.

Munroe, S. E. M., Simpfendorfer, C. A. & Heupel, M. R. (2016). Variation in blacktip shark movement patterns in a tropical coastal bay. *Environmental Biology of Fishes* **99**(4), 377–389.

Murase, A., Ishimaru, T., Ogata, Y., Yamasaki, Y., Kawano, H., Nakanishi, K. & Inoue, K. (2020). Where is the nursery for amphidromous nekton? Abundance and size comparisons of juvenile ayu among habitats and contexts. *Estuarine Coastal and Shelf Science* **241**, 106831.

Murchie, K. J., Schwager, E., Cooke, S. J., Danylchuk, A. J., Danylchuk, S. E., Goldberg, T. L., Suski, C. D. & Philipp, D. P. (2010). Spatial ecology of juvenile lemon sharks (*Negaprion brevirostris*) in tidal creeks and coastal waters of Eleuthera, The Bahamas. *Environmental Biology of Fishes* **89**(1), 95–104.

Nack, C. C., Limburg, K. E. & Miller, D. (2015). Assessing the quality of four inshore habitats used by post yolk-sac *Alosa sapidissima* (Wilson 1811) in the Hudson River: a prelude to restoration. *Restoration Ecology* **23**(1), 57–64.

Nagelkerken, I., Huebert, K. B., Serafy, J. E., Grol, M. G. G., Dorenbosch, M. & Bradshaw, C. J. A. (2017). Highly localized replenishment of coral reef fish populations near nursery habitats. *Marine Ecology Progress Series* **568**, 137–150.

Nagelkerken, I., Roberts, C. M., van der Velde, G., Dorenbosch, M., van Riel, M. C., de la Morinere, E. C. & Nienhuis, P. H. (2002). How important are mangroves and seagrass beds for coral-reef fish? The nursery hypothesis tested on an island scale. *Marine Ecology Progress Series* **244**, 299–305.

Nakamura, Y., Hirota, K., Shibuno, T. & Watanabe, Y. (2012). Variability in nursery function of tropical seagrass beds during fish ontogeny: timing of ontogenetic habitat shift. *Marine Biology* **159**(6), 1305–1315.

Nakamura, Y., Horinouchi, M., Sano, M. & Shibuno, T. (2009). The effects of distance from coral reefs on seagrass nursery use by 5 emperor fishes at the southern Ryukyu Islands, Japan. *Fisheries Science* **75**(6), 1401–1408.

Nakamura, Y., Horinouchi, M., Shibuno, T., Tanaka, Y., Miyajima, T., Koike, I., Kurokura, H. & Sano, M. (2008). Evidence of ontogenetic migration from mangroves to coral reefs by black-tail snapper *Lutjanus fulvus*: stable isotope approach. *Marine Ecology Progress Series* **355**, 257–266.

Nakata, H., Kimura, S., Okazaki, Y. & Kasai, A. (2000). Implications of meso-scale eddies caused by frontal disturbances of the Kuroshio Current for anchovy recruitment. *ICES Journal of Marine Science* **57**(1), 143–151.

Nakaya, M., Takatsu, T., Joh, M., Nakagami, M. & Takahashi, T. (2007). Annual variation of potential predation impacts on larval and juvenile marbled sole *Pseudopleuronectes yokohamae* by sand shrimp *Crangon uritai* in Hakodate Bay, Hokkaido. *Fisheries Science* **73**(1), 112–122.

Nanami, A. & Endo, T. (2007). Seasonal dynamics of fish assemblage structures in a surf zone on an exposed sandy beach in Japan. *Ichthyological Research* **54**(3), 277–286.

Nanez-James, S. E., Stunz, G. W. & Holt, S. A. (2009). Habitat use patterns of newly settled southern flounder, *Paralichthys lethostigma*, in Aransas-Copano Bay, Texas. *Estuaries and Coasts* **32**(2), 350–359.

Nanjo, K., Nakamura, Y., Horinouchi, M., Kohno, H. & Sano, M. (2011). Predation risks for juvenile fishes in a mangrove estuary: A comparison of vegetated and unvegetated microhabitats by tethering experiments. *Journal of Experimental Marine Biology and Ecology* **405**(1–2), 53–58.

Nash, R. D. M. & Geffen, A. J. (2000). The influence of nursery ground processes in the determination of year-class strength in juvenile plaice *Pleuronectes platessa* L. in Port Erin Bay, Irish Sea. *Journal of Sea Research* **44**(1–2), 101–110.

Nash, R. D. M., Geffen, A. J., Burrows, M. T. & Gibson, R. N. (2007). Dynamics of shallow-water juvenile flatfish nursery grounds: application of the self-thinning rule. *Marine Ecology Progress Series* **344**, 231–244.

Neahr, T. A., Stunz, G. W. & Minello, T. J. (2010). Habitat use patterns of newly settled spotted seatrout in estuaries of the north-western Gulf of Mexico. *Fisheries Management and Ecology* **17**(5), 404–413.

Necaise, A. M. D., Ross, S. W. & Miller, J. M. (2005). Estuarine habitat evaluation measured by growth of juvenile summer flounder *Paralichthys dentatus* in a North Carolina estuary. *Marine Ecology Progress Series* **285**, 157–168.

Nellis, P., Munro, J., Hatin, D., Desrosiers, G., Simons, R. D. & Guilbard, F. (2007). Macrobenthos assemblages in the St. Lawrence estuarine transition zone and their potential as food for Atlantic sturgeon and lake sturgeon. In J. Munro (Ed.), *Anadromous Sturgeons: Habitats, Threats, and Management* (Vol. 56, pp. 105-128). Bethesda: Amer Fisheries Soc.

Nelson, T. R., Hightower, C. L., Coogan, J., Walther, B. D. & Powers, S. P. (2021). Patterns and consequences of life history diversity in salinity exposure of an estuarine dependent fish. *Environmental Biology of Fishes* **104**(4), 419–436.

Nemerson, D. M. & Able, K. W. (2004). Spatial patterns in diet and distribution of juveniles of four fish species in Delaware Bay marsh creeks: factors influencing fish abundance. *Marine Ecology Progress Series* **276**, 249–262.

Nestlerode, J. A., Luckenbach, M. W. & O'Beirn, F. X. (2007). Settlement and survival of the oyster *Crassostrea virginica* on created oyster reef habitats in Chesapeake Bay. *Restoration Ecology* **15**(2), 273–283.

Newhard, J. J., Love, J. W. & Gill, J. (2012). Do juvenile white perch *Morone americana* grow better in freshwater habitats of the Blackwater River drainage (Chesapeake Bay, MD, USA)? *Estuaries and Coasts* **35**(4), 1110–1118.

Nicolas, D., Le Loc'h, F., Desaunay, Y., Hamon, D., Blanchet, A. & Le Pape, O. (2007). Relationships between benthic macrofauna and habitat suitability for juvenile common sole (*Solea solea*, L.) in the Vilaine estuary (Bay of Biscay, France) nursery ground. *Estuarine Coastal and Shelf Science* **73**(3–4), 639–650.

Niklitschek, E. J., Secor, D. H., Toledo, P., Valenzuela, X., Cubillos, L. A. & Zuleta, A. (2014). Nursery systems for Patagonian grenadier off Western Patagonia: large inner sea or narrow continental shelf? *ICES Journal of Marine Science* **71**(2), 374–390.

Nissling, A. & Wallin, I. (2020). Recruitment variability in Baltic flounder (*Platichthys solemdali*) – effects of salinity with implications for stock development facing climate change. *Journal of Sea Research* **162**, 101913.

Norcross, B. L., Holladay, B. A. & Muter, F. J. (1995). Nursery area characteristics of Pleuronectids in coastal Alaska, USA. *Netherlands Journal of Sea Research* **34**(1–3), 161–175.

Norcross, B. L., Muter, F. J. & Holladay, B. A. (1997). Habitat models for juvenile pleuronectids around Kodiak Island, Alaska. *Fishery Bulletin* **95**(3), 504–520.

North, E. W. & Houde, E. D. (2001). Retention of white perch and striped bass larvae: Biological-physical interactions in Chesapeake Bay estuarine turbidity maximum. *Estuaries* **24**(5), 756–769.

Norton, S. L., Wiley, T. R., Carlson, J. K., Frick, A. L., Poulakis, G. R. & Simpfendorfer, C. A. (2012). Designating critical habitat for juvenile endangered smalltooth sawfish in the United States. *Marine and Coastal Fisheries* **4**(1), 473–480.

Nosal, A. P., Cartamil, D. P., Wegner, N. C., Lam, C. H. & Hastings, P. A. (2019). Movement ecology of young-of-the-year blue sharks *Prionace glauca* and shortfin makos *Isurus oxyrinchus* within a putative binational nursery area. *Marine Ecology Progress Series* **623**, 99–115.

Nys, L. N., Fabrizio, M. C. & Tuckey, T. D. (2015). Multi-decadal variation in size of juvenile Summer Flounder (*Paralichthys dentatus*) in Chesapeake Bay. *Journal of Sea Research* **103**, 50–58.

Nys, L. N., Fabrizio, M. C. & Tuckey, T. D. (2016). Multi-decadal variation in size of juvenile Summer Flounder (*Paralichthys dentatus*) in Chesapeake Bay. *Journal of Sea Research* **107**, 112–120.

O'Brien, C. J. (1994). Population dynamics of juvenile tiger prawns *Penaeus esculentus* in South Queensland, Australia. *Marine Ecology Progress Series* **104**(3), 247–256.

Oh, B. Z. L., Thums, M., Babcock, R. C., Meeuwig, J. J., Pillans, R. D., Speed, C. & Meekan, M. G. (2017). Contrasting patterns of residency and space use of coastal sharks within a communal shark nursery. *Marine and Freshwater Research* **68**(8), 1501–1517.

Olsen, Z. (2019). Quantifying nursery habitat function: variation in habitat suitability linked to mortality and growth for juvenile black drum in a hypersaline estuary. *Marine and Coastal Fisheries* **11**(1), 86–96.

Olson, A. M., Hessing-Lewis, M., Haggarty, D. & Juanes, F. (2019). Nearshore seascape connectivity enhances seagrass meadow nursery function. *Ecological Applications* **29**(5), e01897.

Onate-Gonzalez, E. C., Sosa-Nishizaki, O., Herzka, S. Z., Lowe, C. G., Lyons, K., Santana-Morales, O., Sepulveda, C., Guerrero-Avila, C., Garcia-Rodriguez, E. & O'Sullivan, J. B. (2017). Importance of Bahia Sebastian Vizcaino as a nursery area for white sharks (*Carcharodon carcharias*) in the Northeastern Pacific: A fishery dependent analysis. *Fisheries Research* **188**, 125–137.

Oshima, M., Robert, D., Kurita, Y., Yoneda, M., Tominaga, O., Tomiyama, T., Yamashita, Y. & Uehara, S. (2010). Do early growth dynamics explain recruitment success in Japanese flounder *Paralichthys olivaceus* off the Pacific coast of northern Japan? *Journal of Sea Research* **64**(1–2), 93–100.

Ota, N., Kawai, T. & Hashimoto, A. (2013). Recruitment, growth, and vertical distribution of the endangered mud snail *Cerithidea rhizophorarum* A. Adams, 1855: implications for its conservation. *Molluscan Research* **33**(2), 87–97.

Paillon, C., Wantiez, L., Kulbicki, M., Labonne, M. & Vigliola, L. (2014). Etent of mangrove nursery habitats determines the geographic distribution of a coral reef fish in a south-pacific archipelago. *PLoS One* **9**(8), e105158.

Palma, A. T., Pardo, L. M., Veas, R., Cartes, C., Silva, M., Manriquez, K., Diaz, A., Munoz, C. & Ojeda, F. P. (2006). Coastal brachyuran decapods: settlement and recruitment under contrasting coastal geometry conditions. *Marine Ecology Progress Series* **316**, 139–153.

Palma, A. T., Wahle, R. A. & Steneck, R. S. (1998). Different early post-settlement strategies between American lobsters *Homarus americanus* and rock crabs *Cancer irroratus* in the Gulf of Maine. *Marine Ecology Progress Series* **162**, 215–225.

Paperno, R., Targett, T. E. & Grecay, P. A. (2000). Spatial and temporal variation in recent growth, overall growth, and mortality of juvenile weakfish (*Cynoscion regalis*) in Delaware Bay. *Estuaries* **23**(1), 10–20.

Pardieck, R. A., Orth, R. J., Diaz, R. J. & Lipcius, R. N. (1999). Ontogenetic changes in habitat use by postlarvae and young juveniles of the blue crab. *Marine Ecology Progress Series* **186**, 227–238.

Pardo, L. M., Rubilar, P. S. & Fuentes, J. P. (2020). North Patagonian estuaries appear to function as nursery habitats for marble crab (*Metacarcinus edwardsii*). *Regional Studies in Marine Science* **36**, 101315.

Parrish, F. A., DeMartini, E. E. & Ellis, D. M. (1997). Nursery habitat in relation to production of juvenile pink snapper, *Pristipomoides filamentosus*, in the Hawaiian Archipelago. *Fishery Bulletin* **95**(1), 137–148.

Parsons, D. M., Buckthought, D., Edhouse, S. & Lohrer, A. M. (2020). The paradox of the Hauraki Gulf snapper population: Testing the nursery habitat concept. *Marine Ecology-an Evolutionary Perspective* **41**(2), e12582.

Parsons, D. M., Buckthought, D., Middleton, C. & MacKay, G. (2016). Relative abundance of snapper (*Chrysophrys auratus*) across habitats within an estuarine system. *New Zealand Journal of Marine and Freshwater Research* **50**(3), 358–370.

Parsons, D. M., MacDonald, I., Buckthought, D. & Middleton, C. (2018). Do nursery habitats provide shelter from flow for juvenile fish? *PLoS One* **13**(1), e0186889.

Parsons, D. M., Middleton, C., Smith, M. D. & Cole, R. G. (2014). The influence of habitat availability on juvenile fish abundance in a northeastern New Zealand estuary. *New Zealand Journal of Marine and Freshwater Research* **48**(2), 216–228.

Parsons, D. M., Middleton, C., Spong, K. T., Mackay, G., Smith, M. D. & Buckthought, D. (2015). Mechanisms explaining nursery habitat association: how do juvenile snapper (*Chrysophrys auratus*) benefit from their nursery habitat? *PLoS One* **10**(3), e0122137.

Parsons, D. M., Morrison, M. A., Thrush, S. F., Middleton, C., Smith, M., Spong, K. T. & Buckthought, D. (2013). The influence of habitat structure on juvenile fish in a New Zealand estuary. *Marine Ecology-an Evolutionary Perspective* **34**(4), 492–500.

Pasquaud, S., Beguer, M., Larsen, M. H., Chaalali, A., Cabral, H. & Lobry, J. (2012). Increase of marine juvenile fish abundances in the middle Gironde estuary related to warmer and more saline waters, due to global changes. *Estuarine Coastal and Shelf Science* **104**, 46–53.

Pastoors, M. A., Rijnsdorp, A. D. & Van Beek, F. A. (2000). Effects of a partially closed area in the North Sea ("plaice box") on stock development of plaice. *ICES Journal of Marine Science* **57**(4), 1014–1022.

Pastor, J., Koeck, B., Astruch, P. & Lenfant, P. (2013). Coastal man-made habitats: Potential nurseries for an exploited fish species, *Diplodus sargus* (Linnaeus, 1758). *Fisheries Research* **148**, 74–80.

Pawson, M. G. & Eaton, D. R. (1999). The influence of a power station on the survival of juvenile sea bass in an estuarine nursery area. *Journal of Fish Biology* **54**(6), 1143–1160.

Pellizzato, M., Galvan, T., Lazzarini, R. & Penzo, P. (2011). Recruitment of *Tapes philippinarum* in the Venice Lagoon (Italy) during 2002–2007. *Aquaculture International* **19**(3), 541–554.

Pennino, M. G., Vilela, R., Bellido, J. M. & Velasco, F. (2019). Balancing resource protection and fishing activity: The case of the European hake in the northern Iberian Peninsula. *Fisheries Oceanography* **28**(1), 54–65.

Perez-Castaneda, R. & Defeo, O. (2004). Spatial distribution and structure along ecological gradients: penaeid shrimps in a tropical estuarine habitat of Mexico. *Marine Ecology Progress Series* **273**, 173–185.

Perkins-Visser, E., Wolcott, T. G. & Wolcott, D. L. (1996). Nursery role of seagrass beds: Enhanced growth of juvenile blue crabs (*Callinectes sapidus* Rathbun). *Journal of Experimental Marine Biology and Ecology* **198**(2), 155–173.

Petrie, M. E. & Ryer, C. H. (2006). Laboratory and field evidence for structural habitat affinity of young-of-the-year lingcod. *Transactions of the American Fisheries Society* **135**(6), 1622–1630.

Pickett, G. D., Kelley, D. F. & Pawson, M. G. (2004). The patterns of recruitment of sea bass, *Dicentrarchus labrax* L. from nursery areas in England and Wales and implications for fisheries management. *Fisheries Research* **68**(1–3), 329–342.

Pihl, L., Modin, J. & Wennhage, H. (2000). Spatial distribution patterns of newly settled plaice (*Pleuronectes platessa* L.) along the Swedish Skagerrak archipelago. *Journal of Sea Research* **44**(1–2), 65–80.

Pihl, L. & van der Veer, H. W. (1992). Importance of exposure and habitat structure for the population density of 0-group plaice, *Pleuronectes platessa* L, in coastal nursery areas. *Netherlands Journal of Sea Research* **29**(1–3), 145–152.

Pile, A. J., Lipcius, R. N., VanMontfrans, J. & Orth, R. J. (1996). Density-dependent settler-recruit-juvenile relationships in blue crabs. *Ecological Monographs* **66**(3), 277–300.

Pillans, R. D., Fry, G. C., Steven, A. D. L. & Patterson, T. (2020). Environmental influences on long-term movement patterns of a euryhaline elasmobranch (*Carcharhinus leucas*) within a subtropical estuary. *Estuaries and Coasts* **43**(8), 2152–2169.

Pimentel, C. R. & Joyeux, J. C. (2010). Diet and food partitioning between juveniles of mutton *Lutjanus analis*, dog *Lutjanus jocu* and lane *Lutjanus synagris* snappers (Perciformes: Lutjanidae) in a mangrove-fringed estuarine environment. *Journal of Fish Biology* **76**(10), 2299–2317.

Pimiento, C., Nifong, J. C., Hunter, M. E., Monaco, E. & Silliman, B. R. (2015). Habitat use patterns of the invasive red lionfish *Pterois volitans*: a comparison between mangrove and reef systems in San Salvador, Bahamas. *Marine Ecology-an Evolutionary Perspective* **36**(1), 28–37.

Pirtle, J. L., Eckert, G. L. & Stoner, A. W. (2012). Habitat structure influences the survival and predator-prey interactions of early juvenile red king crab *Paralithodes camtschaticus*. *Marine Ecology Progress Series* **465**, 169–184.

Pirtle, J. L., Shotwell, S. K., Zimmerman, M., Reid, J. A. & Golden, N. (2019). Habitat suitability models for groundfish in the Gulf of Alaska. *Deep-Sea Research Part II-Topical Studies in Oceanography* **165**, 303–321.

Plaza, G., Ishida, M. & Aoyama, D. (2008). Temporal patterns of growth in larval cohorts of the Japanese sardine *Sardinops melanostictus* in a coastal nursery area. *Journal of Fish Biology* **73**(6), 1284–1300.

Plaza, G., Katayama, S. & Omori, M. (2002). Abundance and early life history traits of young-of-the-year *Sebastes inermis* in a Zostera marina bed. *Fisheries Science* **68**(6), 1254–1264.

Plaza, G., Katayama, S. & Omori, M. (2010). Daily patterns of settlement and individual growth rates of young-of-the-year of the rockfish *Sebastes inermis* in a *Sargassum* bed. *Fisheries Research* **103**(1–3), 48–55.

Poiesz, S. S. H., van Leeuwen, A., Soetaert, K., Witte, J. I. J., Zaat, D. S. C. & van der Veer, H. W. (2020). Is summer growth reduction related to feeding guild? A test for a benthic juvenile flatfish sole (*Solea solea*) in a temperate coastal area, the western Wadden Sea. *Estuarine Coastal and Shelf Science* **235**, 106570.

Polte, P. & Asmus, H. (2006). Influence of seagrass beds (*Zostera noltii*) on the species composition of juvenile fishes temporarily visiting the intertidal zone of the Wadden Sea. *Journal of Sea Research* **55**(3), 244–252.

Poos, J. J., Aarts, G., Vandemaele, S., Willems, W., Bolle, L. J. & van Helmond, A. T. M. (2013). Estimating spatial and temporal variability of juvenile North Sea plaice from opportunistic data. *Journal of Sea Research* **75**, 118–128.

Posey, M. H., Alphin, T. D., Harwell, H. & Allen, B. (2005). Importance of low salinity areas for juvenile blue crabs, *Callinectes sapidus* Rathbun, in river-dominated estuaries of southeastern United States. *Journal of Experimental Marine Biology and Ecology* **319**(1–2), 81–100.

Potthoff, M. T. & Allen, D. M. (2003). Site fidelity, home range, and tidal migrations of juvenile pinfish, *Lagodon rhomboides*, in salt marsh creeks. *Environmental Biology of Fishes* **67**(3), 231–240.

Poulakis, G. R., Stevens, P. W., Timmers, A. A., Wiley, T. R. & Simpfendorfer, C. A. (2011). Abiotic affinities and spatiotemporal distribution of the endangered smalltooth sawfish, *Pristis pectinata*, in a south-western Florida nursery. *Marine and Freshwater Research* **62**(10), 1165–1177.

Primo, A. L., Azeiteiro, U. M., Marques, S. C., Martinho, F., Baptista, J. & Pardal, M. A. (2013). Colonization and nursery habitat use patterns of larval and juvenile flatfish species in a small temperate estuary. *Journal of Sea Research* **76**, 126–134.

Primo, A. L., Vaz, A. C., Crespo, D., Costa, F., Pardal, M. & Martinho, F. (2021). Contrasting links between growth and survival in the early life stages of two flatfish species. *Estuarine Coastal and Shelf Science* **254**, 107314.

Pruell, R. J., Taplin, B. K. & Karr, J. D. (2012). Spatial and temporal trends in stable carbon and oxygen isotope ratios of juvenile winter flounder otoliths. *Environmental Biology of Fishes* **93**(1), 61–71.

Rabaut, M., Calderon, M. A., van de Moortel, L., van Dalfsen, J., Vincx, M., Degraer, S. & Desroy, N. (2013). The role of structuring benthos for juvenile flatfish. *Journal of Sea Research* **84**, 70–76.

Rabaut, M., Van de Moortel, L., Vincx, M. & Degraer, S. (2010). Biogenic reefs as structuring factor in *Pleuronectes platessa* (Plaice) nursery. *Journal of Sea Research* **64**(1–2), 102–106.

Rackovan, J. L. & Howell, W. H. (2017). Spatial and temporal distribution of juvenile *Cyclopterus lumpus* (lumpfish) in a New England estuary. *Regional Studies in Marine Science* **16**, 109–115.

Rakocinski, C. F. & McCall, D. D. (2005). Early blue crab recruitment to alternative nursery habitats in Mississippi, USA. *Journal of Shellfish Research* **24**(1), 253–259.

Ralph, G. M., Seitz, R. D., Orth, R. J., Knick, K. E. & Lipcius, R. N. (2013). Broad-scale association between seagrass cover and juvenile blue crab density in Chesapeake Bay. *Marine Ecology Progress Series* **488**, 51–63.

Ramos, J. A. A., Barletta, M., Dantas, D. V. & Costa, M. F. (2016). Seasonal and spatial ontogenetic movements of Gerreidae in a Brazilian tropical estuarine ecocline and its application for nursery habitat conservation. *Journal of Fish Biology* **89**(1), 696–712.

Ramos, S., Re, P. & Bordalo, A. A. (2009). Environmental control on early life stages of flatfishes in the Lima Estuary (NW Portugal). *Estuarine Coastal and Shelf Science* **83**(2), 252–264.

Rangeley, R. W. & Kramer, D. L. (1995*a*). Tidal effects on habitat selection and aggregation by juvenile pollock *Pollachius virens* in the rocky intertidal zone. *Marine Ecology Progress Series* **126**(1–3), 19–29.

Rangeley, R. W. & Kramer, D. L. (1995*b*). Use of rocky intertidal habitats by juvenile pollock *Pollachius virens*. *Marine Ecology Progress Series* **126**(1–3), 9–17.

Ray, M. & Stoner, A. W. (1994). Experimental analysis of growth and survivorship in a marine gastropod aggregation - balancing growth with safety in numbers. *Marine Ecology Progress Series* **105**(1–2), 47–59.

Ray, M. & Stoner, A. W. (1995). Growth, survivorship, and habitat choice in a newly settled seagrass gastropod, *Strombus gigas*. *Marine Ecology Progress Series* **123**(1–3), 83–94.

Rechisky, E. L. & Wetherbee, B. M. (2003). Short-term movements of juvenile and neonate sandbar sharks, *Carcharhinus plumbeus*, on their nursery grounds in Delaware Bay. *Environmental Biology of Fishes* **68**(2), 113–128.

Reichert, M. J. M. & Vanderveer, H. W. (1991). Settlement, abundance, growth and mortality of juvenile flatfish in a subtropical tidal estuary (Georgia, USA). *Netherlands Journal of Sea Research* **27**(3–4), 375–391.

Reis-Santos, P., Tanner, S. E., Vasconcelos, R. P., Elsdon, T. S., Cabral, H. N. & Gillanders, B. M. (2013). Connectivity between estuarine and coastal fish populations: contributions of estuaries are not consistent over time. *Marine Ecology Progress Series* **491**, 177–186.

Relini, L. O., Papaconstantinou, C., Jukic-Peladic, S., Souplet, A., De Sola, L. G., Piccinetti, C., Kavadas, S. & Rossi, M. (2002). Distribution of the Mediterranean hake populations (*Merluccius merluccius smiridus* Rafinesque, 1810) (Osteichthyes : Gadiformes) based on six years monitoring by trawl-surveys: some implications for management. *Scientia Marina* **66**, 21–38.

Renan, X., Cervera-Cervera, K. & Brule, T. (2003). *Probable nursery areas for juvenile groupers along the northern coast of the Yucatan Peninsula, Mexico* (Vol. 54). Ft Pierce: Proceedings of the Gulf and Caribbean Fisheries Institute.

Renkawitz, M. D., Gregory, R. S. & Schneider, D. C. (2011). Habitat dependant growth of three species of bottom settling fish in a coastal fjord. *Journal of Experimental Marine Biology and Ecology* **409**(1–2), 79–88.

Riedel, R., Perry, H., Warren, J., Criss, A. & van Devender, T. (2008). Using conventional analysis in parallel with Geographical Information Systems techniques to examine the distribution of brown shrimp in the Mississippi Sound. *North American Journal of Fisheries Management* **28**(5), 1439–1449.

Riou, P., Le Pape, O. & Rogers, S. I. (2001). Relative contributions of different sole and plaice nurseries to the adult population in the Eastern Channel: application of a combined method using generalized linear models and a geographic information system. *Aquatic Living Resources* **14**(2), 125–135.

Rishworth, G. M., Strydom, N. A. & Potts, W. M. (2015). The nursery role of a sheltered surf-zone in warm-temperate southern Africa. *African Zoology* **50**(1), 11–16.

Rodrigues, M. A., Ortega, I. & D'Incao, F. (2019). The importance of shallow areas as nursery grounds for the recruitment of blue crab (*Callinectes sapidus*) juveniles in subtropical estuaries of Southern Brazil. *Regional Studies in Marine Science* **25**, 100492.

Rodriguez-Climent, S., Angelico, M. M., Marques, V., Oliveira, P., Wise, L. & Silva, A. (2017). Essential habitat for sardine juveniles in Iberian waters. *Scientia Marina* **81**(3), 351–360.

Rogers-Bennett, L. & Pearse, J. S. (2001). Indirect benefits of marine protected areas for juvenile abalone. *Conservation Biology* **15**(3), 642–647.

Rogers, S. I. (1992). Environmental factors affecting the distribution of sole (*Solea solea* (L)) within a nursery area. *Netherlands Journal of Sea Research* **29**(1–3), 153–161.

Rogers, S. I. (1994). Population density and growth rate of juvenile sole *Solea solea* (L). *Netherlands Journal of Sea Research* **32**(3–4), 353–360.

Rohtla, M. & Vetemaa, M. (2016). Otolith chemistry chimes in: migratory environmental histories of Atlantic tarpon (*Megalops atlanticus*) caught from offshore waters of French Guiana. *Environmental Biology of Fishes* **99**(8–9), 593–602.

Rojas, Y. E. T., Osuna, F. P., Herrera, A. H., Magana, F. G., Garcia, S. A., Ortiz, H. V. & Sampson, L. (2014). Feeding grounds of juvenile scalloped hammerhead sharks (*Sphyrna lewini*) in the south-eastern Gulf of California. *Hydrobiologia* **726**(1), 81–94.

Ronnback, P., Macia, A., Almqvist, G., Schultz, L. & Troell, M. (2002). Do penaeid shrimps have a preference for mangrove habitats? Distribution pattern analysis on Inhaca Island, Mozambique. *Estuarine Coastal and Shelf Science* **55**(3), 427–436.

Rooker, J. R., Holt, G. J. & Holt, S. A. (1997). Condition of larval and juvenile red drum (*Sciaenops ocellatus*) from estuarine nursery habitats. *Marine Biology* **127**(3), 387–394.

Rooker, J. R., Holt, S. A., Holt, G. J. & Fuiman, L. A. (1999). Spatial and temporal variability in growth, mortality, and recruitment potential of postsettlement red drum, *Sciaenops ocellatus*, in a subtropical estuary. *Fishery Bulletin* **97**(3), 581–590.

Rooker, J. R., Landry, A. M., Geary, B. W. & Harper, J. A. (2004). Assessment of a shell bank and associated substrates as nursery habitat of postsettlement red snapper. *Estuarine Coastal and Shelf Science* **59**(4), 653–661.

Rooper, C. N., Armstrong, D. A. & Gunderson, D. R. (2002). *Habitat use by juvenile Dungeness crabs in coastal nursery estuaries* (Vol. 19, pp. 609-629). Fairbanks: Univ Alaska Sea Grant.

Rooper, C. N., Boldt, J. L., Batten, S. & Gburski, C. (2012). Growth and production of Pacific ocean perch (*Sebastes alutus*) in nursery habitats of the Gulf of Alaska. *Fisheries Oceanography* **21**(6), 415–429.

Rooper, C. N., Boldt, J. L. & Zimmermann, M. (2007). An assessment of juvenile Pacific Ocean perch (*Sebastes alutus*) habitat use in a deepwater nursery. *Estuarine Coastal and Shelf Science* **75**(3), 371–380.

Rooper, C. N., Gunderson, D. R. & Armstrong, D. A. (2003). Patterns in use of estuarine habitat by juvenile English sole (*Pleuronectes vetulus*) in four Eastern North Pacific estuaries. *Estuaries* **26**(4B), 1142–1154.

Rooper, C. N., Gunderson, D. R. & Armstrong, D. A. (2004). Application of the concentration hypothesis to English sole in nursery estuaries and potential contribution to coastal fisheries. *Estuaries* **27**(1), 102–111.

Rooper, C. N., Gunderson, D. R. & Armstrong, D. A. (2006). Evidence for resource partitioning and competition in nursery estuaries by juvenile flatfish in Oregon and Washington. *Fishery Bulletin* **104**(4), 616–622.

Rosenberg, A. A. (1982). Growth of juvenile english sole, *Parophrys vetulus*, in estuarine and open coastal nursery grounds. *Fishery Bulletin* **80**(2), 245–252.

Rosende-Pereiro, A. & Corgos, A. (2018). Pilot acoustic tracking study on young of the year scalloped hammerhead sharks, *Sphyrna lewini*, within a coastal nursery area in Jalisco, Mexico. *Latin American Journal of Aquatic Research* **46**(4), 645–659.

Ross, P. M., Thrush, S. F., Montgomery, J. C., Walker, J. W. & Parsons, D. M. (2007). Habitat complexity and predation risk determine juvenile snapper (*Pagrus auratus*) and goatfish (*Upeneichthys lineatus*) behaviour and distribution. *Marine and Freshwater Research* **58**(12), 1144–1151.

Ross, S. W. (2003). The relative value of different estuarine nursery areas in North Carolina for transient juvenile marine fishes. *Fishery Bulletin* **101**(2), 384–404.

Rothlisberg, P. C., Church, J. A. & Fandry, C. B. (1995). A mechanism for near-shore concentration and estuarine recruitment of post-larval *Penaeus plebejus* hess (Decapoda, Penaeidae). *Estuarine Coastal and Shelf Science* **40**(2), 115–138.

Rountree, R. A. & Able, K. W. (1992). Foraging habits, growth, and temporal patterns of salt-marsh creek habitat use by young-of-year summer flounder in New Jersey. *Transactions of the American Fisheries Society* **121**(6), 765–776.

Rountree, R. A. & Able, K. W. (1996). Seasonal abundance, growth, and foraging habits of juvenile smooth dogfish, *Mustelus canis*, in a New Jersey estuary. *Fishery Bulletin* **94**(3), 522–534.

Rowell, K., Flessa, K. W., Dettman, D. L. & Roman, M. (2005). The importance of Colorado River flow to nursery habitats of the Gulf corvina (*Cynoscion othonopterus*). *Canadian Journal of Fisheries and Aquatic Sciences* **62**(12), 2874–2885.

Rudershausen, P. J. & Buckel, J. A. (2020). Urbanization impacts on production and recruitment of *Fundulus heteroclitus* in salt marsh creeks. *Marine Ecology Progress Series* **645**, 187–204.

Rummer, J. L., Fangue, N. A., Jordan, H. L., Tiffany, B. N., Blansit, K. J., Galleher, S., Kirkpatrick, A., Kizlauskas, A. A., Pomory, C. M. & Bennett, W. A. (2009). Physiological tolerance to hyperthermia and hypoxia and effects on species richness and distribution of rockpool fishes of Loggerhead Key, Dry Tortugas National Park. *Journal of Experimental Marine Biology and Ecology* **371**(2), 155–162.

Ryer, C. H., Laurel, B. J. & Stoner, A. W. (2010). Testing the shallow water refuge hypothesis in flatfish nurseries. *Marine Ecology Progress Series* **415**, 275–282.

Ryer, C. H., Long, W. C., Spencer, M. L. & Iseri, P. (2015). Depth distribution, habitat associations, and differential growth of newly settled southern Tanner crab (*Chionoecetes bairdi*) in embayments around Kodiak Island, Alaska. *Fishery Bulletin* **113**(3), 256–269.

Ryer, C. H., Spencer, M. L., Iseri, P., Knoth, B. A., Laurel, B. J. & Stoner, A. W. (2013). Polychaete worm tubes modify juvenile northern rock sole *Lepidopsetta polyxystra* depth distribution in Kodiak nurseries. *Journal of Experimental Marine Biology and Ecology* **446**, 311–319.

Ryer, C. H., Stoner, A. W., Spencer, M. L. & Abookire, A. A. (2007). Presence of larger flatfish modifies habitat preference by Age-0 northern rock sole *Lepidopsetta polyxystra*. *Marine Ecology Progress Series* **342**, 227–238.

Saemundsson, K., Jonasson, J. P., Begg, G. A., Karlsson, H., Marteinsdottir, G. & Jonsdottir, I. G. (2020). Dispersal of juvenile cod (*Gadus morhua* L.) in Icelandic waters. *Fisheries Research* **232**, 105721.

Saier, B. (2000). Age-dependent zonation of the periwinkle *Littorina littorea* (L.) in the Wadden Sea. *Helgoland Marine Research* **54**(4), 224–229.

Sakamoto, T., van der Lingen, C. D., Shirai, K., Ishimura, T., Geja, Y., Peterson, J. & Komatsu, K. (2020). Otolith delta O-18 and microstructure analyses provide further evidence of population structure in sardine *Sardinops sagax* around South Africa. *ICES Journal of Marine Science* **77**(7–8), 2669–2680.

Sanchez, P., Demestre, M., Recasens, L., Maynou, F. & Martin, P. (2008). Combining GIS and GAMs to identify potential habitats of squid *Loligo vulgaris* in the Northwestern Mediterranean. *Hydrobiologia* **612**, 91–98.

Sanchez, R. P., Remeslo, A., Madirolas, A. & Deciechomski, J. D. (1995). Distribution and abundance of post-larvae and juveniles of the patagonian sprat, *Sprattus fuegensis*, and related hydrographic conditions. *Fisheries Research* **23**(1–2), 47–81.

Santos, R. O., Schinbeckler, R., Viadero, N., Larkin, M. F., Rennert, J. J., Shenker, J. M. & Rehage, J. S. (2019). Linking bonefish (*Albula vulpes*) populations to nearshore estuarine habitats using an otolith microchemistry approach. *Environmental Biology of Fishes* **102**(2), 267–283.

Sassa, C., Konishi, Y. & Mori, K. (2006). Distribution of jack mackerel (*Trachurus japonicus*) larvae and juveniles in the East China Sea, with special reference to the larval transport by the Kuroshio Current. *Fisheries Oceanography* **15**(6), 508–518.

Schaffler, J. J., van Montfrans, J., Jones, C. M. & Orth, R. J. (2013). Fish species distribution in seagrass habitats of Chesapeake Bay are structured by abiotic and biotic Factors. *Marine and Coastal Fisheries* **5**(1), 114–124.

Scharer, R. M., Stevens, P. W., Shea, C. P. & Poulakis, G. R. (2017). All nurseries are not created equal: large-scale habitat use patterns in two smalltooth sawfish nurseries. *Endangered Species Research* **34**, 473-492.

Schiel, D. R. (1993). Experimental evaluation of commercial-scale enhancement of abalone *Haliotis iris* populations in New Zealand. *Marine Ecology Progress Series* **97**(2), 167–181.

Schilling, H. T., Reis-Santos, P., Hughes, J. M., Smith, J. A., Everett, J. D., Stewart, J., Gillanders, B. M. & Suthers, I. M. (2018). Evaluating estuarine nursery use and life history patterns of *Pomatomus saltatrix* in eastern Australia. *Marine Ecology Progress Series* **598**, 187–199.

Schloesser, R. W. & Fabrizio, M. C. (2019). Nursery habitat quality assessed by the condition of juvenile fishes: not all estuarine areas are equal. *Estuaries and Coasts* **42**(2), 548–566.

Schmidt, A. J. & Diele, K. (2009). First field record of mangrove crab *Ucides cordatus* (Crustacea: Decapoda: Ucididae) recruits co-inhabiting burrows of conspecific crabs. *Zoologia* **26**(4), 792–794.

Schulz, K., Stevens, P. W., Hill, J. E., Trotter, A. A., Ritch, J. L., Williams, K. L., Patterson, J. T. & Tuckett, Q. M. (2020). Coastal wetland restoration improves habitat for juvenile sportfish in Tampa Bay, Florida,USA. *Restoration Ecology* **28**(5), 1283–1295.

Schwartzkopf, B. D. & Heppell, S. A. (2020). A feeding-ecology-based approach to evaluating nursery potential of estuaries for black rockfish. *Marine and Coastal Fisheries* **12**(2), 124–141.

Seabra, M. I., Hawkins, S. J., Espirito-Santo, C., Castro, J. J. & Cruz, T. (2020). Rock-pools as nurseries for co-existing limpets: Spatial and temporal patterns of limpet recruitment. *Regional Studies in Marine Science* **37**, 101339.

Searcy, S. P., Eggleston, D. B. & Hare, J. A. (2007). Is growth a reliable indicator of habitat quality and essential fish habitat for a juvenile estuarine fish? *Canadian Journal of Fisheries and Aquatic Sciences* **64**(4), 681–691.

Secor, D. H., Houde, E. D. & Kellogg, L. L. (2017). Estuarine retention and production of striped bass larvae: a mark-recapture experiment. *ICES Journal of Marine Science* **74**(6), 1735–1748.

Segura-Cobena, E., Alfaro-Shigueto, J., Mangel, J., Urzua, A. & Gorski, K. (2021). Stable isotope and fatty acid analyses reveal significant differences in trophic niches of smooth hammerhead *Sphyrna zygaena* (Carcharhiniformes) among three nursery areas in northern Humboldt Current System. *Peerj* **9**, e11283.

Seinor, K. M., Smith, S. D. A., Logan, M. & Purcell, S. W. (2020). Biophysical habitat features explain colonization and size distribution of introduced Trochus (Gastropoda). *Frontiers in Marine Science* **7**, 223.

Seitz, R. D., Knick, K. E. & Westphal, M. (2011). Diet selectivity of juvenile blue crabs (*Callinectes sapidus*) in Chesapeake Bay. *Integrative and Comparative Biology* **51**(4), 598–607.

Seitz, R. D., Lipcius, R. N., Knick, K. E., Seebo, M. S., Long, W. C., Brylawski, B. J. & Smith, A. (2008). Stock enhancement and carrying capacity of blue crab nursery habitats in Chesapeake Bay. *Reviews in Fisheries Science* **16**(1–3), 329–337.

Seitz, R. D., Lipcius, R. N. & Seebo, M. S. (2005). Food availability and growth of the blue crab in seagrass and unvegetated nurseries of Chesapeake Bay. *Journal of Experimental Marine Biology and Ecology* **319**(1–2), 57–68.

Selfati, M., El Ouamari, N., Lenfant, P., Fontcuberta, A., Lecaillon, G., Mesfioui, A., Boissery, P. & Bazairi, H. (2018). Promoting restoration of fish communities using artificial habitats in coastal marinas. *Biological Conservation* **219**, 89–95.

Selgrath, J. C., Hovel, K. A. & Wahle, R. A. (2007). Effects of habitat edges on American lobster abundance and survival. *Journal of Experimental Marine Biology and Ecology* **353**(2), 253–264.

Selleslagh, J. & Amara, R. (2013). Effect of starvation on condition and growth of juvenile plaice *Pleuronectes platessa*: nursery habitat quality assessment during the settlement period. *Journal of the Marine Biological Association of the United Kingdom* **93**(2), 479–488.

Serra-Pereira, B., Erzini, K., Maia, C. & Figueiredo, I. (2014). Identification of potential essential fish habitats for skates based on fishers' knowledge. *Environmental Management* **53**(5), 985–998.

Shahlapour, S., Bandpei, M. A. A., Rabbaniha, M., Pourang, N. & Nasrollahzadeh, H. (2019). Diversity and distribution of larval and juvenile fish in nearshore waters of the Southeastern Caspian Sea and Gorgan Bay. *Iranian Journal of Fisheries Sciences* **18**(2), 332–348.

Shakeri, L. M., Darnell, K. M., Carruthers, T. J. B. & Darnell, M. Z. (2020). Blue crab abundance and survival in a fragmenting coastal marsh system. *Estuaries and Coasts* **43**(6), 1545–1555.

Sharpe, C., Carr-Harris, C., Arbeider, M., Wilson, S. M. & Moore, J. W. (2019). Estuary habitat associations for juvenile Pacific salmon and pelagic fish: Implications for coastal planning processes. *Aquatic Conservation-Marine and Freshwater Ecosystems* **29**(10), 1636–1656.

Shaw, R. L., Curtis, T. H., Metzger, G., McCallister, M. P., Newton, A., Fischer, G. C. & Ajemian, M. J. (2021). Three-dimensional movements and habitat selection of young white sharks (*Carcharodon carcharias*) across a temperate continental shelf ecosystem. *Frontiers in Marine Science* **8**, 643831.

Shervette, V. R. & Gelwick, F. (2007). Habitat-speciric growth in juvenile pinfish. *Transactions of the American Fisheries Society* **136**(2), 445–451.

Shervette, V. R., Gelwick, F. & Hadley, N. (2011). Decapod utilization of adjacent oyster, vegetated marsh, and non-vegetated bottom habitats in a Gulf of Mexico estuary. *Journal of Crustacean Biology* **31**(4), 660–667.

Shervette, V. R., Perry, H. M., Rakocinski, C. F. & Biesiot, P. M. (2004). Factors influencing refuge occupation by stone crab *Menippe adina* juveniles in Mississippi Sound. *Journal of Crustacean Biology* **24**(4), 652–665.

Shideler, G. S., Sagarese, S. R., Harford, W. J., Schull, J. & Serafy, J. E. (2015). Assessing the suitability of mangrove habitats for juvenile Atlantic goliath grouper. *Environmental Biology of Fishes* **98**(10), 2067–2082.

Shoji, J. & Tanaka, M. (2007). Density-dependence in post-recruit Japanese seaperch *Lateolabrax japonicus* in the Chikugo River, Japan. *Marine Ecology Progress Series* **334**, 255–262.

Sievers, K. T., McClure, E. C., Abesamis, R. A. & Russ, G. R. (2020). Non-reef habitats in a tropical seascape affect density and biomass of fishes on coral reefs. *Ecology and Evolution* **10**(24), 13673–13686.

Silva, M. D., Araujo, F. G., de Azevedo, M. C. C. & Santos, J. N. D. (2004). The nursery function of sandy beaches in a Brazilian tropical bay for 0-group anchovies (Teleostei : Engraulidae): diel, seasonal and spatial patterns. *Journal of the Marine Biological Association of the United Kingdom* **84**(6), 1229–1232.

Simpfendorfer, C. A., Wiley, T. R. & Yeiser, B. G. (2010). Improving conservation planning for an endangered sawfish using data from acoustic telemetry. *Biological Conservation* **143**(6), 1460–1469.

Simpfendorfer, C. A., Yeiser, B. G., Wiley, T. R., Poulakis, G. R., Stevens, P. W. & Heupel, M. R. (2011). Environmental influences on the spatial ecology of juvenile smalltooth sawfish (*Pristis pectinata*): results from acoustic monitoring. *PLoS One* **6**(2), e16918.

Skilleter, G. A., Olds, A., Loneragan, N. R. & Zharikov, Y. (2005). The value of patches of intertidal seagrass to prawns depends on their proximity to mangroves. *Marine Biology* **147**(2), 353–365.

Sluis, M. Z., Barnett, B. K., Patterson, W. F., Cowan, J. H. & Shiller, A. M. (2015). Application of otolith chemical signatures to estimate population connectivity of red snapper in the western Gulf of Mexico. *Marine and Coastal Fisheries* **7**(1), 483–496.

Smale, M. J., Dicken, M. L. & Booth, A. J. (2015). Seasonality, behaviour and philopatry of spotted ragged-tooth sharks *Carcharias taurus* in Eastern Cape nursery areas, South Africa. *African Journal of Marine Science* **37**(2), 219–231.

Smith, G. C. & Parrish, J. D. (2002). Estuaries as nurseries for the jacks *Caranx ignobilis* and *Caranx melampygus* (Carangidae) in Hawaii. *Estuarine Coastal and Shelf Science* **55**(3), 347–359.

Smith, K. A. & Sinerchia, M. (2004). Timing of recruitment events, residence periods and post-settlement growth of juvenile fish in a seagrass nursery area, south-eastern Australia. *Environmental Biology of Fishes* **71**(1), 73–84.

Sobocinski, K. L. & Latour, R. J. (2015). Trophic transfer in seagrass systems: estimating seasonal production of an abundant seagrass fish, *Bairdiella chrysoura*, in lower Chesapeake Bay. *Marine Ecology Progress Series* **523**, 157–174.

Sogard, S. M. (1992). Variability in growth-rates of juvenile fishes in different estuarine habitats. *Marine Ecology Progress Series* **85**(1–2), 35–53.

Sogard, S. M., Able, K. W. & Hagan, S. M. (2001). Long-term assessment of settlement and growth of juvenile winter flounder (*Pseudopleuronectes americanus*) in New Jersey estuaries. *Journal of Sea Research* **45**(3–4), 189–204.

Sosa-Cordero, E., Arce, A. M., Aguilar-Davila, W. & Ramirez-Gonzalez, A. (1998). Artificial shelters for spiny lobster *Panulirus argus* (Latreille): an evaluation of occupancy in different benthic habitats. *Journal of Experimental Marine Biology and Ecology* **229**(1), 1–18.

Souza, A. T., Dias, E., Nogueira, A., Campos, J., Marques, J. C. & Martins, I. (2013). Population ecology and habitat preferences of juvenile flounder *Platichthys flesus* (Actinopterygii: Pleuronectidae) in a temperate estuary. *Journal of Sea Research* **79**, 60–69.

Spitzer, P. M., Mattila, J. & Heck, K. L. (2000). The effects of vegetation density on the relative growth rates of juvenile pinfish, *Lagodon rhomboides* (Linneaus), in Big Lagoon, Florida. *Journal of Experimental Marine Biology and Ecology* **244**(1), 67–86.

Staveley, T. A. B., Jacoby, D. M. P., Perry, D., van der Meijs, F., Lagenfelt, I., Cremle, M. & Gullstrom, M. (2019). Sea surface temperature dictates movement and habitat connectivity of Atlantic cod in a coastal fjord system. *Ecology and Evolution* **9**(16), 9076–9086.

Steller, D. L. & Caceres-Martinez, C. (2009). Coralline algal rhodoliths enhance larval settlement and early growth of the Pacific calico scallop *Argopecten ventricosus*. *Marine Ecology Progress Series* **396**, 49–60.

Stevens, B. G., Munk, J. E. & Cummiskey, P. A. (2004). Utilization of log-piling structures as artificial habitats for red king crab *Paralithodes camtschaticus*. *Journal of Shellfish Research* **23**(1), 221–226.

Stevens, P. W., Dutka-Gianelli, J., Nagid, E. J., Trotter, A. A., Johnson, K. G., Tuten, T. & Whittington, J. A. (2020). Niche partitioning among snook (Pisces: Centropomidae) in rivers of southeastern Florida and implications for species range limits. *Estuaries and Coasts* **43**(2), 396–408.

Steves, B. P., Cowen, R. K. & Malchoff, M. H. (2000). Settlement and nursery habitats for demersal fishes on the continental shelf of the New York Bight. *Fishery Bulletin* **98**(1), 167–188.

Stierhoff, K. L., Targett, T. E. & Power, J. H. (2009). Hypoxia-induced growth limitation of juvenile fishes in an estuarine nursery: assessment of small-scale temporal dynamics using RNA:DNA. *Canadian Journal of Fisheries and Aquatic Sciences* **66**(7), 1033–1047.

Stokesbury, K. D. E., Kirsch, J., Brown, E. D., Thomas, G. L. & Norcross, B. L. (2000). Spatial distributions of Pacific herring, *Clupea pallasi*, and walleye pollock, *Theragra chalcogramma*, in Prince William Sound, Alaska. *Fishery Bulletin* **98**(2), 400–409.

Stoner, A. W. & Lally, J. (1994). High-density aggregation in queen conch *Strombus gigas* - formation, patterns, and ecological significance. *Marine Ecology Progress Series* **106**(1–2), 73–84.

Stoner, A. W., Lin, J. & Hanisak, M. D. (1995). Relationships between seagrass bed characteristics and juvenile queen conch (*Strombus gigas* Linne) abundance in the Bahamas. *Journal of Shellfish Research* **14**(2), 315–323.

Stoner, A. W., Manderson, J. P. & Pessutti, J. P. (2001). Spatially explicit analysis of estuarine habitat for juvenile winter flounder: combining generalized additive models and geographic information systems. *Marine Ecology Progress Series* **213**, 253–271.

Stoner, A. W., Pitts, P. A. & Armstrong, R. A. (1996). Interaction of physical and biological factors in the large-scale distribution of juvenile queen conch in seagrass meadows. *Bulletin of Marine Science* **58**(1), 217–233.

Stoner, A. W., Ray-Culp, M. & O'Connell, S. M. (1998). Settlement and recruitment of queen conch, *Strombus gigas*, in seagrass meadows: Associations with habitat and micropredators. *Fishery Bulletin* **96**(4), 885–899.

Stoner, A. W. & Ray, M. (1993). Aggregation dynamics in juvenile queen conch (*Strombus gigas*) - population-structure, mortality, growth, and migration. *Marine Biology* **116**(4), 571–582.

Stoner, A. W. & Sandt, V. J. (1991). Experimental analysis of habitat quality for juvenile queen conch in seagrass meadows. *Fishery Bulletin* **89**(4), 693–700.

Stoner, A. W. & Titgen, R. H. (2003). Biological structures and bottom type influence habitat choices made by Alaska flatfishes. *Journal of Experimental Marine Biology and Ecology* **292**(1), 43–59.

Stoner, A. W. & Waite, J. M. (1991). Trophic biology of *Strombus gigas* in nursery habitats - diets and food sources in seagrass meadows. *Journal of Molluscan Studies* **57**, 451–460.

Stottrup, J. C., Munk, P., Kodama, M. & Stedmon, C. (2017). Changes in distributional patterns of plaice *Pleuronectes platessa* in the central and eastern North Sea; do declining nutrient loadings play a role? *Journal of Sea Research* **127**, 164–172.

Stowell, M. A., Copeman, L. A. & Ciannelli, L. (2019). Variability in juvenile English sole condition relative to temperature and trophic dynamics along an Oregon estuarine gradient. *Estuaries and Coasts* **42**(7), 1955–1968.

Strydom, N. A., Whitfield, A. K. & Paterson, A. W. (2002). Influence of altered freshwater flow regimes on abundance of larval and juvenile *Gilchristella aestuaria* (Pisces : Clupeidae) in the upper reaches of two South African estuaries. *Marine and Freshwater Research* **53**(2), 431–438.

Stunz, G. W., Minello, T. J. & Levin, P. S. (2002). A comparison of early juvenile red drum densities among various habitat types in Galveston Bay, Texas. *Estuaries* **25**(1), 76–85.

Subramaniam, S. P. (1990). Chwaka Bay (Zanzibar, East Africa) as a nursery ground for penaeid prawns. *Hydrobiologia* **208**(1–2), 111–122.

Sullivan, M. C., Cowen, R. K., Able, K. W. & Fahay, M. P. (2000). Spatial scaling of recruitment in four continental shelf fishes. *Marine Ecology Progress Series* **207**, 141–154.

Sullivan, M. C., Cowen, R. K., Able, K. W. & Fahay, M. P. (2003). Effects of anthropogenic and natural disturbance on a recently settled continental shelf flatfish. *Marine Ecology Progress Series* **260**, 237–253.

Suzuki, K. W., Kanematsu, Y., Nakayama, K. & Tanaka, M. (2014). Microdistribution and feeding dynamics of *Coilia nasus* (Engraulidae) larvae and juveniles in relation to the estuarine turbidity maximum of the macrotidal Chikugo River estuary, Ariake Sea, Japan. *Fisheries Oceanography* **23**(2), 157–171.

Swartzman, G., Winter, A., Coyle, K., Brodeur, R., Buckley, T., Ciannelli, L., Hunt, G., Ianelli, J. & Macklin, A. (2005). Relationship of age-0 pollock abundance and distribution around the Pribilof Islands, to other shelf regions of the eastern Bering Sea. *Fisheries Research* **74**(1–3), 273–287.

Switzer, T. S., MacDonald, T. C., McMichael, R. H. & Keenan, S. F. (2012). Recruitment of juvenile gags in the eastern Gulf of Mexico and factors contributing to observed spatial and temporal patterns of estuarine occupancy. *Transactions of the American Fisheries Society* **141**(3), 707–719.

Szedlmayer, S. T. & Able, K. W. (1993). Ultrasonic telemetry of age-0 summer flounder, *Paralichthys dentatus*, movements in a southern New Jersey estuary. *Copeia*(3), 728–736.

Szedlmayer, S. T., Able, K. W. & Rountree, R. A. (1992). Growth and temperature-induced mortality of young-of-the-year summer flounder (*Paralichthys dentatus*) in southern New Jersey. *Copeia*(1), 120–128.

Szedlmayer, S. T. & Lee, J. D. (2004). Diet shifts of juvenile red snapper (*Lutjanus compechanus*) with changes in habitat and fish size. *Fishery Bulletin* **102**(2), 366–375.

Tableau, A., Le Bris, H. & Brind'Amour, A. (2015). Available Benthic Energy Coefficient (ABEC): a generic tool to estimate the food profitability in coastal fish nurseries. *Marine Ecology Progress Series* **522**, 203–218.

Takahashi, M., Watanabe, Y., Kinoshita, T. & Watanabe, C. (2001). Growth of larval and early juvenile Japanese anchovy, *Engraulis japonicus*, in the Kuroshio-Oyashio transition region. *Fisheries Oceanography* **10**(2), 235–247.

Takami, H., Kawamura, T., Won, N. I., Muraoka, D., Hayakawa, J. & Onitsuka, T. (2017). Effects of macroalgal expansion triggered by the 2011 earthquake and tsunami on recruitment density of juvenile abalone *Haliotis discus* hannai at Oshika Peninsula, northeastern Japan. *Fisheries Oceanography* **26**(2), 141–154.

Talman, S. G., Norkko, A., Thrush, S. F. & Hewitt, J. E. (2004). Habitat structure and the survival of juvenile scallops *Pecten novaezelandiae*: comparing predation in habitats with varying complexity. *Marine Ecology Progress Series* **269**, 197–207.

Tamburin, E., Elorriaga-Verplancken, F. R., Estupinan-Montano, C., Madigan, D. J., Sanchez-Gonzalez, A., Padilla, M. H., Wcisel, M. & Galvan-Magana, F. (2020). New insights into the trophic ecology of young white sharks (*Carcharodon carcharias*) in waters off the Baja California Peninsula, Mexico. *Marine Biology* **167**(5), 55.

Tang, F., Minch, T., Dinning, K., Martyniuk, C. J., Kilada, R. & Rochette, R. (2015). Size-at-age and body condition of juvenile American lobsters (*Homarus americanus*) living on cobble and mud in a mixed-bottom embayment in the Bay of Fundy. *Marine Biology* **162**(1), 69–79.

Tanner, S. E., Reis-Santos, P., Vasconcelos, R. P., Thorrold, S. R. & Cabral, H. N. (2013). Population connectivity of *Solea solea* and *Solea senegalensis* over time. *Journal of Sea Research* **76**, 82–88.

Tarpgaard, E., Mogensen, M., Gronkjaer, P. & Carl, J. (2005). Using short-term growth of enclosed 0-group European flounder, *Platichthys flesus*, to assess habitat quality in a Danish bay. *Journal of Applied Ichthyology* **21**(1), 53–63.

Taylor, D. L. & Fehon, M. M. (2021). Blue crab (*Callinectes sapidus*) population structure in southern New England tidal rivers: patterns of shallow-water, unvegetated habitat use and quality. *Estuaries and Coasts* **44**(5), 1320–1343.

Taylor, D. L., McNamee, J., Lake, J., Gervasi, C. L. & Palance, D. G. (2016*a*). Juvenile winter flounder (*Pseudopleuronectes americanus*) and summer flounder (*Paralichthys dentatus*) utilization of southern New England nurseries: comparisons among estuarine, tidal river, and coastal lagoon shallow-water habitats. *Estuaries and Coasts* **39**(5), 1505–1525.

Taylor, M. D., Becker, A., Moltschaniwskyj, N. A. & Gaston, T. F. (2018). Direct and indirect interactions between lower estuarine mangrove and saltmarsh habitats and a commercially important penaeid shrimp. *Estuaries and Coasts* **41**(3), 815–826.

Taylor, M. D., Fry, B., Becker, A. & Moltschaniwskyj, N. (2017*a*). Recruitment and connectivity influence the role of seagrass as a penaeid nursery habitat in a wave dominated estuary. *Science of the Total Environment* **584**, 622–630.

Taylor, M. D., Fry, B., Becker, A. & Moltschaniwskyj, N. (2017*b*). The role of connectivity and physicochemical conditions in effective habitat of two exploited penaeid species. *Ecological Indicators* **80**, 1–11.

Taylor, M. D., Smith, J. A., Boys, C. A. & Whitney, H. (2016*b*). A rapid approach to evaluate putative nursery sites for penaeid prawns. *Journal of Sea Research* **114**, 26–31.

Teal, L. R., de Leeuw, J. J., van der Veer, H. W. & Rijnsdorp, A. D. (2008). Effects of climate change on growth of 0-group sole and plaice. *Marine Ecology Progress Series* **358**, 219–230.

Temperoni, B., Massa, A. E., Derisio, C., Martos, P., Berghoff, C. & Vinas, M. D. (2018). Effect of nursery ground variability on condition of age 0+year *Merluccius hubbsi*. *Journal of Fish Biology* **93**(6), 1090–1101.

Thistle, M. E., Schneider, D. C., Gregory, R. S. & Wells, N. J. (2010). Fractal measures of habitat structure: maximum densities of juvenile cod occur at intermediate eelgrass complexity. *Marine Ecology Progress Series* **405**, 39–56.

Thomas, M. J., Peterson, M. L., Chapman, E. D., Fangue, N. A. & Klimley, A. P. (2019). Individual habitat use and behavior of acoustically-tagged juvenile green sturgeon in the Sacramento-San Joaquin Delta. *Environmental Biology of Fishes* **102**(8), 1025–1037.

Ticzon, V. S., Mumby, P. J., Samaniego, B. R., Bejarano-Chavarro, S. & David, L. T. (2012). Microhabitat use of juvenile coral reef fish in Palau. *Environmental Biology of Fishes* **95**(3), 355–370.

Tobin, D., Wright, P. J., Gibb, F. M. & Gibb, I. M. (2010). The importance of life stage to population connectivity in whiting (*Merlangius merlangus*) from the northern European shelf. *Marine Biology* **157**(5), 1063–1073.

Toledo, P., Darnaude, A. M., Niklitschek, E. J., Ojeda, V., Voue, R., Leiva, F. P., Labonne, M. & Canales-Aguirre, C. B. (2019). Partial migration and early size of southern hake *Merluccius australis*: a journey between estuarine and oceanic habitats off Northwest Patagonia. *ICES Journal of Marine Science* **76**(4), 1094–1106.

Tominaga, O., Watanobe, M., Hanyu, M., Domon, K., Watanabe, Y. & Takahashi, T. (2000). Distribution and movement of larvae, juvenile and young of the pointhead flounder *Hippoglossoides pinetorum* in Ishikari Bay and vicinity, Hokkaido. *Fisheries Science* **66**(3), 442–451.

Tomiyama, T., Katayama, S., Omori, M. & Honda, H. (2005). Importance of feeding on regenerable parts of prey for juvenile stone flounder *Platichthys bicoloratus* in estuarine habitats. *Journal of Sea Research* **53**(4), 297–308.

Tomiyama, T., Kusakabe, K., Otsuki, N., Yoshida, Y., Takahashi, S., Hata, M., Shoji, J. & Hori, M. (2018). Ontogenetic changes in the optimal temperature for growth of juvenile marbled flounder *Pseudopleuronectes yokohamae*. *Journal of Sea Research* **141**, 14–20.

Tomiyama, T. & Omori, M. (2008). Habitat selection of stone and starry flounders in an estuary in relation to feeding and survival. *Estuarine Coastal and Shelf Science* **79**(3), 475–482.

Tomiyama, T., Omori, M. & Minami, T. (2007). Feeding and growth of juvenile stone flounder in estuaries: generality and the importance of sublethal tissue cropping of benthic invertebrates. *Marine Biology* **151**(1), 365–376.

Tomiyama, T., Yamashita, Y. & Tanaka, M. (2009). Occurrence of juvenile Japanese flounder *Paralichthys olivaceus* in brackish estuaries. *Estuarine Coastal and Shelf Science* **85**(4), 661–665.

Tournois, J., Darnaude, A. M., Ferraton, F., Aliaume, C., Mercier, L. & McKenzie, D. J. (2017). Lagoon nurseries make a major contribution to adult populations of a highly prized coastal fish. *Limnology and Oceanography* **62**(3), 1219–1233.

Trape, S., Durand, J. D., Vigliola, L. & Panfili, J. (2017). Recruitment success and growth variability of mugilids in a West African estuary impacted by climate change. *Estuarine Coastal and Shelf Science* **198**, 53–62.

Tsagarakis, K., Machias, A., Somarakis, S., Giannoulaki, M., Palialexis, A. & Valavanis, V. D. (2008). Habitat discrimination of juvenile sardines in the Aegean Sea using remotely sensed environmental data. *Hydrobiologia* **612**, 215–223.

Tupper, M. (2007). Identification of nursery habitats for commercially valuable humphead wrasse *Cheilinus undulatus* and large groupers (Pisces : Serranidae) in Palau. *Marine Ecology Progress Series* **332**, 189–199.

Tupper, M. & Boutilier, R. G. (1995). Effects of habitat on settlement, growth, and postsettlement survival of Atlantic cod (*Gadus morhua*). *Canadian Journal of Fisheries and Aquatic Sciences* **52**(9), 1834–1841.

Tupper, M. & Boutilier, R. G. (1997). Effects of habitat on settlement, growth, predation risk and survival of a temperate reef fish. *Marine Ecology Progress Series* **151**(1–3), 225–236.

Turner, S. M. & Limburg, K. E. (2016). Juvenile river herring habitat use and marine emigration trends: comparing populations. *Oecologia* **180**(1), 77–89.

Tyler, R. M. & Targett, T. E. (2007). Juvenile weakfish *Cynoscion regalis* distribution in relation to diel-cycling dissolved oxygen in an estuarine tributary. *Marine Ecology Progress Series* **333**, 257–269.

van der Veer, H. W., Dapper, R. & Witte, J. I. J. (2001). The nursery function of the intertidal areas in the western Wadden Sea for 0-group sole *Solea solea* (L.). *Journal of Sea Research* **45**(3–4), 271–279.

van der Veer, H. W., Freitas, V., Koot, J., Witte, J. I. J. & Zuur, A. F. (2010). Food limitation in epibenthic species in temperate intertidal systems in summer: analysis of 0-group plaice *Pleuronectes platessa*. *Marine Ecology Progress Series* **416**, 215–227.

van der Veer, H. W., Koot, J., Aarts, G., Dekker, R., Diderich, W., Freitas, V. & Witte, J. I. J. (2011). Long-term trends in juvenile flatfish indicate a dramatic reduction in nursery function of the Balgzand intertidal, Dutch Wadden Sea. *Marine Ecology Progress Series* **434**, 143–154.

van Montfrans, J., Ryer, C. H. & Orth, R. J. (2003). Substrate selection by blue crab *Callinectes sapidus* megalopae and first juvenile instars. *Marine Ecology Progress Series* **260**, 209–217.

Vanalderweireldt, L., Sirois, P., Mingelbier, M. & Winkler, G. (2019*a*). Feeding ecology of early life stages of striped bass (*Morone saxatilis*) along an estuarine salinity-turbidity gradient, St. Lawrence Estuary, Canada. *Journal of Plankton Research* **41**(4), 507–520.

Vanalderweireldt, L., Winkler, G., Forget-Lacoursiere, E. L., Mingelbier, M. & Sirois, P. (2020). Habitat use by early life stages of the re-established striped bass and conspecific fish species along the St. Lawrence estuary. *Estuarine Coastal and Shelf Science* **237**, 106696.

Vanalderweireldt, L., Winkler, G., Mingelbier, M. & Sirois, P. (2019*b*). Early growth, mortality, and partial migration of striped bass (*Morone saxatili*s) larvae and juveniles in the St. Lawrence estuary, Canada. *ICES Journal of Marine Science* **76**(7), 2235–2246.

Vance, D. J., Haywood, M. D. E., Heales, D. S., Kenyon, R. A., Loneragan, N. R. & Pendrey, R. C. (1996). How far do prawns and fish move into mangroves? Distribution of juvenile banana prawns *Penaeus merguiensis* and fish in a tropical mangrove forest in northern Australia. *Marine Ecology Progress Series* **131**(1–3), 115–124.

Vance, D. J., Haywood, M. D. E., Heales, D. S., Kenyon, R. A., Loneragan, N. R. & Pendrey, R. C. (2002). Distribution of juvenile penaeid prawns in mangrove forests in a tropical Australian estuary, with particular reference to *Penaeus merguiensis*. *Marine Ecology Progress Series* **228**, 165–177.

Vance, D. J., Haywood, M. D. E. & Staples, D. J. (1990). Use of a mangrove estuary as a nursery area by postlarval and juvenile banana prawns, *Penaeus merguiensis* de Man, in Northern Australia. *Estuarine Coastal and Shelf Science* **31**(5), 689–701.

Vandendriessche, S., Messiaen, M., O'Flynn, S., Vincx, M. & Degraer, S. (2007). Hiding and feeding in floating seaweed: Floating seaweed clumps as possible refuges or feeding grounds for fishes. *Estuarine Coastal and Shelf Science* **71**(3–4), 691–703.

Vandeperre, F., Aires-da-Silva, A., Fontes, J., Santos, M., Santos, R. S. & Afonso, P. (2014). Movements of blue sharks (*Prionace glauca*) across their life history. *PLoS One* **9**(8), e103538.

Vandeperre, F., Aires-da-Silva, A., Lennert-Cody, C., Santos, R. S. & Afonso, P. (2016). Essential pelagic habitat of juvenile blue shark (*Prionace glauca*) inferred from telemetry data. *Limnology and Oceanography* **61**(5), 1605–1625.

Vasconcelos, R. P., Reis-Santos, P., Costa, M. J. & Cabral, H. N. (2011*a*). Connectivity between estuaries and marine environment: Integrating metrics to assess estuarine nursery function. *Ecological Indicators* **11**(5), 1123–1133.

Vasconcelos, R. P., Reis-Santos, P., Fonseca, V., Ruano, M., Tanner, S., Costa, M. J. & Cabral, H. N. (2009). Juvenile fish condition in estuarine nurseries along the Portuguese coast. *Estuarine Coastal and Shelf Science* **82**(1), 128–138.

Vasconcelos, R. P., Reis-Santos, P., Maia, A., Fonseca, V., Franca, S., Wouters, N., Costa, M. J. & Cabral, H. N. (2010). Nursery use patterns of commercially important marine fish species in estuarine systems along the Portuguese coast. *Estuarine Coastal and Shelf Science* **86**(4), 613–624.

Vasconcelos, R. P., Reis-Santos, P., Tanner, S., Maia, A., Latkoczy, C., Gunther, D., Costa, M. J. & Cabral, H. (2008). Evidence of estuarine nursery origin of five coastal fish species along the Portuguese coast through otolith elemental fingerprints. *Estuarine Coastal and Shelf Science* **79**(2), 317–327.

Veale, L. J., Coulson, P. G., Hall, N. G. & Potter, I. C. (2016). Biology of a marine estuarine-opportunist fish species in a microtidal estuary, including comparisons among decades and with coastal waters. *Marine and Freshwater Research* **67**(8), 1128–1140.

Ventura, D., Bonifazi, A., Lasinio, G. J., Gravina, M. F., Mancini, E. & Ardizzone, G. (2018). Can microscale habitat-related differences influence the abundance of ectoparasites ? Multiple evidences from two juvenile coastal fish (Perciformes: Sparidae). *Estuarine Coastal and Shelf Science* **209**, 110–122.

Ventura, D., Lasinio, G. J. & Ardizzone, G. (2015). Temporal partitioning of microhabitat use among four juvenile fish species of the genus *Diplodus* (Pisces: Perciformes, Sparidae). *Marine Ecology-an Evolutionary Perspective* **36**(4), 1013–1032.

Verdiell-Cubedo, D., Oliva-Paterna, F. J., Ruiz-Navarro, A. & Torralva, M. (2013). Assessing the nursery role for marine fish species in a hypersaline coastal lagoon (Mar Menor, Mediterranean Sea). *Marine Biology Research* **9**(8), 739–748.

Verweij, M. C., Nagelkerken, I., Hans, I., Ruseler, S. M. & Mason, P. R. D. (2008). Seagrass nurseries contribute to coral reef fish populations. *Limnology and Oceanography* **53**(4), 1540–1547.

Vinagre, C., Amara, R., Maia, A. & Cabral, H. N. (2008*a*). Latitudinal comparison of spawning season and growth of 0-group sole, *Solea solea* (L.). *Estuarine Coastal and Shelf Science* **78**(3), 521–528.

Vinagre, C. & Cabral, H. N. (2008). Prey consumption by the juvenile soles, *Solea solea* and *Solea senegalensi*s, in the Tagus estuary, Portugal. *Estuarine Coastal and Shelf Science* **78**(1), 45–50.

Vinagre, C., Ferreira, T., Matos, L., Costa, M. J. & Cabral, H. N. (2009*a*). Latitudinal gradients in growth and spawning of sea bass, *Dicentrarchus labrax*, and their relationship with temperature and photoperiod. *Estuarine Coastal and Shelf Science* **81**(3), 375–380.

Vinagre, C., Fonseca, V., Maia, A., Amara, R. & Cabral, H. (2008*b*). Habitat specific growth rates and condition indices for the sympatric soles *Solea solea* (Linnaeus, 1758) and *Solea senegalensis* Kaup 1858, in the Tagus estuary, Portugal, based on otolith daily increments and RNA-DNA ratio. *Journal of Applied Ichthyology* **24**(2), 163–169.

Vinagre, C., Franca, S. & Cabral, H. N. (2006*b*). Diel and semi-lunar patterns in the use of an intertidal mudflat by juveniles of Senegal sole, *Solea senegalensis*. *Estuarine Coastal and Shelf Science* **69**(1–2), 246–254.

Vinagre, C., Franca, S., Costa, M. J. & Cabral, H. N. (2005). Niche overlap between juvenile flatfishes, *Platichthys flesus* and *Solea solea*, in a southern European estuary and adjacent coastal waters. *Journal of Applied Ichthyology* **21**(2), 114–120.

Vinagre, C., Maia, A., Reis-Santos, P., Costa, M. J. & Cabral, H. N. (2009*b*). Small-scale distribution of *Solea solea* and *Solea senegalensis* juveniles in the Tagus estuary (Portugal). *Estuarine Coastal and Shelf Science* **81**(3), 296–300.

Vinagre, C., Salgado, J., Costa, M. J. & Cabral, H. N. (2008*c*). Nursery fidelity, food web interactions and primary sources of nutrition of the juveniles of *Solea solea* and *S. senegalensis* in the Tagus estuary (Portugal): A stable isotope approach. *Estuarine Coastal and Shelf Science* **76**(2), 255–264.

Wada, T., Kamiyama, K., Shimamura, S., Matsumoto, I., Mizuno, T. & Nemoto, Y. (2011). Habitat utilization, feeding, and growth of wild spotted halibut *Verasper variegatus* in a shallow brackish lagoon: Matsukawa-ura, northeastern Japan. *Fisheries Science* **77**(5), 785–793.

Wahle, R. A., Bergeron, C., Tremblay, J., Wilson, C., Burdett-Coutts, V., Comeau, M., Rochette, R., Lawton, P., Glenn, R. & Gibson, M. (2013). The geography and bathymetry of American lobster benthic recruitment as measured by diver-based suction sampling and passive collectors. *Marine Biology Research* **9**(1), 42–58.

Wahle, R. A., Dellinger, L., Olszewski, S. & Jekielek, P. (2015). American lobster nurseries of southern New England receding in the face of climate change. *ICES Journal of Marine Science* **72**, 69–78.

Wahle, R. A. & Steneck, R. S. (1991). Recruitment habitats and nursery grounds of the american lobster *Homarus americanus* - a demographic bottleneck. *Marine Ecology Progress Series* **69**(3), 231–243.

Walsh, S. J. (1992). Factors influencing distribution of juvenile yellowtail flounder (*Limanda ferruginea*) on the Grand Bank of Newfoundland. *Netherlands Journal of Sea Research* **29**(1–3), 193–203.

Ward, T. M., Staunton-Smith, J., Hoyle, S. & Halliday, I. A. (2003). Spawning patterns of four species of predominantly temperate pelagic fishes in the sub-tropical waters of southern Queensland. *Estuarine Coastal and Shelf Science* **56**(5–6), 1125–1140.

Ward-Paige, C. A., Britten, G. L., Bethea, D. M. & Carlson, J. K. (2015). Characterizing and predicting essential habitat features for juvenile coastal sharks. *Marine Ecology-an Evolutionary Perspective* **36**(3), 419–431.

Warren, M. A., Gregory, R. S., Laurel, B. J. & Snelgrove, P. V. R. (2010). Increasing density of juvenile Atlantic (*Gadus morhua*) and Greenland cod (*G. ogac*) in association with spatial expansion and recovery of eelgrass (*Zostera marina*) in a coastal nursery habitat. *Journal of Experimental Marine Biology and Ecology* **394**(1–2), 154–160.

Wasserman, R. J., Whitfield, A. K., Deyzel, S. H. P., James, N. C. & Hugo, S. (2020). Seagrass (*Zostera capensis*) bed development as a predictor of size structured abundance for a ubiquitous estuary-dependent marine fish species. *Estuarine Coastal and Shelf Science* **238**, 106694.

Watanabe, T. T., Sant'Anna, B. S., Hattori, G. Y. & Zara, F. J. (2014). Population biology and distribution of the portunid crab *Callinectes ornatus* (Decapoda: Brachyura) in an estuary-bay complex of southern Brazil. *Zoologia* **31**(4), 329–336.

Webb, S. & Kneib, R. T. (2004). Individual growth rates and movement of juvenile white shrimp (*Litopenaeus setiferus*) in a tidal marsh nursery. *Fishery Bulletin* **102**(2), 376–388.

Webb, S. R. & Kneib, R. T. (2002). Abundance and distribution of juvenile white shrimp *Litopenaeus setiferus* within a tidal marsh landscape. *Marine Ecology Progress Series* **232**, 213–223.

Wen, C. K. C., Pratchett, M. S., Almany, G. R. & Jones, G. P. (2013). Role of prey availability in microhabitat preferences of juvenile coral trout (Plectropomus: Serranidae). *Journal of Experimental Marine Biology and Ecology* **443**, 39–45.

Weng, K. C., O'Sullivan, J. B., Lowe, C. G., Winkler, C. E., Dewar, H. & Block, B. A. (2007). Movements, behavior and habitat preferences of juvenile white sharks *Carcharodon carcharias* in the eastern Pacific. *Marine Ecology Progress Series* **338**, 211–224.

Wenner, E. L. & Beatty, H. R. (1993). Utilization of shallow estuarine habitats in South Carolina, USA, by postlarval and juvenile stages of *Penaeus* spp (Decapoda, Penaeidae). *Journal of Crustacean Biology* **13**(2), 280–295.

Wennhage, H. & Pihl, L. (2001). Settlement patterns of newly settled plaice (*Pleuronectes platessa*) in a non-tidal Swedish fjord in relation to larval supply and benthic predators. *Marine Biology* **139**(5), 877–889.

Wennhage, H., Pihl, L. & Stal, J. (2007). Distribution and quality of plaice (*Pleuronectes platessa*) nursery grounds on the Swedish west coast. *Journal of Sea Research* **57**(2–3), 218–229.

Werry, J. M., Lee, S. Y., Lemckert, C. J. & Otway, N. M. (2012). Natural or artificial? Habitat-use by the bull shark, *Carcharhinus leucas*. *PLoS One* **7**(11), e49796.

Wheeland, L. J. & Morgan, M. J. (2020). Age-specific shifts in Greenland halibut (*Reinhardtius hippoglossoides*) distribution in response to changing ocean climate. *ICES Journal of Marine Science* **77**(1), 230–240.

Wibisono, E., Puggioni, G., Firmana, E. & Humphries, A. (2021). Identifying hotspots for spatial management of the Indonesian deep-slope demersal fishery. *Conservation Science and Practice* **3**(5), e356.

Williams, J. A., Holt, G. J., Robillard, M. M. R., Holt, S. A., Hensgen, G. & Stunz, G. W. (2016). Seagrass fragmentation impacts recruitment dynamics of estuarine-dependent fish. *Journal of Experimental Marine Biology and Ecology* **479**, 97–105.

Wilson, J. K., Adams, A. J. & Ahrens, R. N. M. (2019). Atlantic tarpon (*Megalops atlanticus*) nursery habitats: evaluation of habitat quality and broad-scale habitat identification. *Environmental Biology of Fishes* **102**(2), 383–402.

Wilson, M. T., Brown, A. L. & Mier, K. L. (2005). Geographic variation among age-0 walleye pollock (*Theragra chalcogramma*): evidence of mesoscale variation in nursery quality? *Fishery Bulletin* **103**(1), 207–218.

Wilson, M. T., Buchheister, A. & Jump, C. (2011). Regional variation in the annual feeding cycle of juvenile walleye pollock (*Theragra chalcogramma*) in the western Gulf of Alaska. *Fishery Bulletin* **109**(3), 316–326.

Wilson, M. T., Mier, K. L. & Jump, C. M. (2013). Effect of region on the food-related benefits to age-0 walleye pollock (*Theragra chalcogramma*) in association with midwater habitat characteristics in the Gulf of Alaska. *ICES Journal of Marine Science* **70**(7), 1396–1407.

Wilson, S. K., Depczynski, M., Fulton, C. J., Holmes, T. H., Radford, B. T. & Tinkler, P. (2016). Influence of nursery microhabitats on the future abundance of a coral reef fish. *Proceedings of the Royal Society B-Biological Sciences* **283**(1836), 20160903.

Wilson, S. K., Depczynski, M., Holmes, T. H., Noble, M. M., Radford, B., Tinkler, P. & Fulton, C. J. (2017). Climatic conditions and nursery habitat quality provide indicators of reef fish recruitment strength. *Limnology and Oceanography* **62**(5), 1868–1880.

Wingate, R. L. & Secor, D. H. (2008). Effects of winter temperature and flow on a summer-fall nursery fish assemblage in the Chesapeake Bay, Maryland. *Transactions of the American Fisheries Society* **137**(4), 1147–1156.

Winner, B. L., Flaherty-Walia, K. E., Switzer, T. S. & Vecchio, J. L. (2014). Multidecadal evidence of recovery of nearshore red drum stocks off west-central florida and connectivity with inshore nurseries. *North American Journal of Fisheries Management* **34**(4), 780–794.

Woodland, R. J. & Secor, D. H. (2011). Differences in juvenile trophic niche for two coastal fish species that use marine and estuarine nursery habitats. *Marine Ecology Progress Series* **439**, 241–254.

Wright, P. J., Regnier, T., Gibb, F. M., Augley, J. & Devalla, S. (2018). Assessing the role of ontogenetic movement in maintaining population structure in fish using otolith microchemistry. *Ecology and Evolution* **8**(16), 7907–7920.

Wright, P. J., Tobin, D., Gibb, F. M. & Gibb, I. M. (2010). Assessing nursery contribution to recruitment: relevance of closed areas to haddock *Melanogrammus aeglefinus*. *Marine Ecology Progress Series* **400**, 221–232.

Wuenschel, M. J., Able, K. W., Buckel, J. A., Morley, J. W., Lankford, T., Branson, A. C., Conover, D. O., Drisco, D., Jordaan, A., Dunton, K., Secor, D. H., Woodland, R. J., Juanes, F. & Stormer, D. (2012). Recruitment patterns and habitat use of young-of-the-year bluefish along the United States east coast: insights from coordinated coastwide sampling. *Reviews in Fisheries Science* **20**(2), 80–102.

Yamada, H., Nanami, A., Ohta, I., Fukuoka, K., Sato, T., Kobayashi, M., Hirai, N., Chimura, M., Akita, Y. & Kawabata, Y. (2012). Occurrence and distribution during the post-settlement stage of two *Choerodon* species in shallow waters around Ishigaki Island, southern Japan. *Fisheries Science* **78**(4), 809–818.

Yamamoto, M., Makino, H., Kagawa, T. & Tominaga, O. (2004). Occurrence and distribution of larval and juvenile Japanese flounder *Paralichthys olivaceus* at sandy beaches in eastern Hiuchi-Nada, central Seto Inland Sea, Japan. *Fisheries Science* **70**(6), 1089–1097.

Yamamoto, M. & Tominaga, O. (2014). Prey availability and daily growth rate of juvenile Japanese flounder *Paralichthys olivaceus* at a sandy beach in the central Seto Inland Sea, Japan. *Fisheries Science* **80**(6), 1285–1292.

Yamashita, Y., Tominaga, O., Takami, H. & Yamada, H. (2003). Comparison of growth, feeding and cortisol level in *Platichthys bicoloratus* juveniles between estuarine and nearshore nursery grounds. *Journal of Fish Biology* **63**(3), 617–630.

Yates, P. M., Heupel, M. R., Tobin, A. J. & Simpfendorfer, C. A. (2015*a*). Ecological drivers of shark distributions along a tropical coastline. *PLoS One* **10**(4), e0121346.

Yates, P. M., Heupel, M. R., Tobin, A. J. & Simpfendorfer, C. A. (2015*b*). Spatio-temporal occurrence patterns of young sharks in tropical coastal waters. *Estuaries and Coasts* **38**(6), 2019–2030.

Yeager, L. A., Acevedo, C. L. & Layman, C. A. (2012). Effects of seascape context on condition, abundance, and secondary production of a coral reef fish, *Haemulon plumierii*. *Marine Ecology Progress Series* **462**, 231–240.

Yeager, M. E. & Hovel, K. A. (2017). Structural complexity and fish body size interactively affect habitat optimality. *Oecologia* **185**(2), 257–267.

Yeung, C. & Yang, M. S. (2017). Habitat quality of the coastal southeastern Bering Sea for juvenile flatfishes from the relationships between diet, body condition and prey availability. *Journal of Sea Research* **119**, 17–27.

Yeung, C. & Yang, M. S. (2018). Spatial variation in habitat quality for juvenile flatfish in the southeastern Bering Sea and its implications for productivity in a warming ecosystem. *Journal of Sea Research* **139**, 62–72.

Youcef, W. A., Lambert, Y. & Audet, C. (2013). Spatial distribution of Greenland halibut *Reinhardtius hippoglossoides* in relation to abundance and hypoxia in the estuary and Gulf of St. Lawrence. *Fisheries Oceanography* **22**(1), 41–60.

Yube, Y., Iseki, T., Hibino, M., Mizuno, K., Nakayama, K. & Tanaka, M. (2006). Daily age and food habits of *Lateolabrax latus* larvae and juveniles occurring in the innermost shallow waters of Uwajima Bay, Japan. *Fisheries Science* **72**(6), 1236–1249.

Zanella, I., Lopez-Garro, A. & Cure, K. (2019). Golfo Dulce: critical habitat and nursery area for juvenile scalloped hammerhead sharks *Sphyrna lewini* in the Eastern Tropical Pacific Seascape. *Environmental Biology of Fishes* **102**(10), 1291–1300.

Zapfe, G. A. & Rakocinski, C. R. (2008). Coherent growth and diet patterns in juvenile spot (*Leiostomus xanthurus* Lacepede) reflect effects of hydrology on access to shoreline habitat. *Fisheries Research* **91**(1), 107–111.

**Table S1.** Categories for methods used to measure abundance as a metric of juvenile habitat quality. Categories identified with an asterisk (*) were considered during the data extraction but no studies were found that used these measures. eDNA, environmental DNA; PIT, passive integrated transponder.

| **Code** | **Category** |
| --- | --- |
| ADJ | Adult–juvenile linkage (e.g. larval supply) |
| AER* | Aerial survey (drone/aircraft) |
| ATL | Acoustic telemetry |
| CAP | Capture count |
| COL | Collector count |
| HYD* | Hydrophone |
| IWC | In-water camera survey |
| IWD | In-water direct observer survey |
| MRP | Mark–recapture: PIT tag |
| MRV | Mark–recapture: visual tag |
| OTH | Other |
| PIT | Tracking with PIT tag |
| STL | Satellite telemetry |
| TRC* | Tracer abundance (e.g. eDNA, scales, otolith chemistry) |

**Table S2.** Categories for methods used to measure growth as a metric of juvenile habitat quality. Measures are categorised according to one of six focal processes and eight practical approaches. ‘Sum’ provides the total number of studies using a particular focal process or measure. Sums exceed the number of studies measuring growth because multiple approaches could be used in a single study. PIT, passive integrated transponder; W/L, weight to length.

| **Focal process** | **Sum** | **Code** | **Measure description** | **Practical approach** | **Sum** |
| --- | --- | --- | --- | --- | --- |
| Direct growth | 344 | SIZ | Size | Morphometric | 158 |
|  |  | SFP | Size–frequency progression | Morphometric | 89 |
|  |  | SAA | Size at age | Morphometric | 63 |
|  |  | MRV | Mark–recapture: visual tag | Morphometric | 18 |
|  |  | CAG | Growth increment from caged animals | Morphometric | 11 |
|  |  | MRP | Mark–recapture: PIT tag | Morphometric | 4 |
|  |  | IMP | Intermoult period | Morphometric | 1 |
| Feeding | 171 | DID | Diet (direct identification) | Macroscopic | 102 |
|  |  | SIF | Stable isotope fractionation | Geochemical | 41 |
|  |  | GUT | Gut fullness | Macroscopic | 23 |
|  |  | PRE | Prey characteristics (e.g. prey abundance, prey energy content) | Environmental | 3 |
|  |  | DIM | Diet (metabarcoding) | Molecular | 2 |
| Proportional growth | 110 | OTI | Increment widths of otoliths or other structures (e.g. scales) | Morphometric | 74 |
|  |  | OTB | Back-calculated size or size progression with age from otoliths or other structures (e.g. scales) | Morphometric | 36 |
| Energy acquired | 75 | WLR | W/L ratio (e.g. 'Fultons') | Morphometric | 51 |
|  |  | LIP | Lipid extraction | Biochemical | 12 |
|  |  | HEP | Hepatosomatic index | Macroscopic | 5 |
|  |  | PRO | Protein extraction | Biochemical | 4 |
|  |  | ENE | Measurement of energy content of tissues | Biochemical | 1 |
|  |  | FAT | Fat meter | Electrical | 1 |
|  |  | WAT | Measurement of water content of tissues | Biochemical | 1 |
| Metabolic processes | 34 | RNA | Nucleic acid-based indices: includes RNA content, DNA content, RNA:DNA, RNA:protein, etc. | Biochemical | 30 |
|  |  | ENZ | Enzyme activity | Biochemical | 2 |
|  |  | GEN | Gene expression | Molecular | 1 |
|  |  | HOR | Hormone level | Biochemical | 1 |
| Derived | 2 | MOD | Bioenergetics model driven by field measurements | Various | 2 |

**Table S3.** Categories for methods used to measure survival as a metric of juvenile habitat quality. Categories identified with an asterisk (*) were considered during the data extraction but no studies were found that used these measures.

| **Code** | **Category** |
| --- | --- |
| CAG | Caging study |
| CAR | Carcasses remaining |
| COH | Cohort analysis |
| DAM | Damage accrued |
| IWC | Direct observation of mortality events: in-water camera |
| IWD | Direct observation of mortality events: in-water observer |
| MRC | Mark–recapture |
| OTH* | Other |
| PRA | Predator abundance |
| PRS | Predator stomach contents |
| TTH | Tethering |

**Table S4.** Categories for methods used to measure juvenile–adult contribution as a metric of juvenile habitat quality. Categories identified with an asterisk (*) were considered during the data extraction but no studies were found that used these measures. PIT, passive integrated transponder.

| **Code** | **Category** | **Grouping** |
| --- | --- | --- |
| ABN | Abundance pattern | Abundance pattern |
| ATL | Acoustic telemetry | Artificial tagging |
| MRP | Mark–recapture: PIT tag | Artificial tagging |
| MRV | Mark–recapture: visual tag | Artificial tagging |
| NTI | Natural tag isotope | Natural tagging |
| NTM | Natural tag microchemistry | Natural tagging |
| NTP* | Natural tag parasite | Natural tagging |
| OTH* | Other | – |
| PIT | Tracking with PIT tag | Artificial tagging |
| STL | Satellite telemetry | Artificial tagging |
